# The largest meta-analysis on the global prevalence of microsporidia in mammals, avian and water provides insights into the epidemic features of these ubiquitous pathogens

**DOI:** 10.1186/s13071-021-04700-x

**Published:** 2021-04-01

**Authors:** Yingfei Ruan, Xiaofei Xu, Qiang He, Li Li, Junrui Guo, Jialing Bao, Guoqing Pan, Tian Li, Zeyang Zhou

**Affiliations:** 1 State Key Laboratory of Silkworm Genome Biology, Southwest University, Chongqing, 400715 China; 2 Chongqing Key Laboratory of Microsporidia Infection and Control, Southwest University, Chongqing, 400715 China; 3 College of Computer and Information Science, Southwest University, Chongqing, 400715 China; 4 College of Life Science, Chongqing Normal University, Chongqing, 400047 China

**Keywords:** Microsporidia, Microsporidiosis, Epidemiology, Global prevalence, Meta-analysis

## Abstract

**Background:**

Microsporidia are obligate intracellular parasites that can infect nearly all invertebrates and vertebrates, posing a threat to public health and causing large economic losses to animal industries such as those of honeybees, silkworms and shrimp. However, the global epidemiology of these pathogens is far from illuminated.

**Methods:**

Publications on microsporidian infections were obtained from PubMed, Science Direct and Web of Science and filtered according to the Newcastle-Ottawa Quality Assessment Scale. Infection data about pathogens, hosts, geography and sampling dates were manually retrieved from the publications and screened for high quality. Prevalence rates and risk factors for different pathogens and hosts were analyzed by conducting a meta-analysis. The geographic distribution and seasonal prevalence of microsporidian infections were drawn and summarized according to sampling locations and date, respectively.

**Results:**

Altogether, 287 out of 4129 publications up to 31 January 2020 were obtained and met the requirements, from which 385 epidemiological data records were retrieved and effective. The overall prevalence rates in humans, pigs, dogs, cats, cattle, sheep, nonhuman primates and fowl were 10.2% [2429/30,354; 95% confidence interval (CI) 9.2–11.2%], 39.3% (2709/5105; 95% CI 28.5–50.1%), 8.8% (228/2890; 95% CI 5.1–10.1%), 8.1% (112/1226; 95% CI 5.5–10.8%), 16.6% (2216/12,175; 95% CI 13.5–19.8%), 24.9% (1142/5967; 95% CI 18.6–31.1%), 18.5% (1388/7009; 95% CI 13.1–23.8%) and 7.8% (725/9243; 95% CI 6.4–9.2%), respectively. The higher prevalence in pigs suggests that routine detection of microsporidia in animals should be given more attention, considering their potential roles in zoonotic disease. The highest rate was detected in water, 58.5% (869/1351; 95% CI 41.6–75.5%), indicating that water is an important source of infections. Univariate regression analysis showed that CD4+ T cell counts and the living environment are significant risk factors for humans and nonhuman primates, respectively. Geographically, microsporidia have been widely found in 92 countries, among which Northern Europe and South Africa have the highest prevalence. In terms of seasonality, the most prevalent taxa, *Enterocytozoon bieneusi* and *Encephalitozoon*, display different prevalence trends, but no significant difference between seasons was observed. In addition to having a high prevalence, microsporidia are extremely divergent because 728 genotypes have been identified in 7 species. Although less investigated, microsporidia coinfections are more common with human immunodeficiency virus and *Cryptosporidium* than with other pathogens.

**Conclusions:**

This study provides the largest-scale meta-analysis to date on microsporidia prevalence in mammals, birds and water worldwide. The results suggest that microsporidia are highly divergent, widespread and prevalent in some animals and water and should be further investigated to better understand their epidemic features.

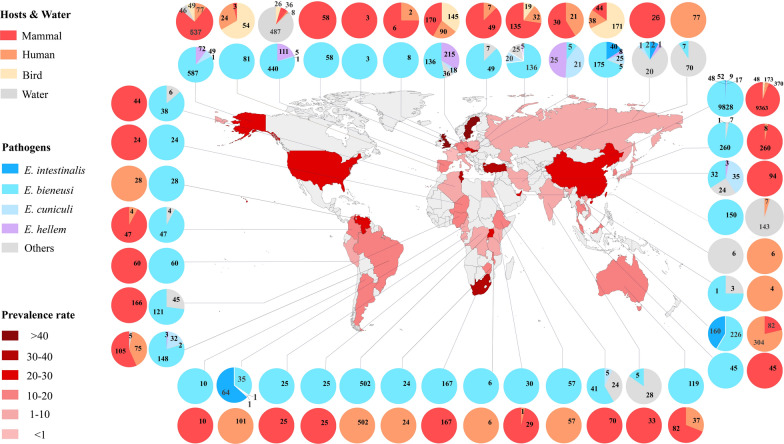

**Supplementary Information:**

The online version contains supplementary material available at 10.1186/s13071-021-04700-x.

## Introduction

Microsporidia are a group of ubiquitous and obligate intracellular pathogens [1–3]. Over 200 genera and 1400 species of microsporidia have been identified [4]. These pathogens have been widely reported to infect economically important insects, fish, crustaceans, mammals and birds [5–10]. Moreover, 17 species have been found to infect humans and cause microsporidiosis [3, 4]. *Enterocytozoon bieneusi* and *Encephalitozoon*, such as *Encephalitozoon cuniculi*, *Encephalitozoon intestinalis* and *Encephalitozoon hellem*, are the major species identified that infect humans, among which *E. bieneusi* is the most clinically reported [11, 12]. The clinical manifestations of human microsporidiosis are enteritis, cholecystitis and diffuse infection without specific symptoms [13]. Microsporidia can also cause self-limiting infections in immunocompetent individuals and life-threatening chronic diarrhea in immunocompromised populations [14]. Both immunocompetent and immunocompromised individuals run a risk of corneal infection, leading to self-limiting mild keratoconjunctivitis and even severe interstitial keratitis, which is difficult to treat with drugs [15, 16]. The infection rate of *E. bieneusi* in children < 2 years of age has been reported to be 13% in Nigeria [17], 17.4% in Uganda [18] and 11.83% in China [19]. In Australia, fecal samples from children < 3 years of age showed a higher infection rate (2.5%) than those from adults (0.3%) [19]. In addition, advanced age is also a potential risk factor. A study investigated 382 randomly selected people aged 1 to 84 years and showed that the infection rate in people > 50 (56.25%) was much higher than that in adults (38.55%) [16]. Another study surveyed *E. bieneusi* infection in 60 HIV-negative elderly patients and found that 8 were positive (17.02%) [20], which is higher than the overall rate of 11.8% in HIV-infected people [21]. Immunosuppressive therapy for organ and bone marrow transplant patients could lead to cellular immunodeficiency, which puts them at a high risk for microsporidian infection. In Poland, 11 out of 72 immunosuppressed renal transplant recipients were found to be infected by *E. bieneusi* [22]. To date, microsporidian infections have been observed in a wide range of human populations, including autoimmune diseases, end-stage renal failure, human immunodeficiency virus (HIV)-positive individuals, leukemia patients and travelers [12, 23]. In addition, studies have shown that there is no significant difference in microsporidia prevalence between genders [24].

Microsporidia seem to be ubiquitous and highly divergent in various naturally infected vertebrates [25]. Analysis of ribosomal ITS sequences revealed that some genotypes are present in both humans and animals, posing a public health threat [26–28]. Moreover, microsporidia have been detected in a variety of water sources, including irrigation water for crops, recreational water and wastewater from sewage treatment plants [17]. Studies have shown that the overall detection rate of *E. bieneusi* in water is 64.5% in China [29–31]. Researchers speculate that water is a possible container of microsporidia and provides a habitat for spores [32]. Because the chitin-containing spore wall provides protection against various environmental conditions and allows pathogens to survive for long periods, microsporidian spores from symptomatic and asymptomatic hosts could be the source of transmission in humans and animals [27, 32, 33]. Widespread microsporidia in animal hosts and water cause an important potential risk of human microsporidiosis. Therefore, understanding the epidemiology of microsporidia in animals and water is vital for developing effective measures to prevent the spread and infection of these pathogens. Herein, we conducted a systematic meta-analysis to assess the global prevalence of microsporidia.

## Materials and methods

### Data sources

Publications up to 31 January 2020 about microsporidia epidemiology were searched in the PubMed (https://pubmed.ncbi.nlm.nih.gov/), Science Direct (https://www.sciencedirect.com/) and Web of Science (https://apps.webofknowledge.com/) databases. The search results were manually checked and verified one by one. Terms used for searches were microsporidium, microsporidiosis, microsporidia, *Enterocytozoon*, *Encephalitozoon*, human, animal, bird, water, epidemiology and prevalence. Meanwhile, classification and genotype data on microsporidia were obtained from the nucleotide database of GenBank (https://www.ncbi.nlm.nih.gov/nuccore/) and searched using the terms microsporidia, ribosomal RNA, MS1, MS3, MS4 and MS7.

### Data processing

The included publications were required to investigate the prevalence of microsporidian infections. Data were excluded if they were from repeated studies and reviews, if there was no sample information or if the sample size was < 20, or if they were not determined with staining and molecular techniques. The suitability of all studies was assessed by four different authors. Disagreements were resolved by discussion among the authors.

We assessed the methodological quality of the included studies with an accessible full text according to the Newcastle-Ottawa Quality Assessment Scale [34]. One received a point if the study satisfied the following scoring guidelines: sample collection was random; sample size was > 200; reporting descriptive statistics to describe the population with proper measures of dispersion; reporting results without selectivity; repeating the detection using different methods. Up to five points could be assigned to a study. Publications with a total score of four or five points were regarded as high quality, whereas three points represented moderate quality and lower scores indicated low quality. Studies with a score of less than one point were excluded. After processing, the following data were extracted: country, sampling date, host, number of samples, number of positive samples, genus and species of the pathogen, age, gender and geographic region, and others are listed in Additional file 1: Tables S1–S9. In addition, information about microsporidian species, strains, genotypes, geographic locations and hosts was retrieved from the GenBank nucleotide database (Additional file 2).

### Data analysis

Meta-analysis was conducted using Stata version 15.0 to calculate the overall prevalence of microsporidian infections. The chi-squared test-based *Q* and *I*^2^ statistics were used to estimate the heterogeneity (*I*^2^ < 25%: low heterogeneity; 25% < *I*^2^ < 50%: moderate heterogeneity: *I*^2^ > 50%, high heterogeneity), which presents the percentage of variation between studies. A fixed effect model was used when heterogeneity was < 50%, and a random effects model was used when heterogeneity was > 50%. Due to the high heterogeneity (*I*^2^ > 50%, *P* < 0.1) in our study, random effects models were used for summary statistics. A forest plot was used to show proportions of individual studies and the total prevalence.

A potential source of heterogeneity was investigated by subgroup analysis and meta-regression analysis. The total prevalence and group-specific prevalence were considered among ages by comparing individuals < 18 years old and > 18 years old, genders by comparing males and females, geographical regions by comparing sub-Saharan Africa with other regions, income levels by comparing low-income countries with countries of other income levels and physical conditions by comparing individuals with HIV and other physical conditions. We also investigated the relationship between CD4+ T cell counts and diarrhea symptoms in the human host. For pig hosts, factors included age group by comparing post-weaned pigs with other ages and species group by comparing pigs with Tibetan pigs and wild boars. For cats and dogs, feral and domestic animals were used to compare living conditions. For cattle and sheep, species comparisons were conducted by comparing yaks and other species and sheep with goats. For nonhuman primates (NHPs), wild and domestic living environments were compared. For birds, factors included bird species by comparing water birds with terrestrial birds and living conditions by comparing wild and domestic living environments. We examined factors both individually and in multiple-variable models. Statistical techniques, *P* values and coefficients (95% CIs) were used to show the differences in factors.

We analyzed data according to the Preferred Reporting Items for Systematic Reviews and Meta-Analyses (PRISMA) statement [35], shown in Additional file 1: Table S10.

## Results

### Data content

**Fig. 1:**
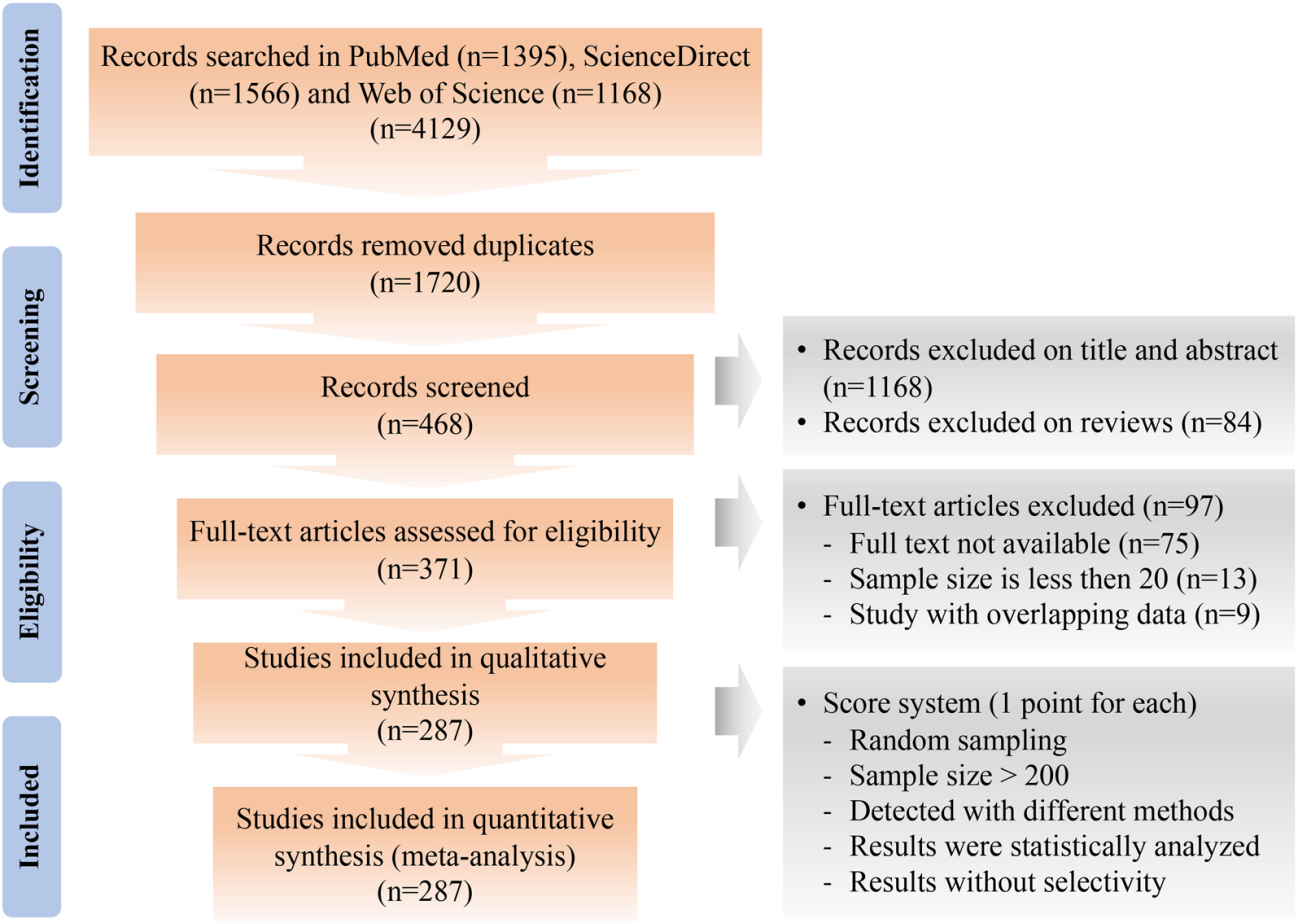
Flowchart for fetching and processing epidemiological data. Epidemiological publications on microsporidia were searched from databases and selected and scored according to the given criteria

In total, we searched 4129 studies and obtained 287 papers meeting the requirements (Fig. 1), from which 385 epidemiological data records were retrieved (Additional file 1: Tables S1–S9)*.* As the detailed prevalence data were predominantly from *E. bieneusi*, *E. cuniculi*, *E. hellem* and *E. intestinalis*, our subsequent meta-analysis mainly focused on these four species (Fig. 4b, c).

A total of 9488 records, including 1011 microsporidian species in 139 genera, were searched from the GenBank nucleotide database (Additional file 2: Table S11). *E. bieneusi* was the most commonly reported pathogen with 4322 records, followed by 2127 *Nosema* records, 443 *Dictyocoela* records, 351 *Vittaforma* records, 297 Microsporidia records, 286 *Encephalitozoon* records and 242 *Berwaldia* records (Fig. 4a).

### Global prevalence features of microsporidia

#### Prevalence of microsporidia and coinfection in humans

A total of 92 reports on human infections in 40 countries were retrieved, with 63 reports on *E. bieneusi* and 14 reports on *Encephalitozoon*. Regarding the sampling sources, 61 were HIV-positive patients, 22 were immunocompetent individuals, 7 were cancer patients, 7 were other patients and 5 were organ transplant individuals (Additional file 1: Table S1).

**Fig. 2:**
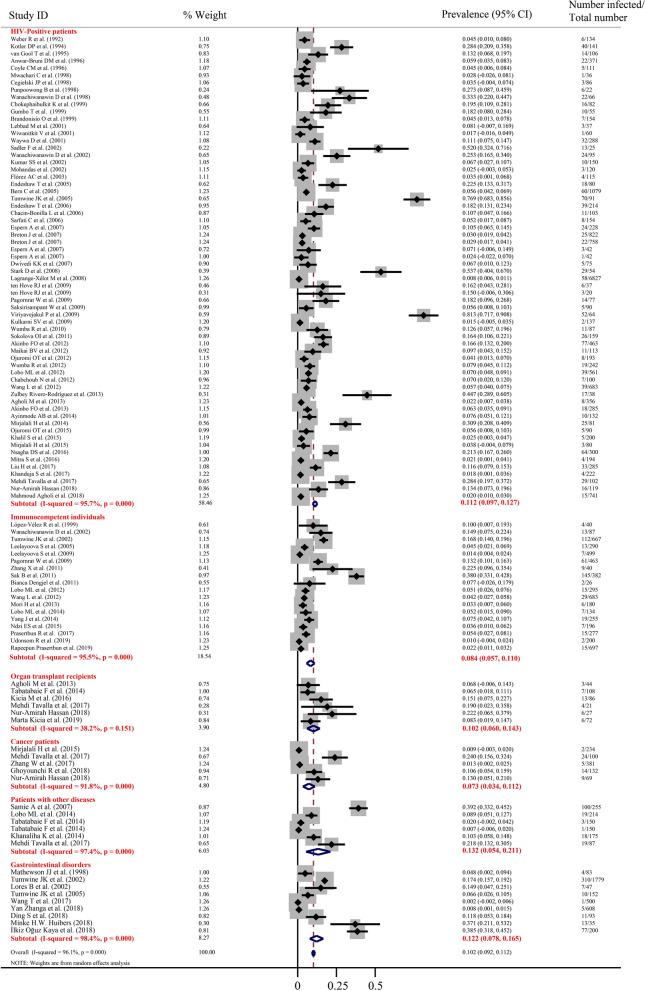
Forest plot diagram showing the prevalence of microsporidian infections in humans. The red items indicate the prevalence rate in different individuals and the 95% confidence interval (CI) in the considered studies based on the random effects model. The midpoint of each line shows the estimation of the prevalence, and the length of the line indicates the 95% CI of each study. The rhombic sign shows the combinational prevalence rate in corresponding studies

**Fig. 3:**
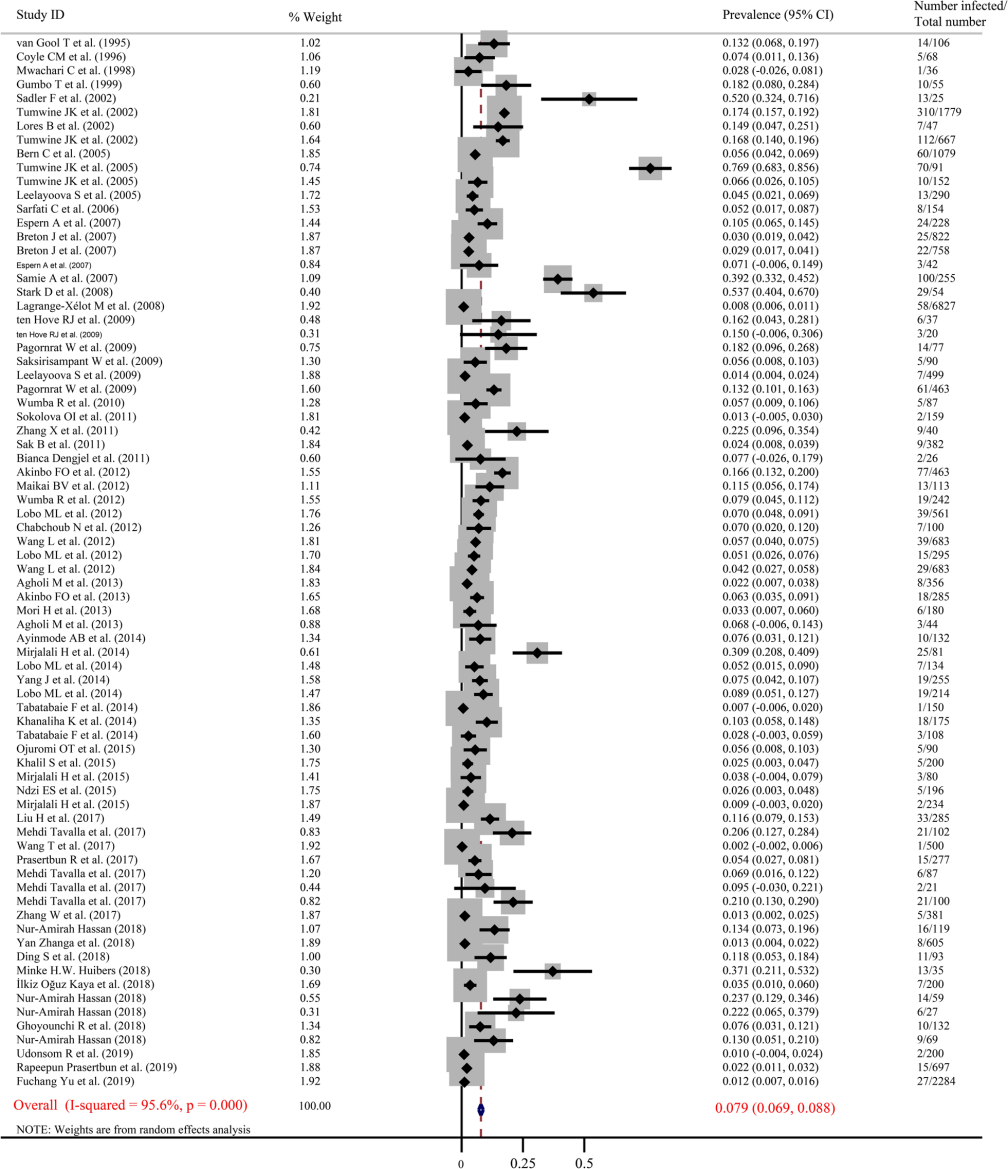
Forest plot diagram showing the prevalence of *E. bieneusi* infection in humans. The red item indicates the prevalence rate of *E. bieneusi* and the 95% confidence interval (CI) in the considered studies based on the random effects model. The midpoint of each line shows the estimation of the prevalence, and the length of the line indicates the 95% CI of each study. The rhombic sign shows the combinational prevalence rate in corresponding studies

**Fig. 4:**
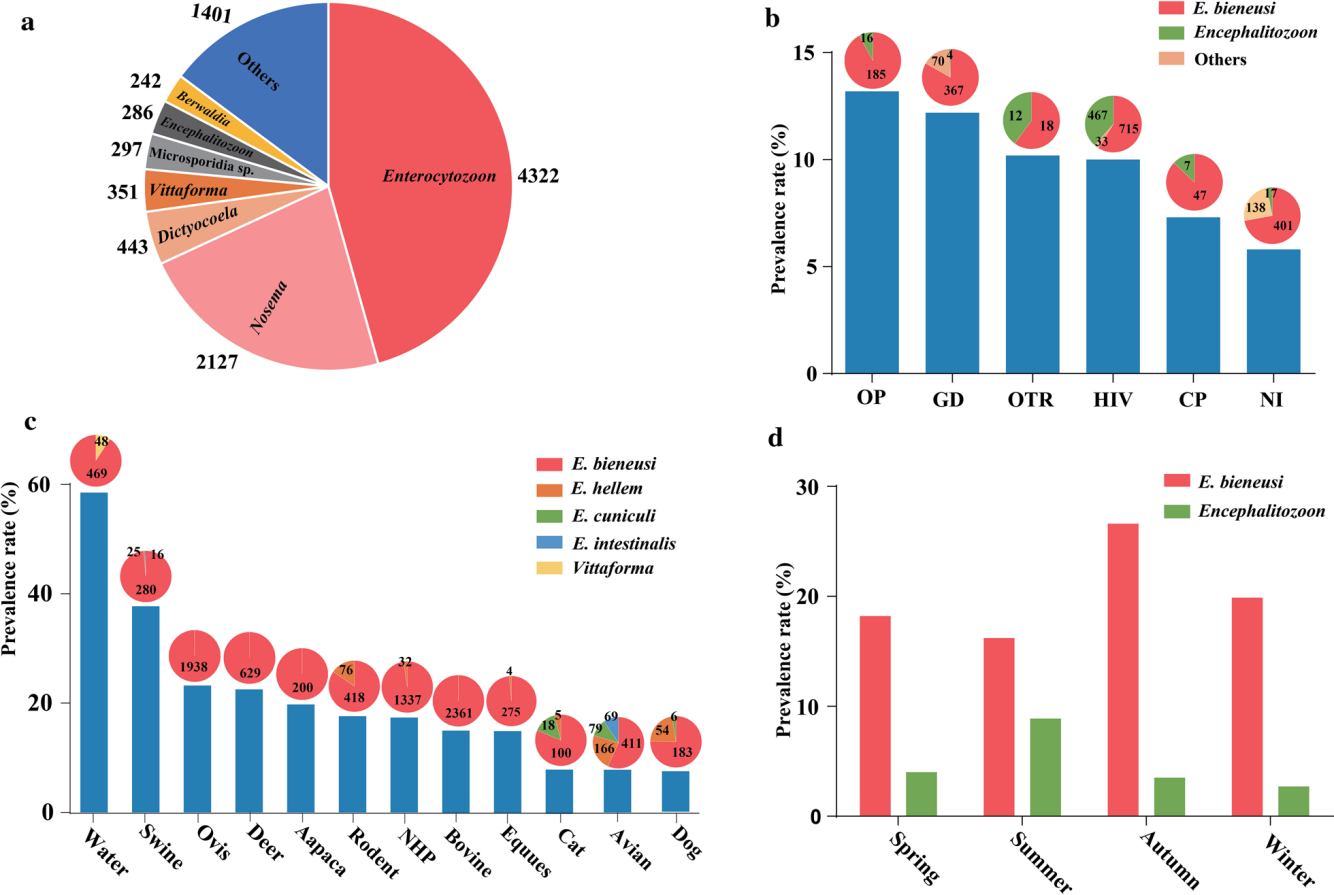
Summary of global microsporidia prevalence. **a** Microsporidian species reported and recorded in the GenBank nucleotide database. **b** Prevalence and distribution of microsporidia in humans under different physical conditions. The *Y*-axis represents the prevalence rate of microsporidia. The *X*-axis represents the type of physical conditions. *GD* gastrointestinal disorders, *OTR* organ transplant recipients, *HIV* HIV-positive patients, *CP* cancer patients, *OP* patients with other diseases, *NI* normal immunity individuals. The number on the pie charts represents the number of positive samples of microsporidia detected. The pie chart shows the pathogen compositions, where red indicates the number of *E. bieneusi*-positive individuals tested, blue indicates the number of microsporidia-positive individuals, and gray indicates the number of Encephalitozoon-positive individuals. **c** The prevalence and species of microsporidia in animal hosts and water. The pie chart shows pathogen composition. **d** Seasonal distribution of *E. bieneusi* and *Encephalitozoon* infections

The overall prevalence rate of microsporidian infection in humans using the random effects model in the meta-analysis was 10.2% (2429/30,354; 95% CI 9.2–11.2%) (Fig. 2), including 7.9% (1654/27,742; 95% CI 6.9–8.8%) by *E. bieneusi* and 10.9% (300/1886; 95% CI 6.2–15.6%) by *Encephalitozoon* (Fig. 3, Additional file 1: Figure S1). Seven factors related to human infections were examined, including gender, age, income level, region, physical condition, diarrhea symptoms and CD4+ T cell counts. The overall prevalence was 8.5% (276/7450; 95% CI 6.3–10.8%) in males and 7.1% (196/3697; 95% CI 5.2–8.9%) in females (Additional file 1: Figure S2). The overall prevalence in individuals < 18 years old was 7.5% (634/7807; 95% CI 6.7–10.9%) and 8.6% (536/6337; 95% CI 6.7–10.4%) in individuals > 18 years old (Additional file 1: Figure S3). The overall prevalence of microsporidia in HIV-positive patients was 11.2% (1190/19,740; 95% CI 9.7–12.7%) and 8.4% (548/5478; 95% CI 5.7–11.0%) in immunocompetent individuals, 7.3% (59/916; 95% CI 3.4–11.2%) in cancer patients, 10.2% (39/358; 95% CI 6.0–14.3%) in organ transplant recipients, 13.2% (160/881; 95% CI 5.4–21.1%) in other patients and 12.2% (438/3497; 95% CI 7.8–16.5%) in individuals with gastrointestinal disorders (Figs. 2, 4b). The prevalence rates for different geographic regions and income levels are shown in Table 1, Additional file 1: Figures S4, S5.

**Table 1:** Factors related to microsporidian infection in humans

Factors	No. studies	No. samples	No. positive samples	Overall prevalence (%) (95% CI)	Heterogeneity		Univariate meta-regression	
					*P*-value	*I*-squared (%)	*P*-value	Coefficient (95% CI) (%)
Gender							0.646	14.1 (1.48–26.8)
Male	21	7450	276	8.5 (6.3–10.8)	< 0.001	92.7		
Female	21	3697	196	8.1 (5.2–8.9)	< 0.001	93.0		
Age							0.687	11.6 (3.4–19.8)
< 18	21	7807	634	7.5 (6.7–10.9)	< 0.001	97.2		
> 18	33	6337	536	8.6 (6.7–10.4)	< 0.001	93.3		
Physical condition							0.888	14.2 (3.4–14.8)
HIV-positive patients	61	19,074	1190	11.2 (9.7–12.7)	< 0.001	95.7		
Immunocompetent individuals	21	5478	548	8.4 (5.7–11.0)	< 0.001	95.5		
Cancer patients	5	916	54	7.3 (3.4–11.2)	< 0.001	91.8		
Other patients	7	881	160	13.2 (5.4–21.1)	< 0.001	97.4		
Organ transplant recipients	7	358	39	10.2 (6.0–14.3)	< 0.001	38.2		
Gastrointestinal disorders individuals	9	3497	438	12.2 (7.8–16.5)	< 0.001	98.4		
Income level							0.459	9.58 (0.02–19.1)
Low income	10	3406	608	17.4 (11.7–23.1)	< 0.001	91.5		
Lower middle income	27	5240	396	6.9 (5.1–8.7)	< 0.001	89.9		
Upper middle income	50	15,036	1091	10.1 (8.6–11.6)	< 0.001	96.9		
High income	19	9913	428	12.6 (9.5–15.7)	< 0.001	96.6		
Region							0.602	14.8 (7.7–22.0)
Western and Central Europe and North America	18	3642	446	14.5 (9.8–19.1)	< 0.001	95.7		
Sub-Saharan Africa	27	19,360	616	12.8 (9.0–16.5)	< 0.001	97.0		
Asia and the Pacific	40	11,871	616	6.8 (5.5–8.0)	< 0.001	95.3		
Latin America and the Caribbean	4	256	32	16.8 (2.7–31)	< 0.001	92.7		
Middle East and North Africa	18	2890	243	9.7 (6.6–12.8)	< 0.001	93.3		
Eastern Europe and Central Asia	4	357	58	14.8 (7.8–21.7)	< 0.001	71.2		
Individual with diarrhea							0.011	34.0 (19.6–48.5)
Yes	22	2635	443	23.9 (16.8–31.0)	< 0.001	97.5		
Mix	8	2407	340	5.6 (2.3–8.9)	< 0.001	84.9		
No	8	1114	86	12.6 (6.3–18.7)	< 0.001	97.2		
CD4 counts ( cells/μl)							0.001	25.3 (11.7–38.9)
< 200	16	289	2840	13.1 (9.6–16.7)	< 0.001	91.2		
200–499	11	107	1166	6.8 (3.0–10.6)	< 0.001	89.1		
> 500	4	4	300	1.3 (0–2.6)	< 0.001	–		
Total	92	30,354	2429	10.2 (9.2–11.2)	< 0.001	96.1		

Due to substantial heterogeneity (*I*^2^ = 96.1%, *P* < 0.001; Table 1), meta-regression analysis indicated that CD4+ T cell counts (*P* = 0.001) and diarrhea (*P* = 0.011) might be sources of heterogeneity. Subgroup analysis showed that the overall prevalence rate of microsporidia in people with CD4+ T cell counts < 200 cells/μl was 13.1% (289/2840; 95% CI 9.6–16.7%), which was significantly higher than that in individuals with CD4+ T cell counts over 499 cells/μl, who had an overall prevalence rate of 1.3% (4/300; 95% CI 0–2.6%) (Additional file 1: Figure S6). In addition, the overall prevalence rate in individuals with diarrhea was 22.7% (448/2810; 95% CI 16.5–29.0%), which was much higher than that in individuals without diarrhea, with a prevalence rate of 12.5% (86/1114; 95% CI 6.3–18.7%) (Additional file 1: Figure S7). The infection rates showed no significant difference by gender (*P* = 0.646), age (*P* = 0.687), physical condition (*P* = 0.888), income level (*P* = 0.459) or geographic region (*P* = 0.602) (Table 1).

Nine publications reported coinfections of microsporidia with other pathogens (Additional file 1: Table S2): human immunodeficiency virus (HIV), *Cryptosporidium* spp., *Cyclospora cayetanensis* and *Mycobacterium tuberculosis*. Statistical analysis showed that the coinfection rates of microsporidia with HIV and *Cryptosporidium* spp. were 11.2% (1190/19,740; 95% CI 9.7–12.7%) and 8.0% (93/880; 95% CI 2.8–13.2%), respectively (Additional file 1: Figure S8). Only two cases reported the coinfection of microsporidia with *C. cayetanensis* with a coinfection rate from 1.69 to 1.72% [36, 37]. In addition, the coinfection rate of microsporidia with *M. tuberculosis* was 0.62% [38].

#### Prevalence of microsporidia in swine

In our searches, there were 19 and 2 reports on *E. bieneusi* and *Encephalitozoon* infections in swine from 12 countries, respectively (Additional file 1: Table S3). The infection rate was 3.7–92.6%. In detail, the prevalence rate was 39.3% (2709/5105; 95% CI 28.5–50.1%) in pigs, 32.9% (331/1132; 95% CI 15.0–50.8%) in wild boars and 32.4% (769/1801; 95% CI 3.8–61.0%) in Tibetan pigs (Additional file 1: Figure S9, Fig. 4c). In addition, the overall prevalence rate was 39.4% (439/1327; 95% CI 24.5–54.3%) in pre-weaned pigs, 50.7% (529/923; 95% CI 31.9–69.4%) in post-weaned pigs, 43.5% (576/1140; 95% CI 24.1–62.9%) in growing pigs and 33.5% (195/835; 95% CI 14.9–52.1%) in adult pigs (Additional file 1: Figure S10). The highest prevalence rate was shown in post-weaned pigs. Moreover, there was no significant difference between age groups (*P* > 0.05) (Table 2).

**Table 2:** Factors related to microsporidian infection in animals

Hosts	Factors	No. studies	No. samples	No. positive samples	Overall prevalence (%) (95% CI)	Heterogeneity		Univariate meta-regression	
						*P*-value	*I*-squared	*P*-value	Coefficient (95% CI) (%)
Swine	Species group							0.496	42.3 (22.0–62.6)
	Pigs	26	5105	2709	39.3 (28.5–50.1)	< 0.001	98.9		
	Wild boards	5	1132	331	32.9 (15.0–50.8)	< 0.001	97.8		
	Tibetan pigs	4	1801	769	32.4 (3.8–61)	< 0.001	99.4		
	Age group							0.707	46.6 (23.7–69.4)
	Preweaned pigs	11	1327	439	39.4 (24.5–54.3)	< 0.001	98.2		
	Postweaned pigs	10	923	529	50.7 (31.9–69.4)	< 0.001	97.8		
	Growing pigs	10	1140	576	43.5 (24.1–62.9)	< 0.001	98.4		
	Adult pigs	6	835	195	33.5 (14.9–52.1)	< 0.001	98.1		
Cat	Living environment							0.679	12.7 (0–24.4)
	Feral cats	5	313	31	9.1 (9.1–13)	< 0.001	27.7		
	Domestic cats	16	1016	92	8.1 (5.0–11.1)	< 0.001	74.3		
Dog	Living environment							0.753	12.0 (5.4–18.6)
	Feral dogs	5	490	58	10.3 (1.8–18.8)	< 0.001	97.0		
	Domestic dogs	12	2410	189	8.4 (5.4–11.5)	< 0.001	97.2		
Bos	Species group							0.027	22.1 (15.6–29.8)
	Cattle	33	12,175	2216	16.6 (13.5–19.8)	< 0.001	96.1		
	Yaks	3	924	65	4.9 (2.1–7.7)	< 0.001	91.9		
	Buffaloes	5	1335	80	15.1 (0.9–29.2)	< 0.001	94.3		
Ovis	Species group							0.485	30.2 (9.8–50.6)
	Sheep	18	5967	1142	24.9 (18.6–31.1)	< 0.001	98.1		
	Goats	10	3735	796	21.3 (9.8–32.8)	< 0.001	98.6		
NHPs	Living environment							0.016	34.9 (20.4–49.5)
	Farmed NHPs	14	3614	687	21.2 (15.3–27.2)	< 0.001	94.9		
	Wild NHPs	4	931	68	7.4 (2.4–12.4)	< 0.001	91.3		
Fowl	Living environment							0.116	34.9 (20.4–49.5)
	Domestic fowl	10	1578	235	14.4 (8.5–20.3)	< 0.001	92.9		
	Wild fowl	18	2318	499	21.9 (13.3–30.6)	< 0.001	96.7		
	Species group							0.891	17.0 (1.68–32.1)
	Amphibious fowl	3	971	91	16.4 (6.3–26.5)	< 0.001	92.2		
	Land fowl	16	2933	606	17.5 (12.7–22.4)	< 0.001	94.2		

#### Prevalence of microsporidia in cats and dogs

From the search results, we found 16 and 15 publications reporting infections of microsporidia in cats and dogs from 14 countries, respectively (Additional file 1: Table S4). The pooled prevalence of microsporidia in cats was 8.1% (112/1226; 95% CI 5.5–10.8%) (Additional file 1: Figure S11, Fig. 4c), including 9.1% (31/313; 95% CI 9.1–13%) in feral cats and 8.1% (92/1016; 95% CI 5.0–11.1%) in domestic cats (Additional file 1: Figure S12). The overall prevalence in dogs was estimated to be 8.8% (228/2890; 95% CI 5.1–10.1%) (Additional file 1: Figure S13, Fig. 4c). The estimated overall prevalence of microsporidia in feral and domestic dogs was 10.3% (58/480; 95% CI 1.8–18.8%) and 8.4% (189/2410; 95% CI 5.4–11.5%), respectively (Additional file 1: Figure S14). The prevalence of microsporidia in feral cats and dogs was higher than that in domestic cats, but the regression analysis showed no significant difference in cats (*P* = 0.679) and dogs (*P* = 0.753) living in different environmental conditions (Table 2).

#### Prevalence of microsporidia in ruminants

A total of 79 studies reported microsporidian infections in ruminants (Additional file 1: Table S5), among which cattle and sheep were investigated in 17 countries. The overall prevalence rate in bovines was 15.1% (2361/14,434; 95% CI 12.2–18.1%), among which the overall prevalence rates in cattle, yaks and water buffalos were 16.6% (2216/12,175; 95% CI 13.5–19.8%), 4.9% (57/347; 95% CI 2.1–7.7%) and 15.1% (57/347; 96% CI 0.9–29.2%), respectively (Additional file 1: Figure S15). Subgroup analysis showed that the pooled prevalence of microsporidian infection in cattle was significantly higher in yaks (*P* = 0.027) (Table 2). Because only three studies were performed in water buffalo, the prevalence of microsporidian infection in this species should be interpreted with caution. The overall prevalence rate in Ovis was 23.2% (1938/9702; 95% CI 18.4–28.0%). Sheep and goats of Ovis had pooled prevalence rates of 24.9% (1142/5967; 95% CI 18.6–31.1%) and 20.4% (796/3735; 95% CI 11.5–29.3%), respectively. In addition, the prevalence rate was 22.5% (629/3359; 95% CI 14.4–27.8%) in deer and 13.5% (210/1481; 95% CI 8.3–18.6%) in horses (Additional file 1: Figures S16–S18, Fig. 4c). The infection rate in camels was 20.5–45%, that in donkeys was 5.3–21.9%, and that in alpaca was 4.4–15.1% (Additional file 1: Table S5).

#### Prevalence of microsporidia in nonhuman primates (NHPs)

We searched 15 reports on microsporidian infections in NHPs from 16 countries. The infection rate in NHPs varied from 1.44% in *Pongo pygmaeus* to 67.8% in *Macaca fascicularis* (Additional file 1: Table S6). The overall prevalence rate acquired using the random effects model in the meta-analysis was 18.5% (1388/7009; 95% CI 13.1–23.8%) (Additional file 1: Figure S19, Fig. 4c). Subgroup analysis showed that the overall rates in wild and farmed NHPs were 7.4% (68/931; 95% CI 2.4–12.4%) and 21.2% (687/3614; 95% CI 15.3–27.2%), respectively, showing a significant difference between the groups (*P* < 0.005) (Additional file 1: Figure S20, Table 2).

#### Prevalence of microsporidia in avian

In total, we obtained 21 reports on microsporidian infections in avian (Additional file 1: Table S8). The meta-analysis showed that the prevalence rate in fowl was 7.8% (725/9243: 95% CI 6.4–9.2%). The overall prevalence rates of *E. bieneusi*, *E. cuniculi*, *E. hellem* and *E. intestinalis* in birds were estimated to be 13.8% (411/2961; 95% CI 9.7–18.0%), 4.4% (69/1662; 95% CI 1.8–7.0%), 7.7% (166/2628; 95% CI 4.9–10.6%) and 2.9% (68/1992; 95% CI 0.5–5.2%), respectively (Additional file 1: Figure S21). The meta-regression analysis showed that the pooled prevalence rate of *E. bieneusi* in birds was significantly higher than that of *E. intestinalis* (*P* = 0.002). Moreover, the infection rates in wild and domestic avians were 21.9% (449/2321; 95% CI 13.2–30.5%) and 14.4% (449/1578; 95% CI 8.5–20.3%), respectively (Additional file 1: Figure S22). The prevalence rates in land and amphibious birds were 17.5% (606/3022; 95% CI 12.5–22.4%) and 16.4% (91/971; 95% CI 6.3–26.5%), respectively (Additional file 1: Figure S23), showing no significant difference between the two groups (*P* > 0.05) (Table 2).

#### Prevalence of microsporidia in other mammals

The prevalence data of microsporidia in other mammals, such as rodents, foxes, raccoons, kangaroos, minks, takins and giant pandas, are shown in Additional file 1: Table S7. Determined with the random effects model in the meta-analysis, the overall prevalence rate in rodents was 17.6% (489/2870; 95% CI 11.6–23.7%) (Additional file 1: Figure S24). In detail, the prevalence rates in rabbits and pandas were 10.2–93% and 6–93%, respectively. In other mammalian populations, however, the overall prevalence could not be estimated because there were insufficient comparable investigations available for meta-analysis.

#### Microsporidia in water

In this study, there were 14 reports on microsporidia contamination in water (Additional file 1: Table S9). The overall prevalence rate of these parasites in water was 58.5% (869/1351; 95% CI 41.6–75.5%) (Additional file 1: Figure S25, Fig. 4c), while the highest rate reached 100% in a particular investigation [39]. Microsporidia have been identified in rivers, lakes, drinking water and wastewater. Subgroup analysis showed that the prevalence rate in wastewater treatment plants was 74.1% (485/630; 95% CI 61.9–86.3%), which was much higher than that in rivers and lakes (42.3%; 80/225; 95% CI 26.3–58.4%), although there was no significant difference (*P* > 0.05) (Additional file 1: Figure S26).

### Distributions of microsporidian infections

#### Geography of microsporidian infections

Data obtained from the GenBank nucleotide database showed that microsporidia are prevalent in 92 countries and regions, where pathogens have been mostly reported in China, Thailand, Russia and India, while investigations in Syria, Switzerland and Romania have been much less prevalent. In addition, microsporidia can infect at least 702 hosts.

**Fig. 5:**
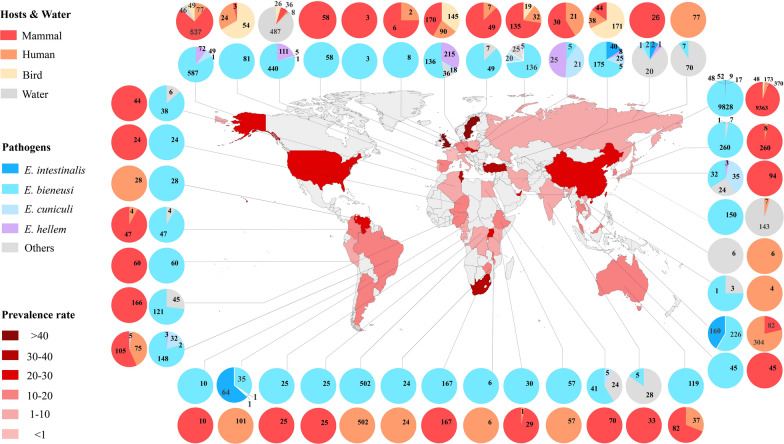
The global geographic prevalence of microsporidia. The overall prevalence of microsporidia in countries worldwide is indicated by color depth on a world map. The outer and inner pie charts show the overall infected hosts and hosts infected by different microsporidia in each country, respectively. The number on the outer and inner pie charts represents the number of positive samples of microsporidia detected in different hosts and pathogens.

Among all microsporidia, *E. bieneusi* is the most widespread species found in both humans and animals and has been detected in 42 countries and mainly reported in Poland, the USA and China. In China, *E. bieneusi* infections have been actively investigated and found in 148 hosts. In addition, *Nosema* is the second most reported and has been widely found in silkworms, wasps, mosquitoes and many other animals distributed in 42 countries, including Russia, Japan and Poland. Moreover, *Encephalitozoon* is widely found in individuals from Rwanda, Australia, Japan and 14 other countries (Additional file 2: Table S11). Among all countries investigated, Turkey, Malawi and Slovakia have the highest prevalence, while Western European countries, such as France, Russia and Italy, have a much lower prevalence (Fig. 5).

#### Seasonal prevalence of microsporidia

When calculating the seasonal prevalence rate of microsporidia, we found that *E. bieneusi* was higher in autumn and lower in summer, while *Encephalitozoon* was more prevalent in summer and less prevalent in winter (Fig. 4d). However, the prevalence rates between seasons showed no significant difference (*P* > 0.05).

#### Microsporidian genotypes

Currently, 722 microsporidian genotypes in 7 species have been identified, including 685 *E. bieneusi*, 14 *Loma salmonae*, 10 *E. hellem*, 8 *Loma* sp. SVB-PE3, 4 *E. cuniculi*, 3 *E. intestinalis* and 2 *Anncaliia algerae*, respectively. To date, the largest number of genotypes has been identified in *E. bieneusi*, among which genotype D has been the most commonly reported and has been found in 63 hosts, including human and domestic animals in 25 countries (Additional file 2: Table S11). The second largest *E. bieneusi* genotype, EbpC, has been found in 12 countries. Except for D and EbpC, some *E. bieneusi* genotypes are rare and have been reported only in one region. For example, HIN1 was only found in Nigeria [27]. Most microsporidia, such as *Nosema*, *Vairimorpha*, *Vittaforma* and *Paranucleospora*, lack genotype identification and need further study on genetic diversity.

## Discussion

This study summarized the global prevalence of microsporidian infections in animals and contamination of water, with overall rates of 15.5% (13,898/1621; 95% CI 14.6–16.4%) and 64.5% (1701/1621; 95% CI 51.9–77%), respectively. Among all microsporidia, *E. bieneusi* is the most commonly reported in mammals and birds, accounting for 48.6% of all epidemiological investigations. *Nosema* and *Dictyocoela* are the second most commonly reported, accounting for 22.4% and 4.6%, respectively (Fig. 4a). *Nosema* are widely found in honeybees and silkworms, and *Dictyocoela* are a common parasite in amphipods (Crustacea, Malacostraca) [29, 40].

Microsporidian infections in humans and animals have been reported worldwide in 40 and 32 countries, respectively (Additional file 2). However, surveys in water were only conducted in five countries. Considering the high prevalence rate in water, microsporidia investigations should be conducted in more water sources and locations. In addition, detection in wild animals is limited; for example, infectious data are lacking in elephants, peacocks, zebras, koalas and many other wild animals.

Microsporidian prevalence was reported to be related to sanitation facilities, drinking water, animal exposure and diagnostic methods [21, 29]. The high prevalence in Northern Europe and South Africa may be related to the developed logistics communication in Northern Europe and underdeveloped health facilities in South Africa (Fig. 5). Furthermore, the prevalence varies greatly in different areas of a country. In Nigeria, for instance, microsporidian infection in humans was 23.3% in Los Lagos [41] but only 7.5% in Ibadan [42]. Therefore, it is necessary to survey and compare the regional distributions in each country.

Notably, microsporidia are highly prevalent in water, posing a high risk for human and animal infection. Microsporidia have been detected in treated effluent and raw sewage [43]. In addition, the same genotypes were detected in wastewater from different treatment plants [30, 44, 45]. These findings suggested that water contamination was likely impacted by humans, livestock and rodents. Therefore, microsporidia from human and animal excretions entering the environment via sewage wastewater probably led to expansion of infection [21]. Because water is likely an important source of infection, guidelines on wastewater usage are needed to minimize human exposure to microsporidia. It is also necessary to strengthen the detection and disinfection of domestic water.

In addition to water, microsporidia can also be transmitted via food and air. Microsporidia have also been detected in fresh vegetables, fruits and milk [46]. The acceleration of food globalization and transportation could increase parasite transmission. Food-born microsporidia should receive increased attention [47]. Microsporidian spores are also present in air atomized from animal excrement, such as bird droppings, and could be an airborne pathogen [48]. Multiple microsporidian species have been detected in bird droppings [49, 50]. Our analysis also demonstrated that *E. bieneusi*, *E. cuniculi*, *E. hellem* and *E. intestinalis* have been widely found in birds. This implies that the dissemination of airborne microsporidia poses a risk of infection to humans.

Domestic animals showed higher infection rates and are another source of human microsporidiosis. The domestic pig, for example, showed the highest prevalence rate (Fig. 4c). In addition, some superior zoonotic genotypes, such as the D of *E. bieneusi*, have been widely identified in domestic cats, donkeys, cattle and pigs [51–54]. Indoor breeding and daily contact with these animals would increase the risk of zoonotic transmission.

The coinfections of microsporidia with other pathogens have been confirmed and should be considered an important public health problem. We found that the coinfection rates of microsporidia with HIV and *Cryptosporidium* were higher than those of microsporidia with other pathogens. Since 1985, the global AIDS pandemic has been a serious problem [55]. Infections by microsporidia and *Cryptosporidium* have been frequently reported in HIV-positive patients. Few studies, however, have examined the coinfections of microsporidia with other parasites.

In summary, this study systematically characterized the global prevalence of microsporidia, providing references for future epidemiological studies and pathogen control. However, more periodical surveys are needed to better understand the global and local epidemiological features of microsporidian infections.

## Supplementary Information

**Additional file 1: Figure S1.** Forest plot diagram showing the prevalence of *Encephalitozoon* infection in humans. **Figure S2.** Forest plot diagram showing microsporidian infection in humans of different genders. **Figure S3.** Forest plot diagram showing microsporidian infection in humans of different age groups. **Figure S4.** Forest plot diagram showing microsporidian infection in humans of different regions. **Figure S5.** Forest plot diagram showing microsporidian infection in humans of different income levels. **Figure S6.** Forest plot diagram showing microsporidian infection in humans with different CD4 cell counts. **Figure S7.** Forest plot diagram showing microsporidian infection in humans with diarrhea. **Figure S8.** Forest plot diagram showing the coinfection prevalence rate of microsporidia and Cryptosporidium in humans. **Figure S9.** Forest plot diagram showing the prevalence of microsporidian infection in different species of swine. **Figure S10.** Forest plot diagram showing the prevalence of microsporidian infection in different age groups of swine. **Figure S11.** Forest plot diagram showing the prevalence of microsporidian infection in cats. **Figure S12.** Forest plot diagram showing the prevalence of microsporidian infection in pet and feral cats. **Figure S13.** Forest plot diagram showing the prevalence of microsporidian infection in dogs. **Figure S14.** Forest plot diagram showing the prevalence of microsporidian infection in pet and feral dogs. **Figure S15.** Forest plot diagram showing the prevalence of microsporidian infection in Bos. **Figure S16.** Forest plot diagram showing the prevalence of microsporidian infection in Ovis. **Figure S17.** Forest plot diagram showing the prevalence of microsporidian infection in deer. **Figure S18.** Forest plot diagram showing the prevalence of microsporidian infection in horses. **Figure S19.** Forest plot diagram showing the prevalence of microsporidian infection in nonhuman primates. **Figure S20.** Forest plot diagram showing the prevalence of microsporidian infection in farm-raised and wild nonhuman primates. **Figure S21.** Forest plot diagram showing the prevalence of microsporidian infection in birds. **Figure S22.** Forest plot diagram showing the prevalence of microsporidian infection in wild and domestic birds. **Figure S23.** Forest plot diagram showing the prevalence of microsporidian infection in land and amphibious birds. **Figure S24.** Forest plot diagram showing the prevalence of microsporidian infection in rodents. **Figure S25.** Forest plot diagram showing the prevalence of microsporidian infection in water. **Figure S26.** Forest plot diagram showing the prevalence of microsporidian infection in different water sources. **Table S1.** Included studies of microsporidian infection in humans. **Table S2.** Included studies of microsporidian coinfection in humans. **Table S3.** Included studies of microsporidian infection in swine. **Table S4.** Included studies of microsporidian infection in cats and dogs. **Table S5.** Included studies of microsporidian infection in ruminants. **Table S6.** Included studies of microsporidian infection in nonhuman primates. **Table S7.** Included studies of microsporidian infection in other mammals. **Table S8.** Included studies of microsporidian infection in birds. **Table S9.** Included studies of microsporidia in water. **Table S10.** Checklist of items included when reporting a meta-analysis.

**Additional file 2: Table S11.** Information on microsporidian species, strains, genotypes, geographic locations and hosts was retrieved from the GenBank nucleotide database.

## Data Availability

All data generated or analyzed in this study are included in this article and its Additional files.

## References

[1] Santin M, Fayer R (2011). Microsporidiosis: *Enterocytozoon bieneusi* in domesticated and wild animals. Res Vet Sci.

[2] Vávra J, Ronny Larsson JI, Weiss LM, Becnel JJ (2014). Structure of microsporidia. Microsporidia: pathogens of opportunity.

[3] Oğuz Kaya İ, Doğruman Al F, Mumcuoğlu İ (2018). Investigation of microsporidia prevalence with calcofluor white and uvitex 2B chemiluminescence staining methods and molecular analysis of species in diarrheal patients. Mikrobiyol Bul.

[4] Fayer R, Santin-Duran M, Weiss LM, Becnel JJ (2014). Epidemiology of microsporidia in human infections. Microsporidia: pathogens of opportunity.

[5] Sinpoo C, Disayathanoowat T, Williams PH, Chantawannakul P (2019). Prevalence of infection by the microsporidian *Nosema* spp. in native bumblebees (*Bombus* spp.) in northern Thailand. PLoS ONE.

[6] He X, He X, Liu H, Li M, Cai S, Fu Z (2014). Proteomic analysis of BmN cells (*Bombyx mori*) in response to infection with *Nosema bombycis*. Acta Biochim Biophys Sin.

[7] Freeman MA, Yokoyama H, Osada A, Yoshida T, Yamanobe A, Ogawa K (2011). Spraguea (Microsporida: Spraguidae) infections in the nervous system of the Japanese anglerfish, *Lophius litulon* (Jordan), with comments on transmission routes and host pathology. J Fish Dis.

[8] Li DF, Zhang Y, Jiang YX, Xing JM, Tao DY, Zhao AY (2019). Genotyping and zoonotic potential of *Enterocytozoon bieneusi* in pigs in Xinjiang, China. Front Microbiol.

[9] Zhao W, Zhang W, Yang Z, Liu A, Zhang L, Yang F (2015). Genotyping of *Enterocytozoon bieneusi* in farmed blue foxes (*Alopex lagopus*) and raccoon dogs (*Nyctereutes procyonoides*) in China. PLoS ONE.

[10] Mathis A, Breitenmoser AC, Deplazes P (1999). Detection of new *Enterocytozoon* genotypes in faecal samples of farm dogs and a cat. Parasite.

[11] Thellier M, Breton J (2008). *Enterocytozoon bieneusi* in human and animals, focus on laboratory identification and molecular epidemiology. Parasite.

[12] Matos O, Lobo ML, Xiao L (2012). Epidemiology of *Enterocytozoon bieneusi* infection in humans. J Parasitol Res.

[13] Anane S, Attouchi H (2010). Microsporidiosis: epidemiology, clinical data and therapy. Gastroenterol Clin Biol.

[14] Stark D, van Hal S, Barratt J, Ellis J, Marriott D, Harkness J (2009). Limited genetic diversity among genotypes of *Enterocytozoon bieneusi* strains isolated from HIV-infected patients from Sydney, Australia. J Med Microbiol.

[15] Sharma S, Das S, Joseph J, Vemuganti GK, Murthy S (2011). Microsporidial keratitis: need for increased awareness. Surv Ophthalmol.

[16] Sak B, Brady D, Pelikánová M, Květoňová D, Rost M, Kostka M (2011). Unapparent microsporidial infection among immunocompetent humans in the Czech Republic. J Clin Microbiol.

[17] Maikai BV, Umoh JU, Lawal IA, Kudi AC, Ejembi CL, Xiao L (2012). Molecular characterizations of *Cryptosporidium*, *Giardia*, and *Enterocytozoon* in humans in Kaduna State, Nigeria. Exp Parasitol.

[18] Tumwine JK, Kekitiinwa A, Nabukeera N, Akiyoshi DE, Buckholt MA, Tzipori S (2002). *Enterocytozoon bieneusi* among children with diarrhea attending Mulago Hospital in Uganda. Am J Trop Med Hyg.

[19] Ding S, Huang W, Qin Q, Tang J, Liu H (2018). Genotype identification and phylogenetic analysis of *Enterocytozoon bieneusi* isolates from stool samples of diarrheic children. J Parasitol.

[20] Lores B, López-Miragaya I, Arias C, Fenoy S, Torres J, del Aguila C (2002). Intestinal microsporidiosis due to *Enterocytozoon bieneusi* in elderly human immunodeficiency virus—negative patients from Vigo, Spain. Clin Infect Dis.

[21] Wang ZD, Liu Q, Liu HH, Li S, Zhang L, Zhao YK (2018). Prevalence of *Cryptosporidium*, microsporidia and *Isospora* infection in HIV-infected people: a global systematic review and meta-analysis. Parasites Vectors.

[22] Kicia M, Szydłowicz M, Cebulski K, Jakuszko K, Piesiak P, Kowal A (2019). Symptomatic respiratory *Encephalitozoon cuniculi* infection in renal transplant recipients. Int J Infect Dis.

[23] Hassan NA, Lim YAL, Mahmud R, Mohd-Shaharuddin N, Wan Sulaiman WY, Ngui R (2018). Molecular diagnosis of microsporidia among immunocompromised patients in Kuala Lumpur, Malaysia. Am J Trop Med Hyg.

[24] Ghoyounchi R, Ahmadpour E, Spotin A, Mahami-Oskouei M, Rezamand A, Aminisani N (2017). Microsporidiosis in Iran: a systematic review and meta-analysis. Asian Pac J Trop Med.

[25] Li W, Feng Y, Santin M (2019). Host specificity of *Enterocytozoon bieneusi* and public health implications. Trends Parasitol.

[26] Yu F, Li D, Chang Y, Wu Y, Guo Z, Jia L (2019). Molecular characterization of three intestinal protozoans in hospitalized children with different disease backgrounds in Zhengzhou, central China. Parasites Vectors.

[27] Wang SS, Wang RJ, Fan XC, Liu TL, Zhang LX, Zhao GH (2018). Prevalence and genotypes of *Enterocytozoon bieneusi* in China. Acta Trop.

[28] Phrompraphai T, Itoh N, Iijima Y, Ito Y, Kimura Y (2019). Molecular detection and genotyping of *Enterocytozoon bieneusi* in family pet dogs obtained from different routes in Japan. Parasitol Int.

[29] Ben Ayed L, Yang W, Widmer G, Cama V, Ortega Y, Xiao L (2012). Survey and genetic characterization of wastewater in Tunisia for *Cryptosporidium* spp., *Giardia duodenalis*, *Enterocytozoon bieneusi*, *Cyclospora cayetanensis* and *Eimeria* spp.. J Water Health.

[30] Hu Y, Feng Y, Huang C, Xiao L (2014). Occurrence, source, and human infection potential of *Cryptosporidium* and *Enterocytozoon bieneusi* in drinking source water in Shanghai, China, during a pig carcass disposal incident. Environ Sci Technol.

[31] Galván AL, Magnet A, Izquierdo F, Fenoy S, Rueda C, Fernández Vadillo C (2013). Molecular characterization of human-pathogenic microsporidia and *Cyclospora cayetanensis* isolated from various water sources in Spain: a year-long longitudinal study. Appl Environ Microbiol.

[32] Didier ES, Stovall ME, Green LC, Brindley PJ, Sestak K, Didier PJ (2004). Epidemiology of microsporidiosis: sources and modes of transmission. Vet Parasitol.

[33] Waller T (1979). Sensitivity of *Encephalitozoon cuniculi* to various temperatures, disinfectants and drugs. Lab Anim.

[34] Stang A (2010). Critical evaluation of the Newcastle–Ottawa scale for the assessment of the quality of nonrandomized studies in meta-analyses. Eur J Epidemiol.

[35] Moher D, Liberati A, Tetzlaff J, Altman DG (2009). Preferred reporting items for systematic reviews and meta-analyses: the PRISMA statement. PLoS Med.

[36] Raccurt CP, Fouché B, Agnamey P, Menotti J, Chouaki T, Totet A (2008). Presence of *Enterocytozoon bieneusi* associated with intestinal coccidia in patients with chronic diarrhea visiting an HIV center in Haiti. Am J Trop Med Hyg.

[37] Kumar SS, Ananthan S, Lakshmi P (2002). Intestinal parasitic infection in HIV infected patients with diarrhoea in Chennai. Indian J Med Microbiol.

[38] Taghipour A, Tabarsi P, Sohrabi MR, Riahi SM, Rostami A, Mirjalali H (2019). Frequency, associated factors and clinical symptoms of intestinal parasites among tuberculosis and non-tuberculosis groups in Iran: a comparative cross-sectional study. Trans R Soc Trop Med Hyg.

[39] Huang C, Hu Y, Wang L, Wang Y, Li N, Guo Y (2017). Environmental transport of emerging human-pathogenic *Cryptosporidium* species and subtypes through combined sewer overflow and wastewater. Appl Environ Microbiol.

[40] Martín-Hernández R, Bartolomé C (2018). *Nosema ceranae* in *Apis mellifera*: a 12 years postdetection perspective. Environ Microbiol.

[41] Ojuromi OT, Izquierdo F, Fenoy S, Fagbenro-Beyioku A, Oyibo W, Akanmu A (2012). Identification and characterization of microsporidia from fecal samples of HIV-positive patients from Lagos, Nigeria. PLoS ONE.

[42] Ayinmode AB, Zhang H, Dada-Adegbola HO, Xiao L (2014). *Cryptosporidium hominis* subtypes and *Enterocytozoon bieneusi* genotypes in HIV-infected persons in Ibadan, Nigeria. Zoonoses Public Health.

[43] Yamashiro S, Fiuza V, Teixeira ÂTLS, Branco N, Levy CE, Castro I (2017). *Enterocytozoon bieneusi* detected by molecular methods in raw sewage and treated effluent from a combined system in Brazil. Mem Inst Oswaldo Cruz.

[44] Chen JS, Hsu BM, Tsai HC, Chen YP, Huang TY, Li KY (2018). Molecular surveillance of *Vittaforma*-like microsporidia by a small-volume procedure in drinking water source in Taiwan: evidence for diverse and emergent pathogens. Environ Sci Pollut Res Int.

[45] Li N, Xiao L, Wang L, Zhao S, Zhao X, Duan L (2012). Molecular surveillance of *Cryptosporidium* spp., *Giardia duodenalis*, and *Enterocytozoon bieneusi* by genotyping and subtyping parasites in wastewater. PLoS Negl Trop Dis.

[46] Jedrzejewski S, Graczyk TK, Slodkowicz-Kowalska A, Tamang L, Majewska AC (2007). Quantitative assessment of contamination of fresh food produce of various retail types by human-virulent microsporidian spores. Appl Environ Microbiol.

[47] Dixon BR, Gajadhar AA (2015). 13—Transmission dynamics of foodborne parasites on fresh produce. Foodborne parasites in the food supply web.

[48] Graczyk TK, Majewska AC, Schwab KJ (2008). The role of birds in dissemination of human waterborne enteropathogens. Trends Parasitol.

[49] Graczyk TK, Sunderland D, Rule AM, da Silva AJ, Moura IN, Tamang L (2007). Urban feral pigeons (*Columba livia*) as a source for air- and waterborne contamination with *Enterocytozoon bieneusi* spores. Appl Environ Microbiol.

[50] Haro M, Izquierdo F, Henriques-Gil N, Andrés I, Alonso F, Fenoy S (2005). First detection and genotyping of human-associated microsporidia in pigeons from urban parks. Appl Environ Microbiol.

[51] Prado JBF, Ramos C (2019). Occurrence of zoonotic *Enterocytozoon bieneusi* in cats in Brazil. Rev Bras Parasitol Vet.

[52] Yue DM, Ma JG, Li FC, Hou JL, Zheng WB, Zhao Q (2017). Occurrence of *Enterocytozoon bieneusi* in Donkeys (*Equus asinus*) in China: a public health concern. Front Microbiol.

[53] Udonsom R, Prasertbun R, Mahittikorn A, Chiabchalard R, Sutthikornchai C, Palasuwan A (2019). Identification of *Enterocytozoon bieneusi* in goats and cattle in Thailand. BMC Vet Res.

[54] Abe N, Kimata I (2010). Molecular survey of *Enterocytozoon bieneusi* in a Japanese porcine population. Vector Borne Zoonotic Dis.

[55] Miao YM, Gazzard BG (2000). Management of protozoal diarrhoea in HIV disease. HIV Med.

