# Supplementary material for: The largest meta-analysis on the global prevalence of microsporidia in mammals, avian and water provides insights into the epidemic features of these ubiquitous pathogens
Source: Parasit Vectors. 2021 Apr 1;14:186. doi: 10.1186/s13071-021-04700-x (PMC8017775; doi:10.1186/s13071-021-04700-x)
Supplement: Supplementary file 1 — Additional file 1: Figure S1. Forest plot diagram showing the prevalence of Encephalitozoon infection in humans. Figure S2. Forest plot diagram showing microsporidian infection in humans of different genders. Figure S3. Forest plot diagram showing microsporidian infection in humans of different age groups. Figure S4. Forest plot diagram showing microsporidian infection in humans of different regions. Figure S5. Forest plot diagram showing microsporidian infection in humans of different income levels. Figure S6. Forest plot diagram showing microsporidian infection in humans with different CD4 cell counts. Figure S7. Forest plot diagram showing microsporidian infection in humans with diarrhea. Figure S8. Forest plot diagram showing the coinfection prevalence rate of microsporidia and Cryptosporidium in humans. Figure S9. Forest plot diagram showing the prevalence of microsporidian infection in different species of swine. Figure S10. Forest plot diagram showing the prevalence of microsporidian infection in different age groups of swine. Figure S11. Forest plot diagram showing the prevalence of microsporidian infection in cats. Figure S12. Forest plot diagram showing the prevalence of microsporidian infection in pet and feral cats. Figure S13. Forest plot diagram showing the prevalence of microsporidian infection in dogs. Figure S14. Forest plot diagram showing the prevalence of microsporidian infection in pet and feral dogs. Figure S15. Forest plot diagram showing the prevalence of microsporidian infection in Bos. Figure S16. Forest plot diagram showing the prevalence of microsporidian infection in Ovis. Figure S17. Forest plot diagram showing the prevalence of microsporidian infection in deer. Figure S18. Forest plot diagram showing the prevalence of microsporidian infection in horses. Figure S19. Forest plot diagram showing the prevalence of microsporidian infection in nonhuman primates. Figure S20. Forest plot diagram showing the prevalence of microsporidian infecti [file 13071_2021_4700_MOESM1_ESM.docx]

Additional file 1

# Additional Figures and Tables

## Additional Figures


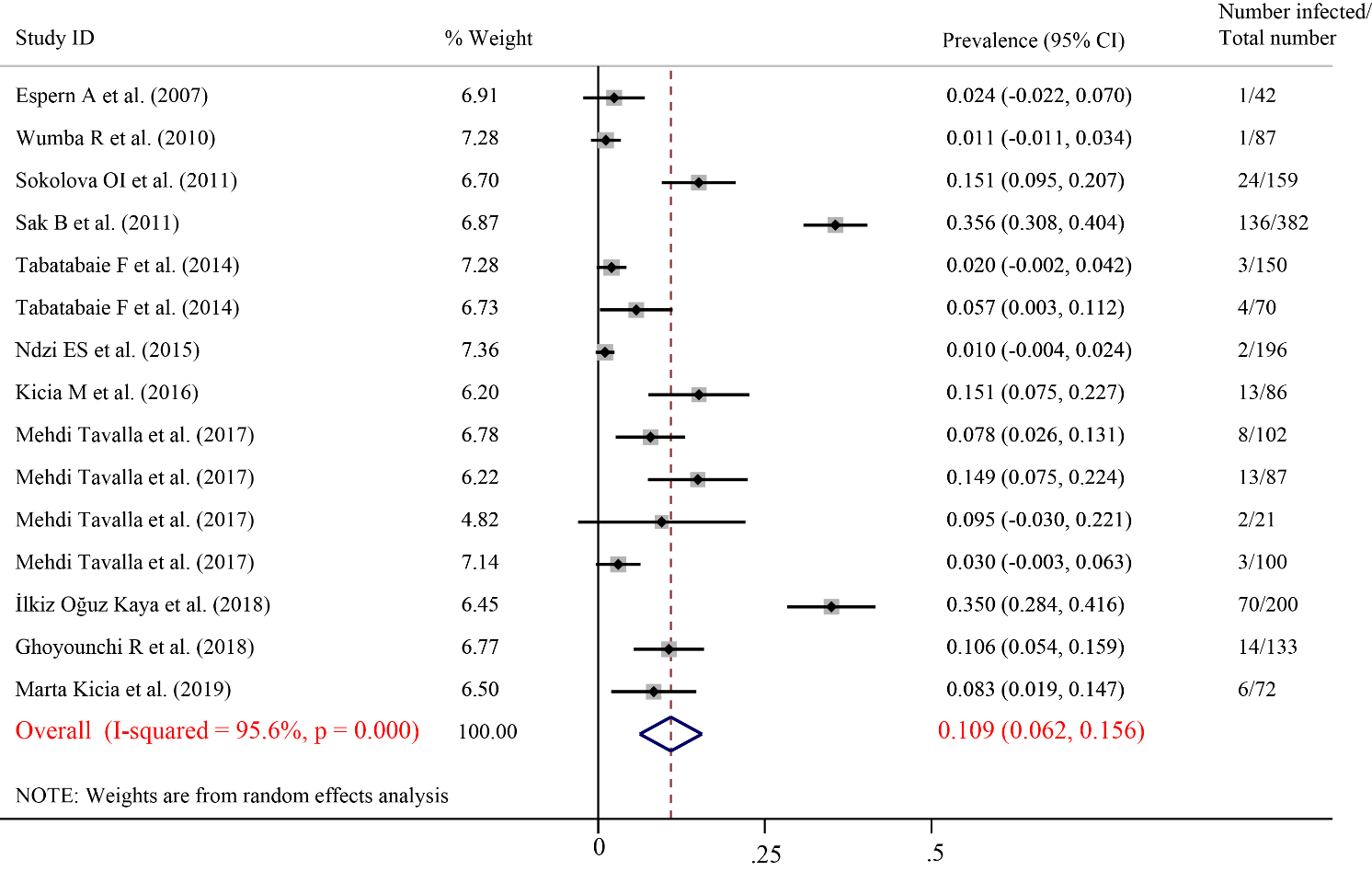


**Figure S1. Forest plot diagram showing the prevalence of *Encephalitozoon* infection in humans.** The red item indicates prevalence rate of *Encephalitozoon* and the 95% confidence interval (CI) in the considered studies based on the random effects model. The midpoint of each line shows the estimation of the prevalence and the length of the line indicates the 95% CI of each study. The rhombic sign shows the combinational prevalence rate in corresponding studies.


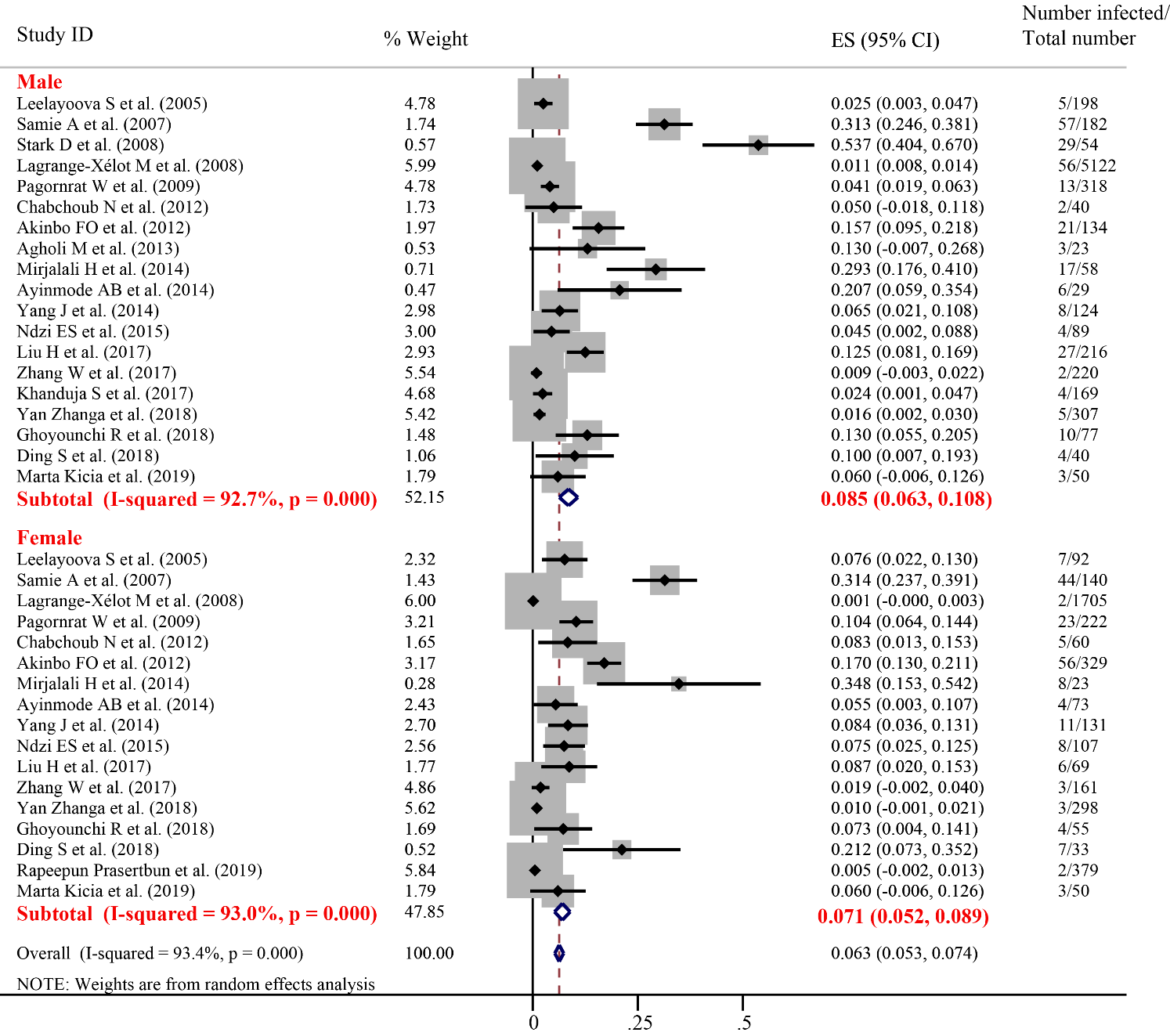


**Figure S2. Forest plot diagram showing microsporidian infection in humans of different genders.** The red items indicate microsporidia prevalence rate in male and female and the 95% confidence interval (CI) in the considered studies based on the random effects model. The midpoint of each line shows the estimation of the prevalence and the length of the line indicates the 95% CI of each study. The rhombic sign shows the combinational prevalence rate in corresponding studies.


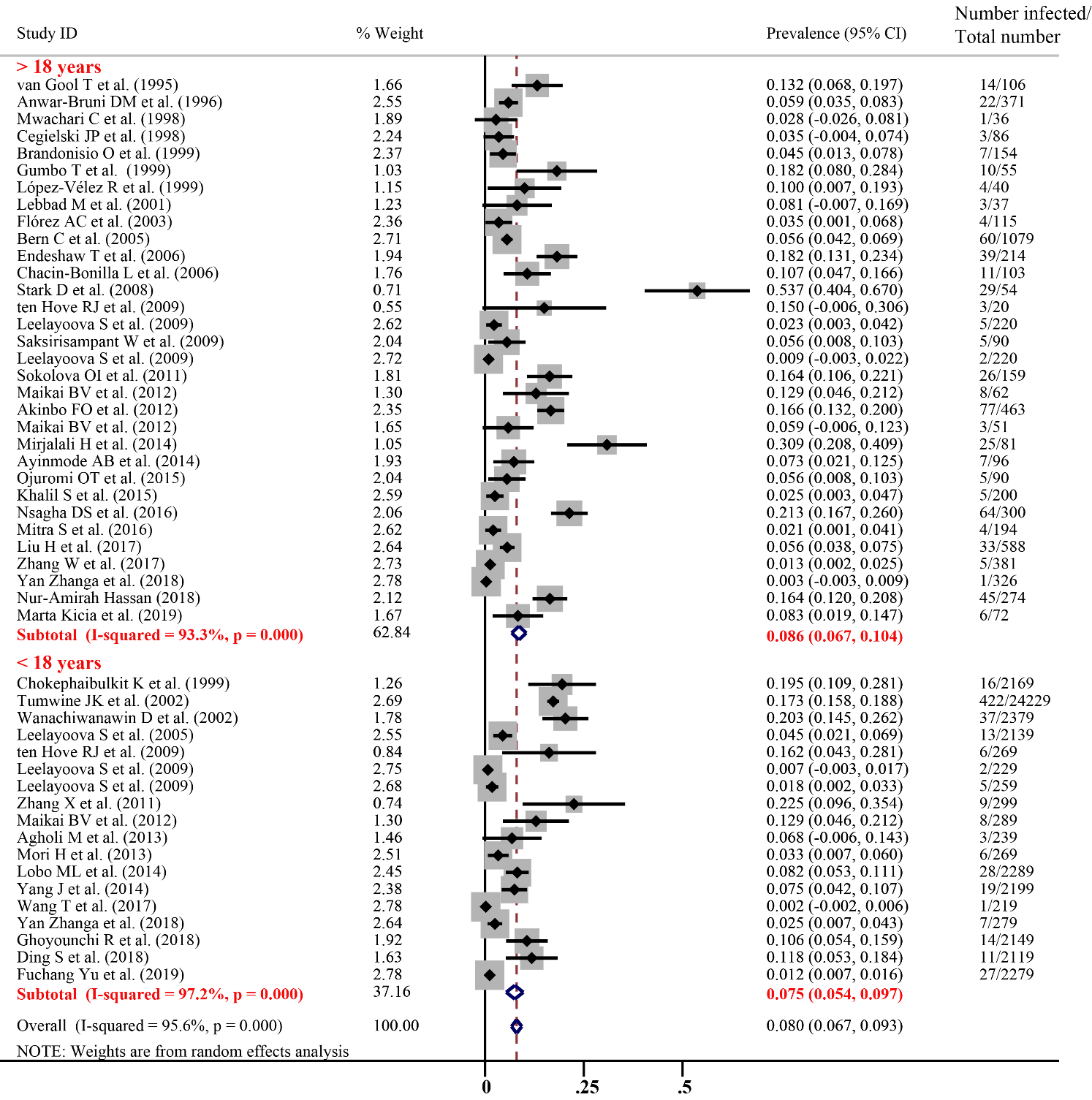


**Figure S3. Forest plot diagram showing microsporidian infection in humans of different age groups.** The red items indicate microsporidia prevalence rate in individuals >18 years and < 18 years and the 95% confidence interval (CI) in the considered studies based on the random effects model. The midpoint of each line shows the estimation of the prevalence and the length of the line indicates the 95% CI of each study. The rhombic sign shows the combinational prevalence rate in corresponding studies.


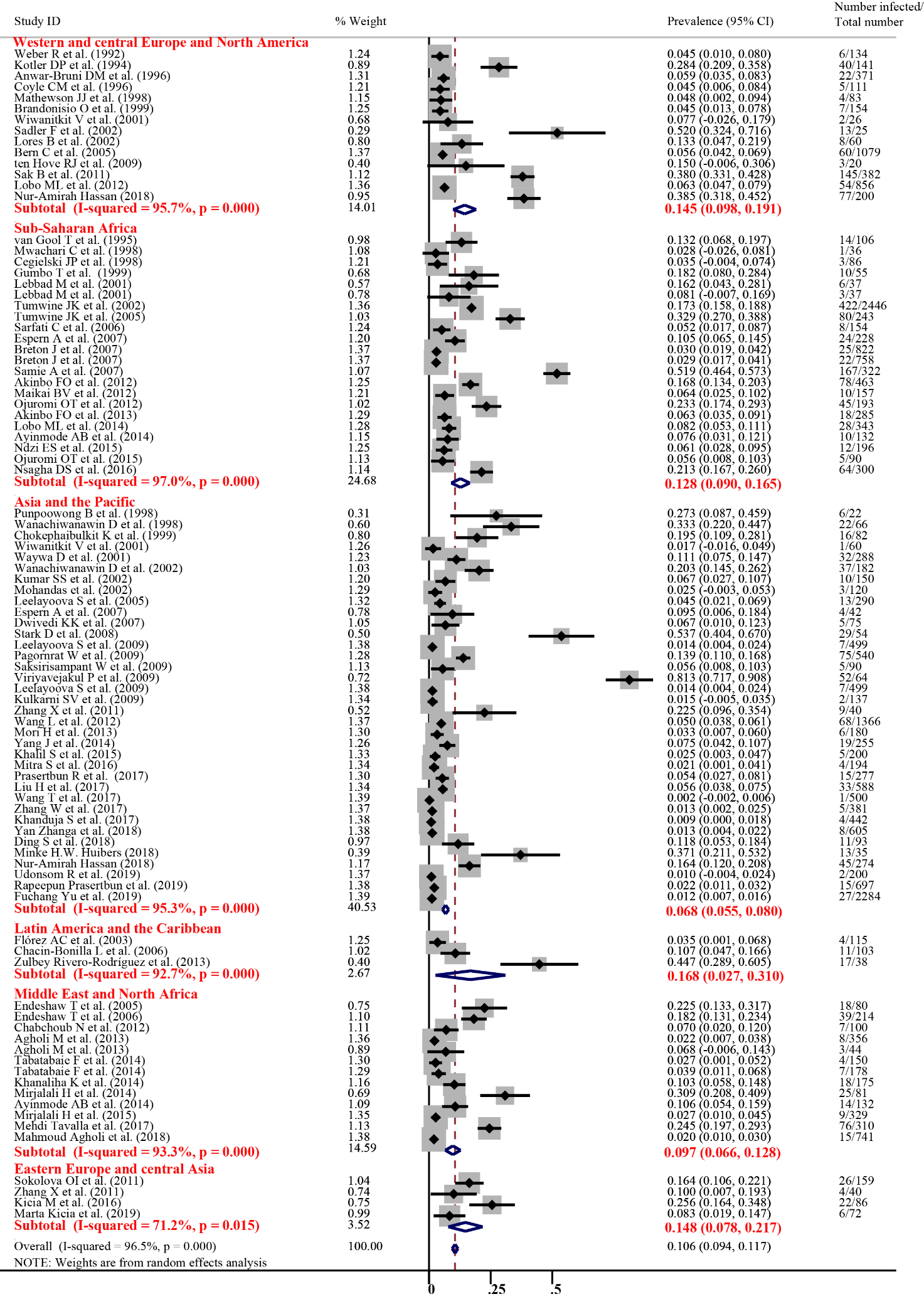


**Figure S4. Forest plot diagram showing microsporidian infection in humans of different regions.** The red items indicate microsporidia prevalence rate in Western and central Europe and North America, Sub-Saharan Africa, Asia and the Pacific, Latin America and the Caribbean, Middle East and North Africa, Eastern Europe and central Asia and the 95% confidence interval (CI) in the considered studies based on the random effects model. The midpoint of each line shows the estimation of the prevalence and the length of the line indicates the 95% CI of each study. The rhombic sign shows the combinational prevalence rate in corresponding studies.


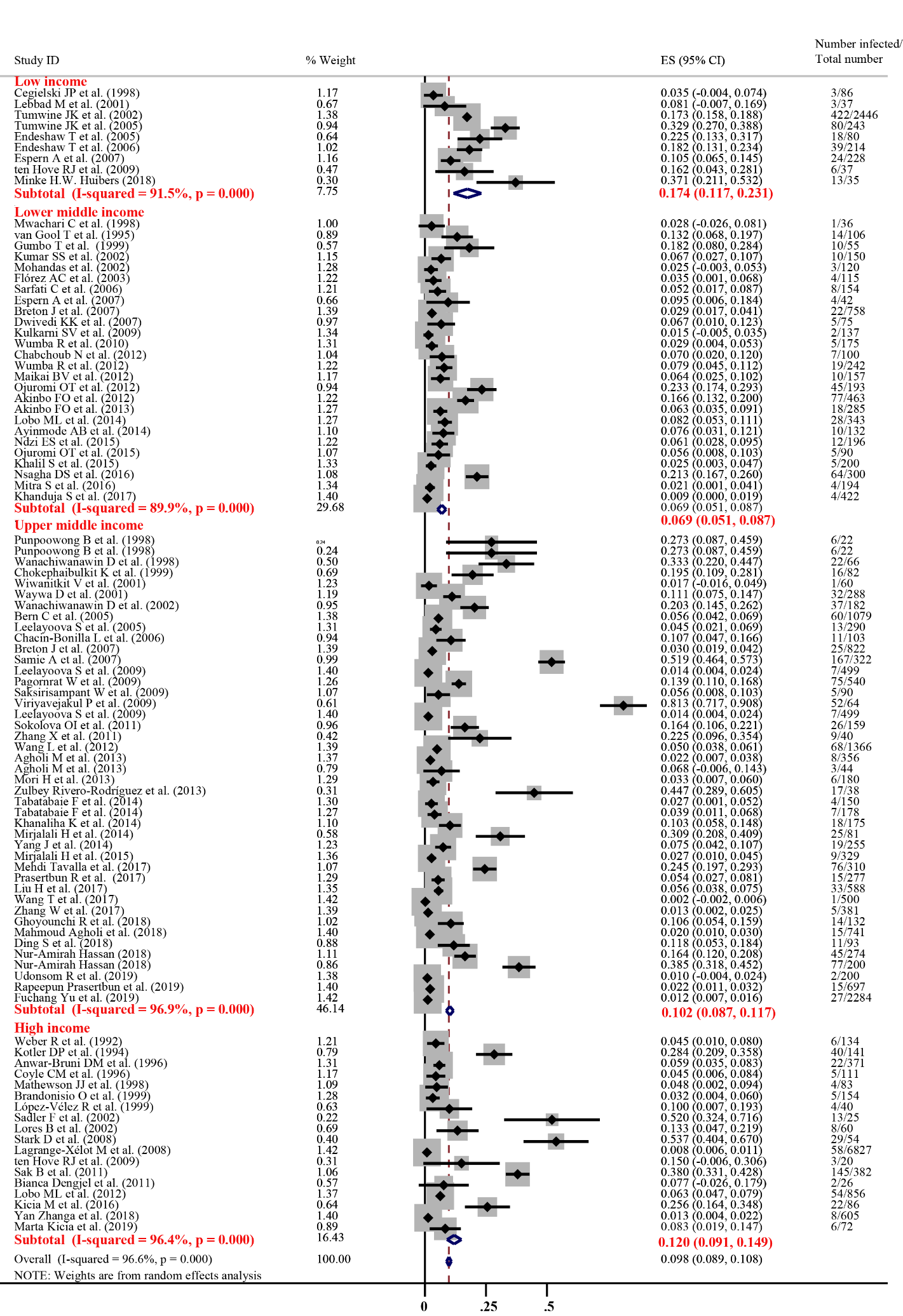


**Figure S5. Forest plot diagram showing microsporidian infection in humans of different income levels.** The red items indicate microsporidia prevalence rate in low income, lower middle income, upper middle income and high income and the 95% confidence interval (CI) in the considered studies based on the random effects model. The midpoint of each line shows the estimation of the prevalence and the length of the line indicates the 95% CI of each study. The rhombic sign shows the combinational prevalence rate in corresponding studies.


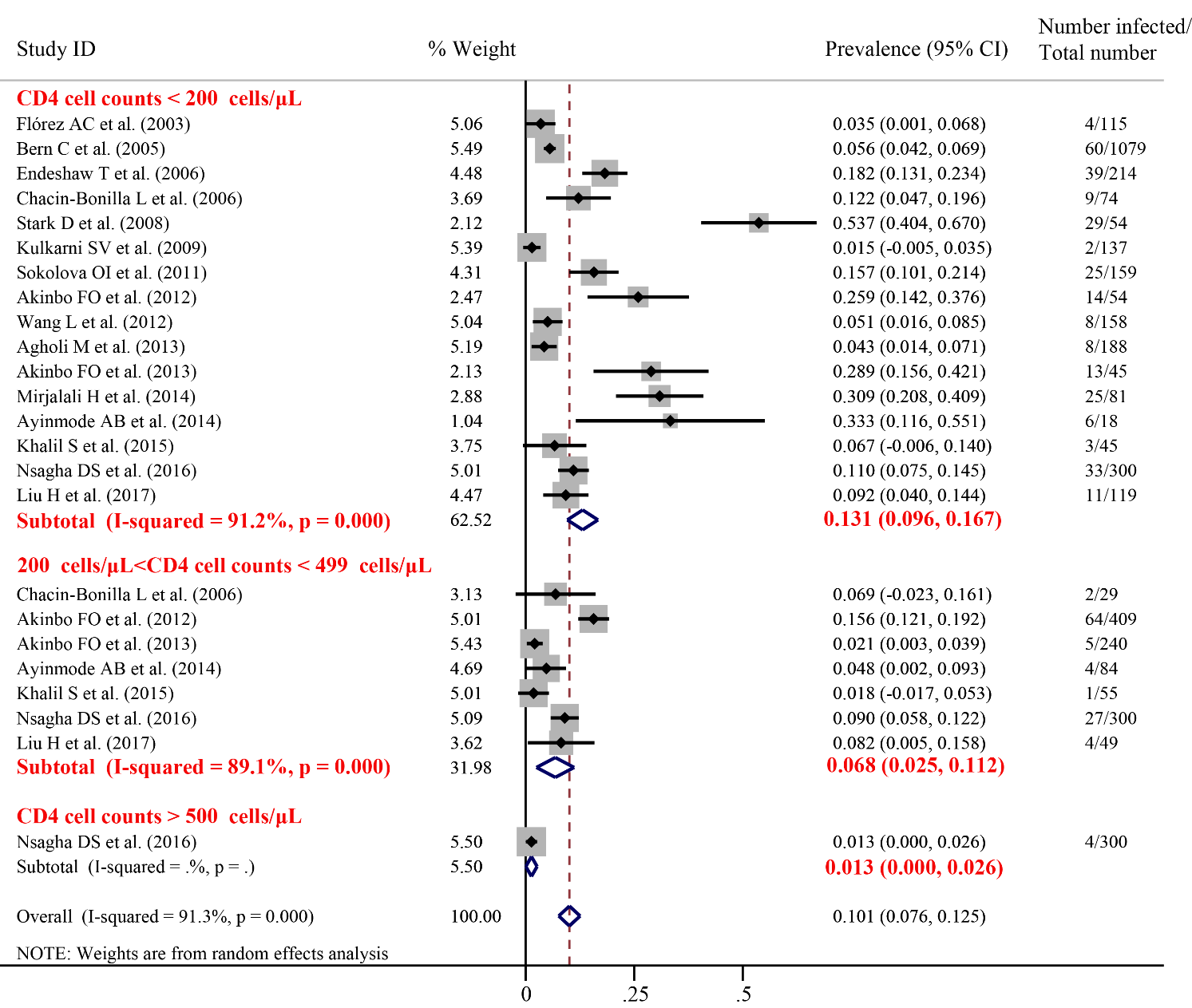


**Figure S6. Forest plot diagram showing microsporidian infection in humans with different CD4 cell counts.** The red items indicate microsporidia prevalence rate in individuals with CD4 cell counts less than 200 cell/μL, individuals with CD4 cell counts over 200 cell/μL but no more than 499 cell/μL, and individuals with CD4 cell counts over 500cell/μL, and the 95% confidence interval (CI) in the considered studies based on the random effects model. The midpoint of each line shows the estimation of the prevalence and the length of the line indicates the 95% CI of each study. The rhombic sign shows the combinational prevalence rate in corresponding studies.


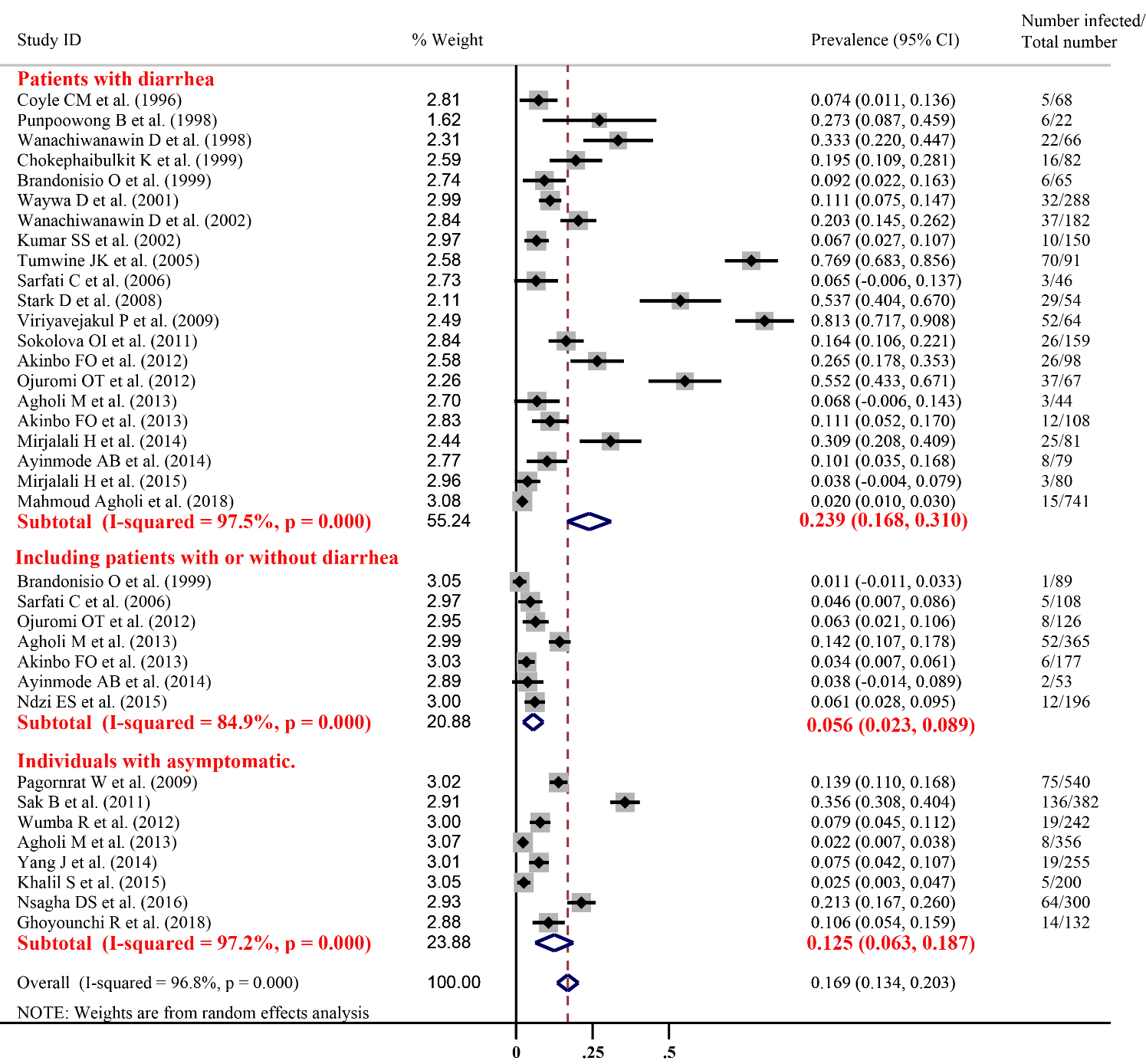


**Figure S7. Forest plot diagram showing microsporidian infection in humans with diarrhea.** The red items indicate microsporidia prevalence rate in patients with diarrhea, include patients with or without diarrhea, and individuals with asymptomatic and the 95% confidence interval (CI) in the considered studies based on the random effects model. The midpoint of each line shows the estimation of the prevalence and the length of the line indicates the 95% CI of each study. The rhombic sign shows the combinational prevalence rate in corresponding studies.


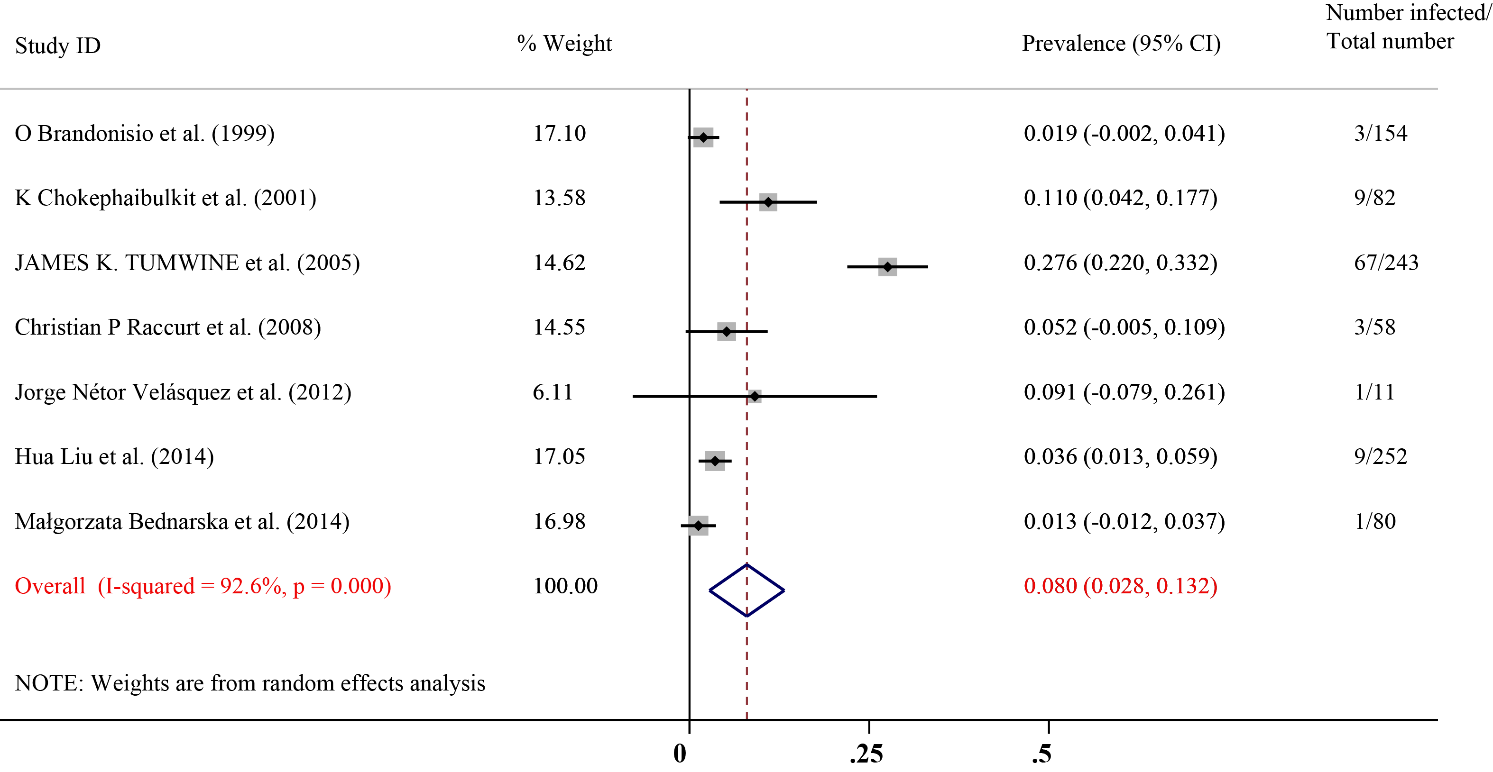


**Figure S8. Forest plot diagram showing the coinfection prevalence rate of microsporidia and *Cryptosporidium* in humans.** The red items indicate coinfection rate and the 95% confidence interval (CI) in the considered studies based on the random effects model. The midpoint of each line shows the estimation of the prevalence and the length of the line indicates the 95% CI of each study. The rhombic sign shows the combinational prevalence rate in corresponding studies.


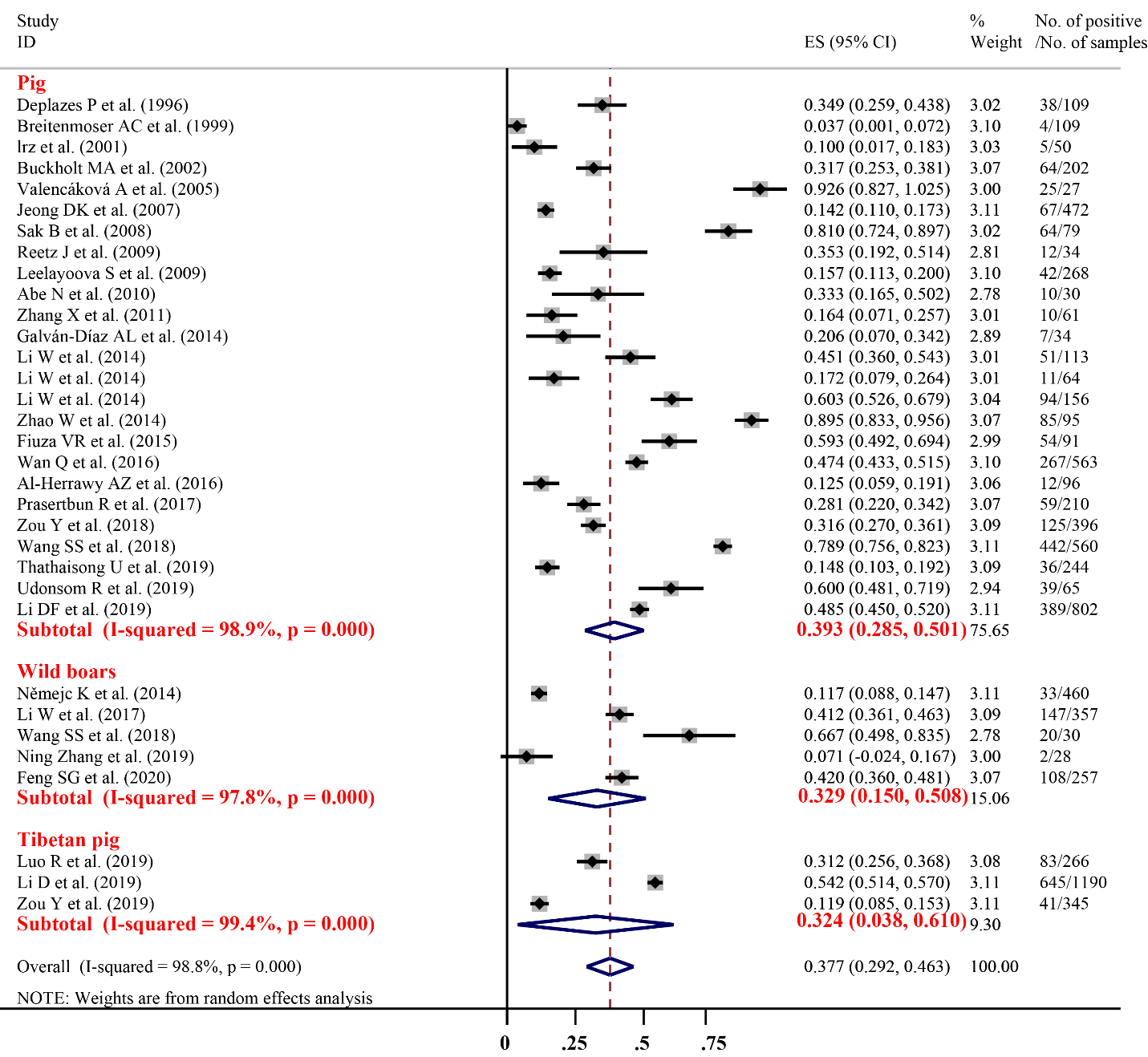


**Figure S9. Forest plot diagram showing the prevalence of microsporidian infection in different species groups of swine.** These red items indicate microsporidia prevalence rate in domestic pig, wild boar and Tibetan pig and the 95% confidence interval (CI) in the considered studies based on the random effects model. The midpoint of each line shows the estimation of the prevalence and the length of the line indicates the 95% CI of each study. The rhombic sign shows the combinational prevalence rate in corresponding studies.


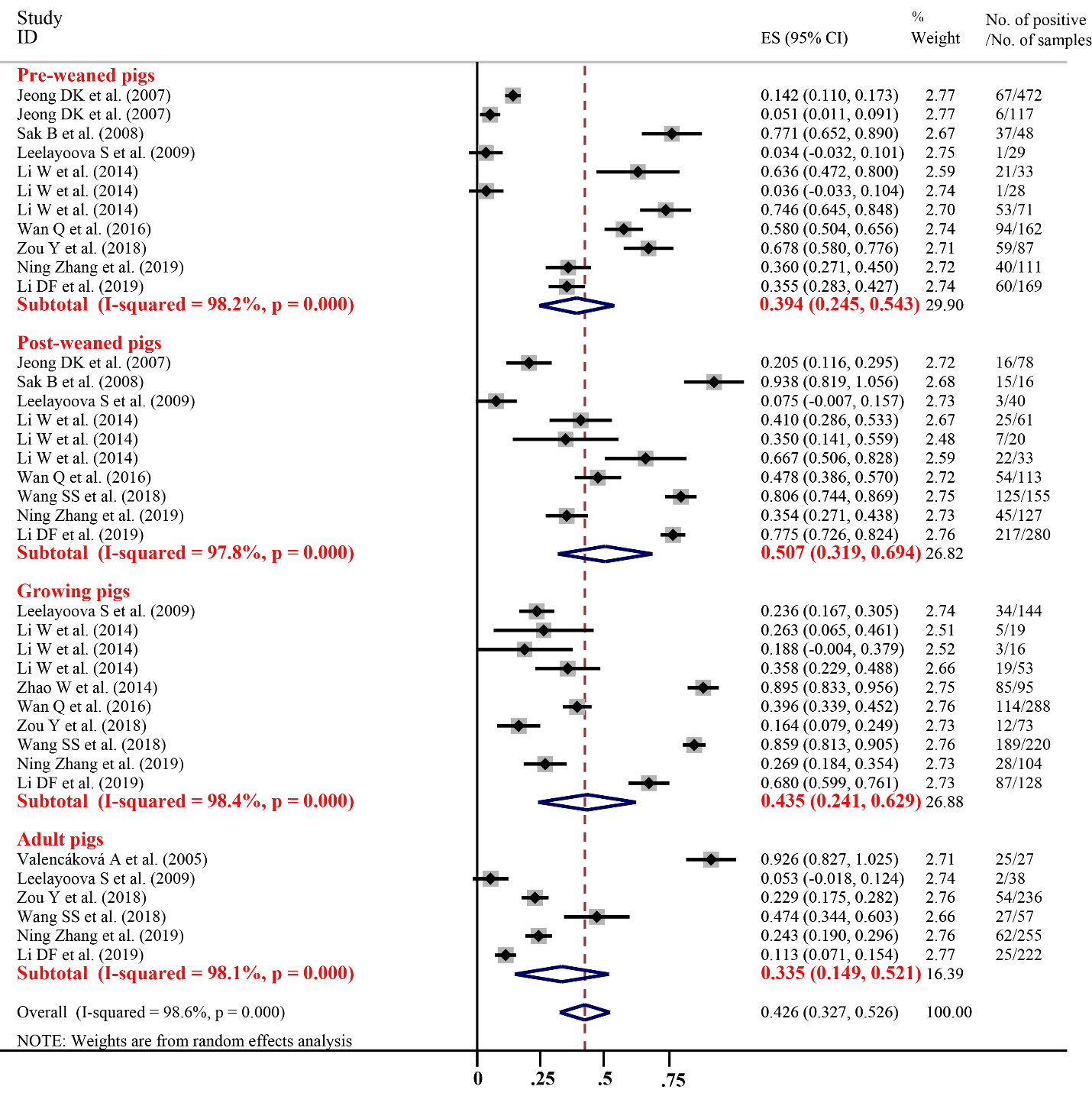


**Figure S10. Forest plot diagram showing the prevalence of microsporidian infection in different age groups of swine.** These red items indicate microsporidia prevalence in pre-weaned pig, post-weaned pig, growing pigs and adult pigs and the 95% confidence interval (CI) in the considered studies based on the random effects model. The midpoint of each line shows the estimation of the prevalence and the length of the line indicates the 95% CI of each study. The rhombic sign shows the combinational prevalence rate in corresponding studies.


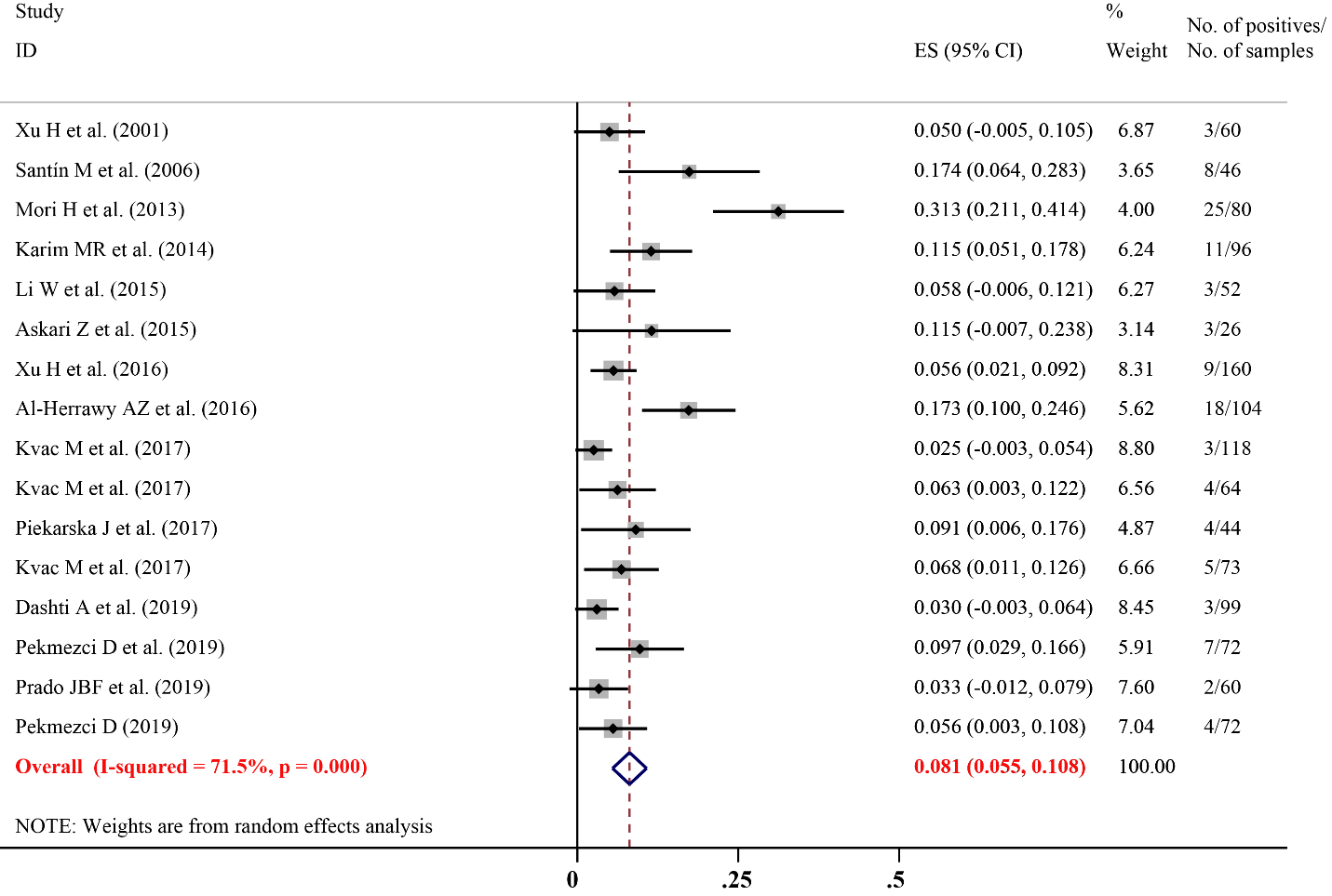


**Figure S11. Forest plot diagram showing the prevalence of microsporidian infection in cats.** The red item indicates microsporidia prevalence in cats and the 95% confidence interval (CI) in the considered studies based on the random effects model. The midpoint of each line shows the estimation of the prevalence and the length of the line indicates the 95% CI of each study. The rhombic sign shows the combinational prevalence rate in corresponding studies.


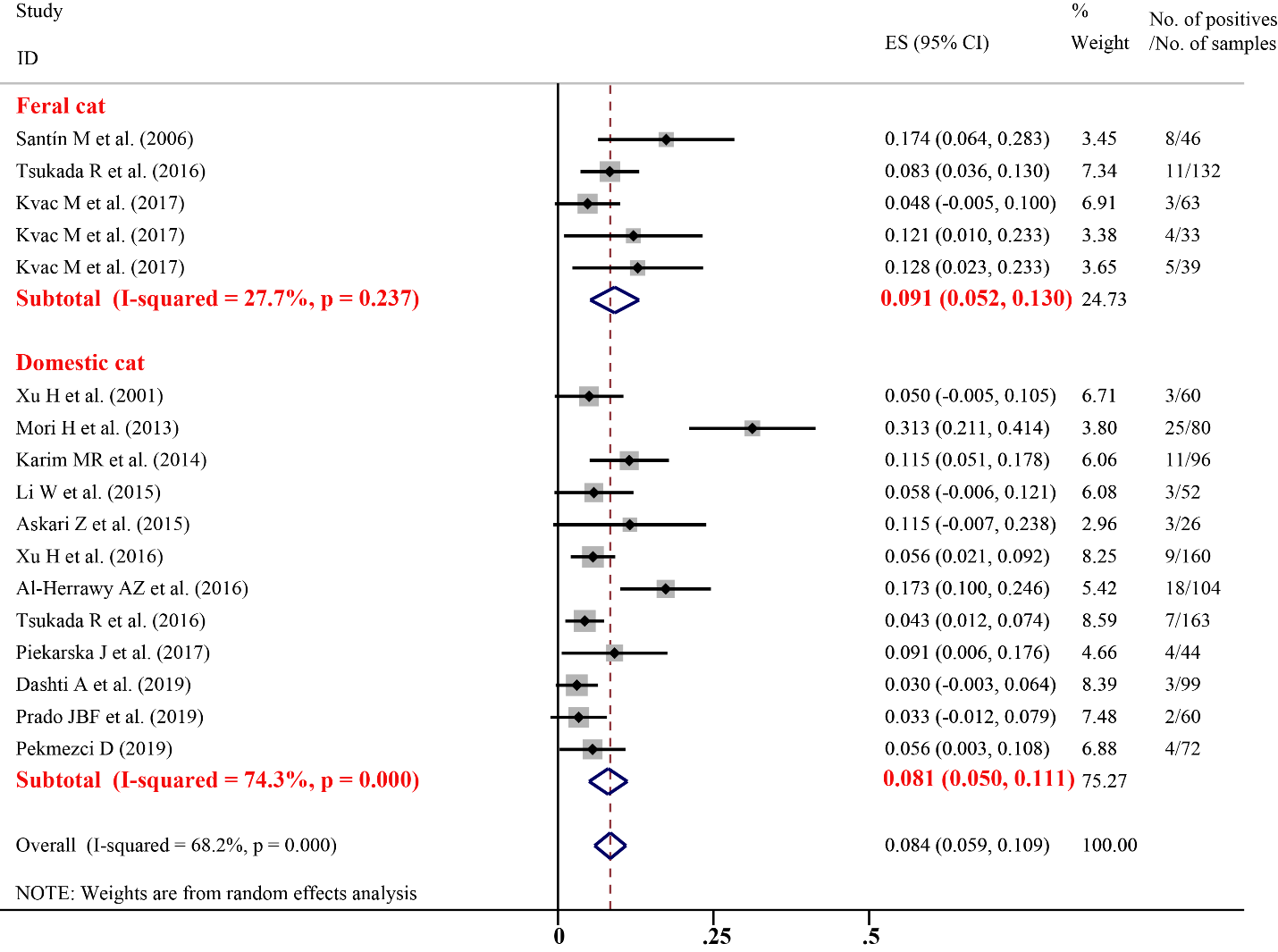


**Figure S12. Forest plot diagram showing the prevalence of microsporidian infection in pet and feral cats.** The red items indicate microsporidia prevalence in Fear cats and domestic cats and the 95% confidence interval (CI) in the considered studies based on the random effects model. The midpoint of each line shows the estimation of the prevalence and the length of the line indicates the 95% CI of each study. The rhombic sign shows the combinational prevalence rate in corresponding studies.


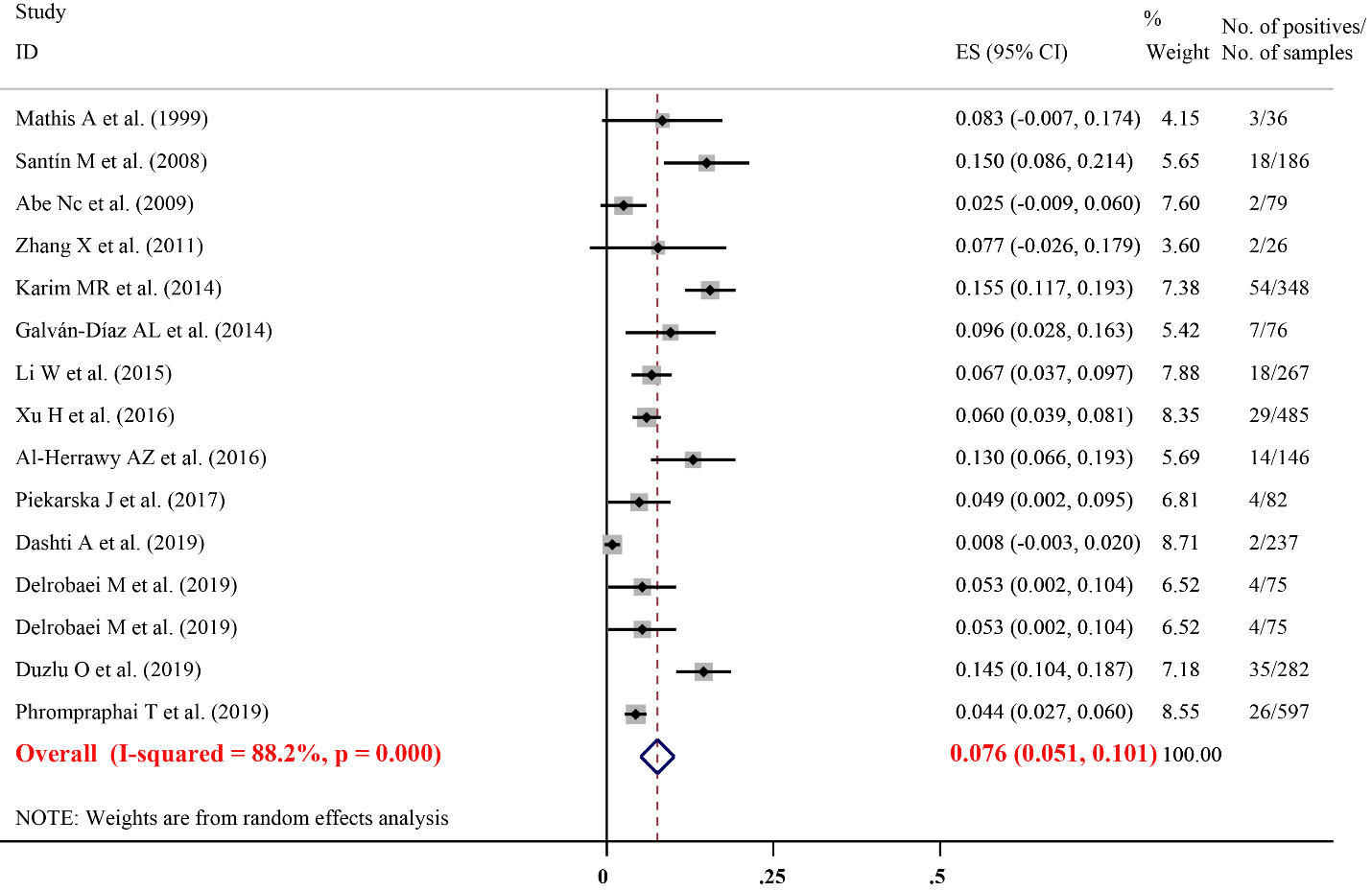


**Figure S13. Forest plot diagram showing the prevalence of microsporidian infection in dogs.** The red item indicates microsporidia prevalence in dogs and the 95% confidence interval (CI) in the considered studies based on the random effects model. The midpoint of each line shows the estimation of the prevalence and the length of the line indicates the 95% CI of each study. The rhombic sign shows the combinational prevalence rate in corresponding studies.


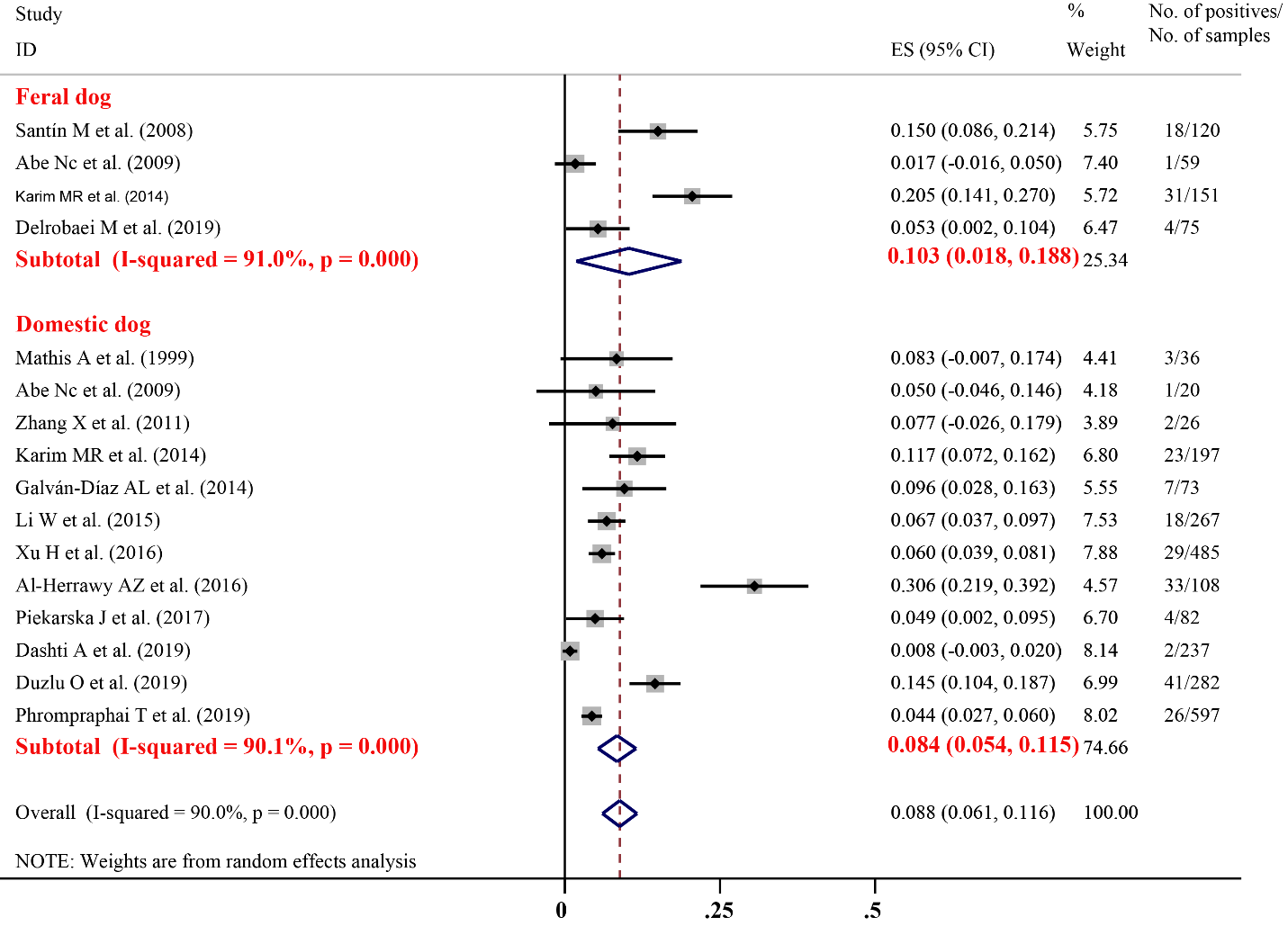


**Figure S14. Forest plot diagram showing the prevalence of microsporidian infection in pet and feral dogs.** The red items indicate microsporidia prevalence in feral dogs and domestic dogs and the 95% confidence interval (CI) in the considered studies based on the random effects model. The midpoint of each line shows the estimation of the prevalence and the length of the line indicates the 95% CI of each study. The rhombic sign shows the combinational prevalence rate in corresponding studies.


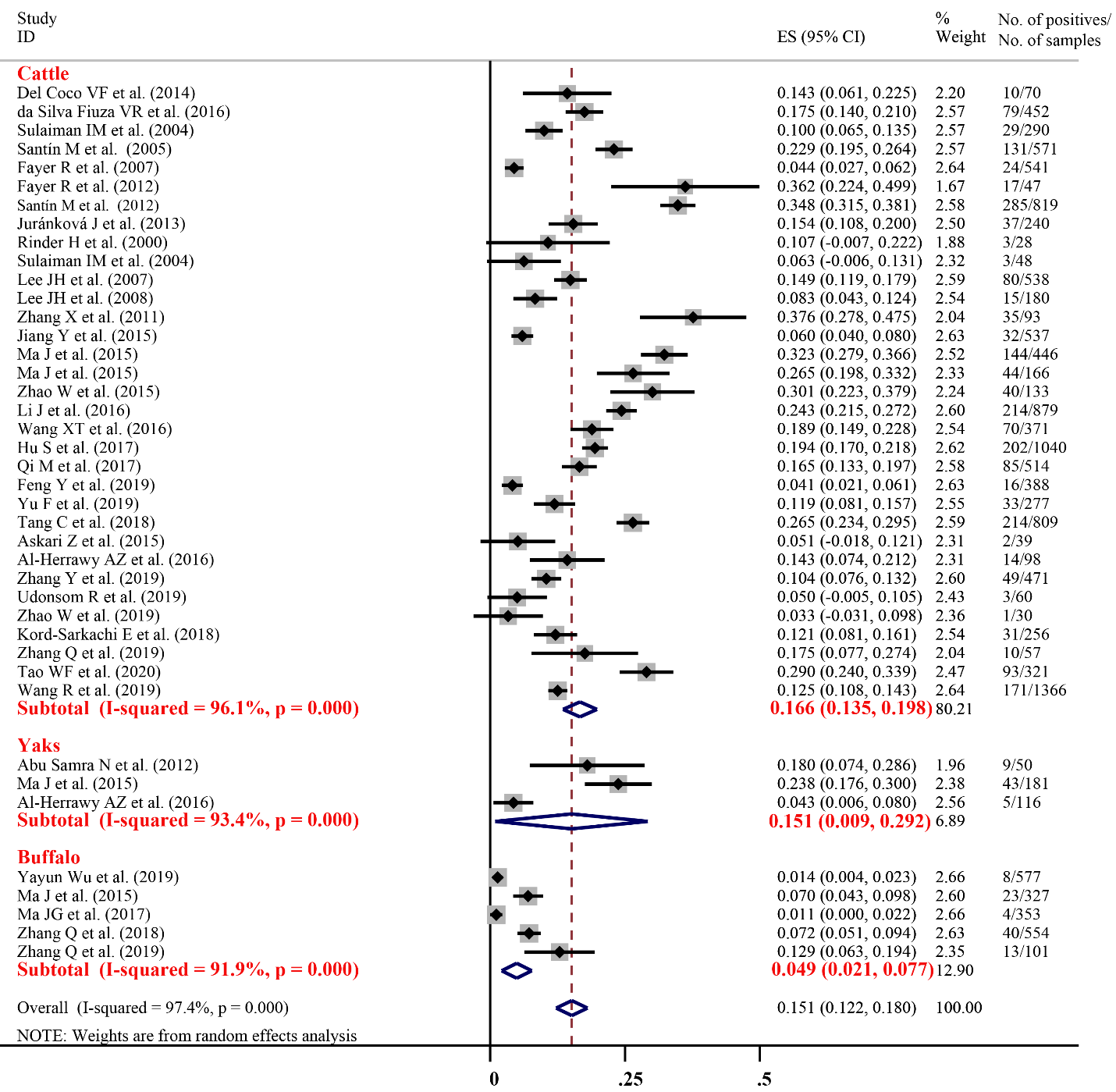


**Figure S15. Forest plot diagram showing the prevalence of microsporidian infection in Bovine.** The red items indicate microsporidia prevalence in cattle, yak and buffalo and the 95% confidence interval (CI) in the considered studies based on the random effects model. The midpoint of each line shows the estimation of the prevalence and the length of the line indicates the 95% CI of each study. The rhombic sign shows the combinational prevalence rate in corresponding studies.


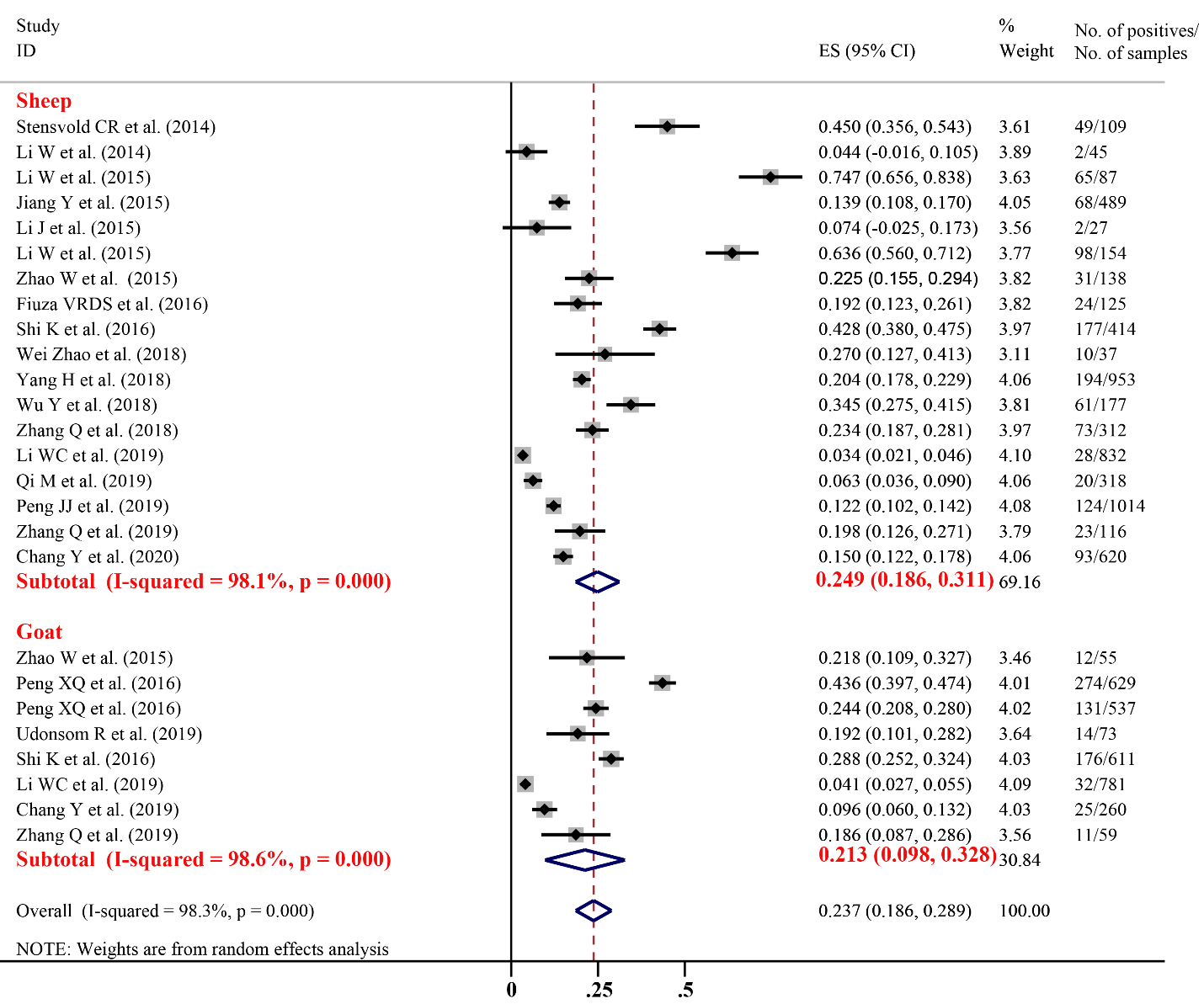


**Figure S16. Forest plot diagram showing the prevalence of microsporidian infection in *Ovis*.** The red items indicate microsporidia prevalence in sheep and goat and the 95% confidence interval (CI) in the considered studies based on the random effects model. The midpoint of each line shows the estimation of the prevalence and the length of the line indicates the 95% CI of each study. The rhombic sign shows the combinational prevalence rate in corresponding studies.


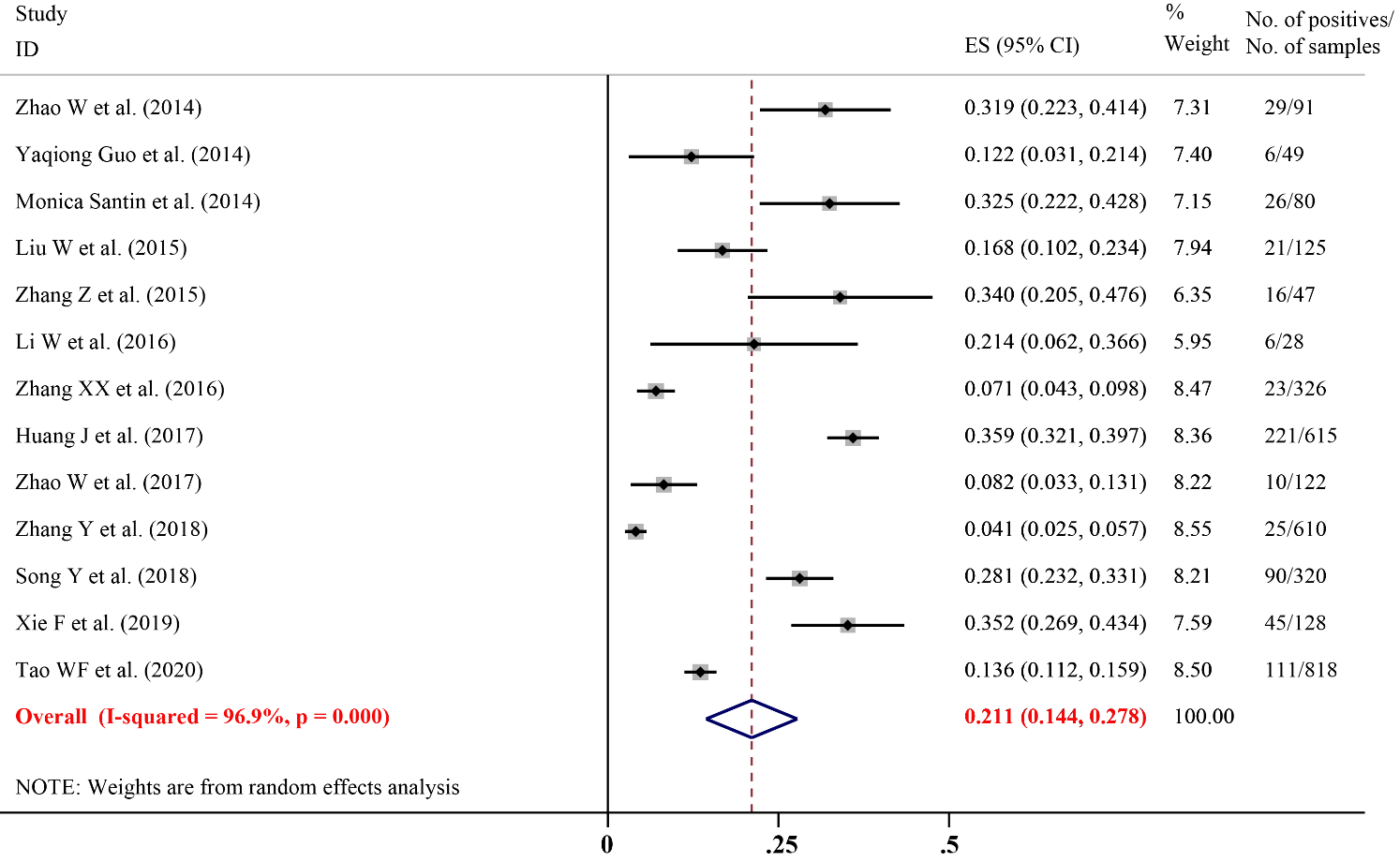


**Figure S17. Forest plot diagram showing the prevalence of microsporidia infection in deer.** The red item indicates microsporidia prevalence in deer and the 95% confidence interval (CI) in the considered studies based on the random effects model. The midpoint of each line shows the estimation of the prevalence and the length of the line indicates the 95% CI of each study. The rhombic sign shows the combinational prevalence rate in corresponding studies.


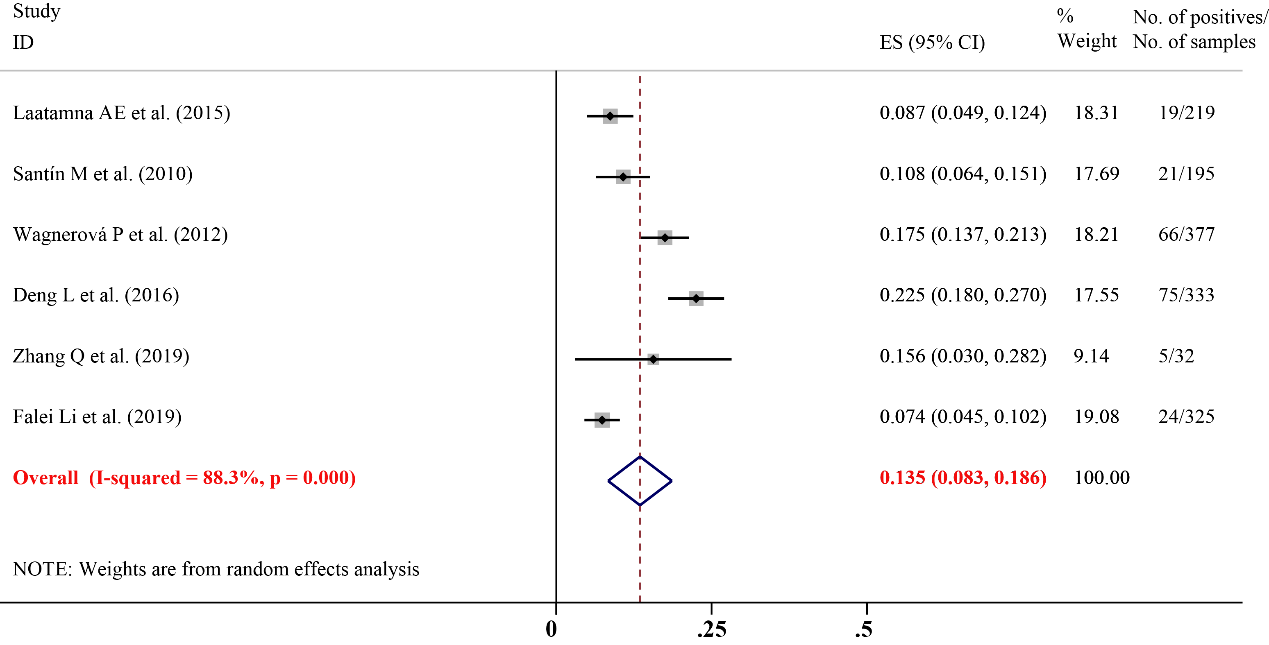


**Figure S18. Forest plot diagram showing the prevalence of microsporidian infection in horses.** The red item indicates microsporidia prevalence in horse and the 95% confidence interval (CI) in the considered studies based on the random effects model. The midpoint of each line shows the estimation of the prevalence and the length of the line indicates the 95% CI of each study. The rhombic sign shows the combinational prevalence rate in corresponding studies.


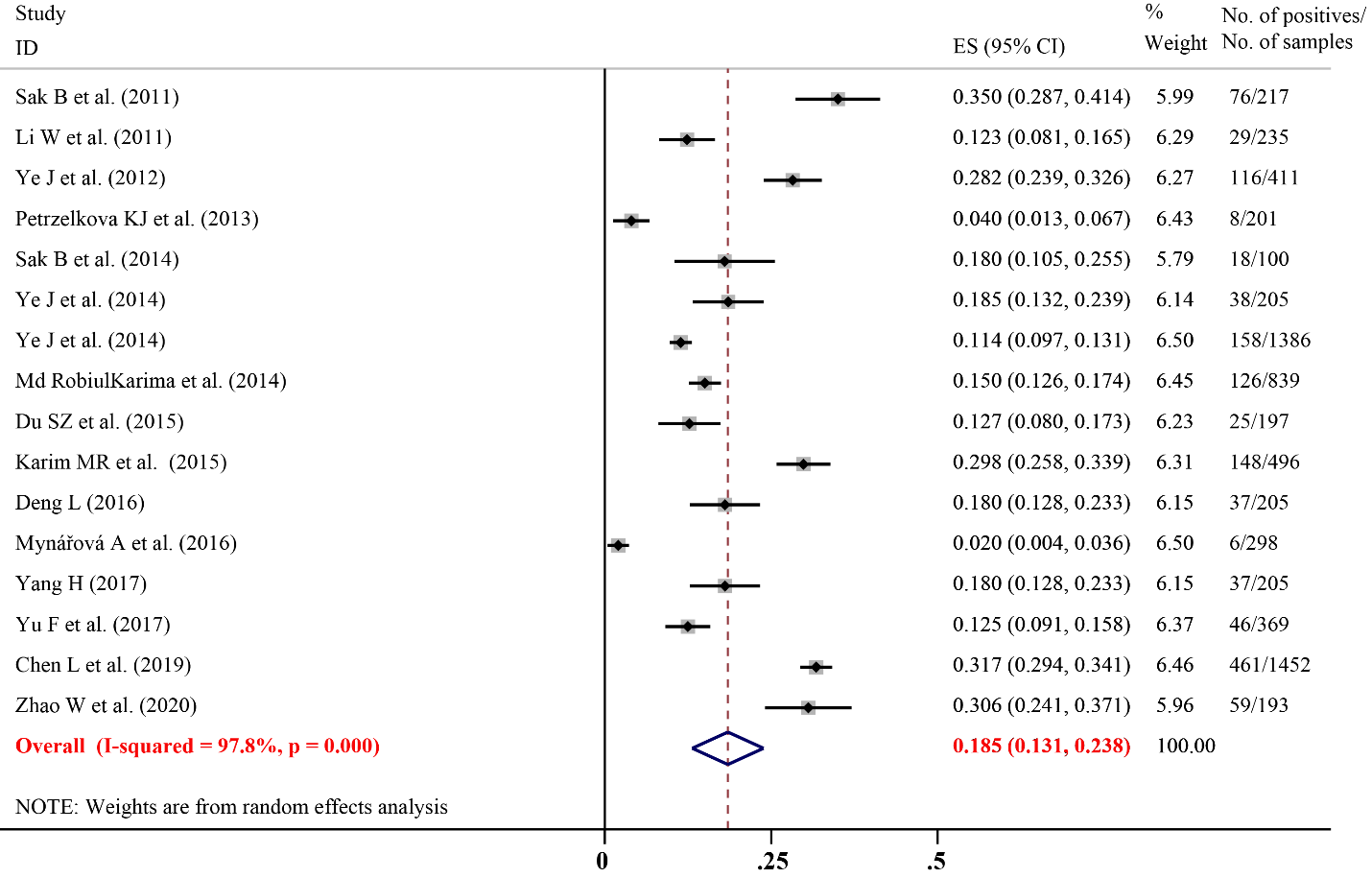


**Figure S19. Forest plot diagram showing the prevalence of microsporidian infection in nonhuman primates.** The red item indicates microsporidia prevalence in no-human primates and the 95% confidence interval (CI) in the considered studies based on the random effects model. The midpoint of each line shows the estimation of the prevalence and the length of the line indicates the 95% CI of each study. The rhombic sign shows the combinational prevalence rate in corresponding studies.


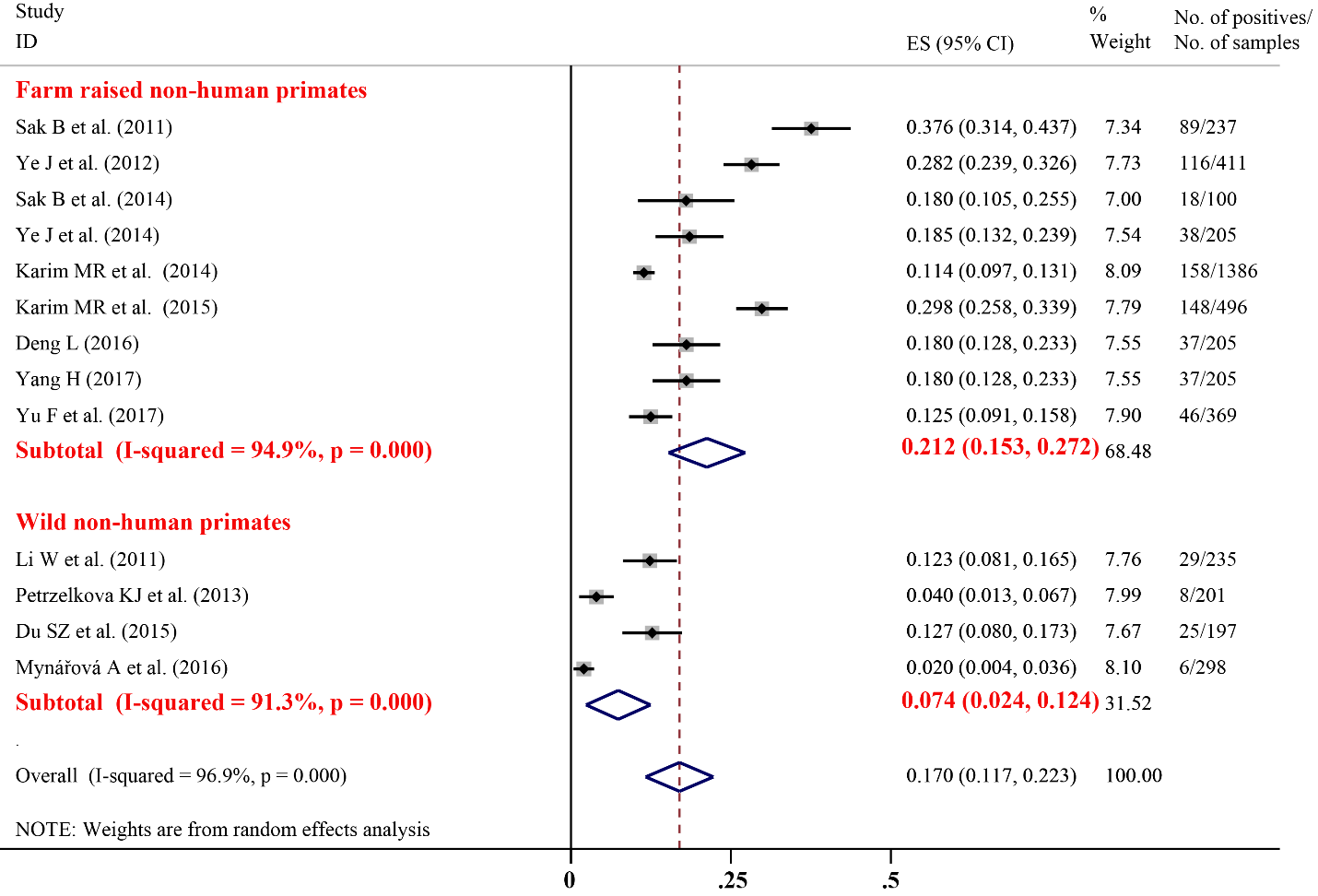


**Figure S20. Forest plot diagram showing the prevalence of microsporidian infection in farm raised and wild no-human primates.** The red items indicate microsporidia prevalence in farm raises no-human primates and wild non-human primates and the 95% confidence interval (CI) in the considered studies based on the random effects model. The midpoint of each line shows the estimation of the prevalence and the length of the line indicates the 95% CI of each study. The rhombic sign shows the combinational prevalence rate in corresponding studies.


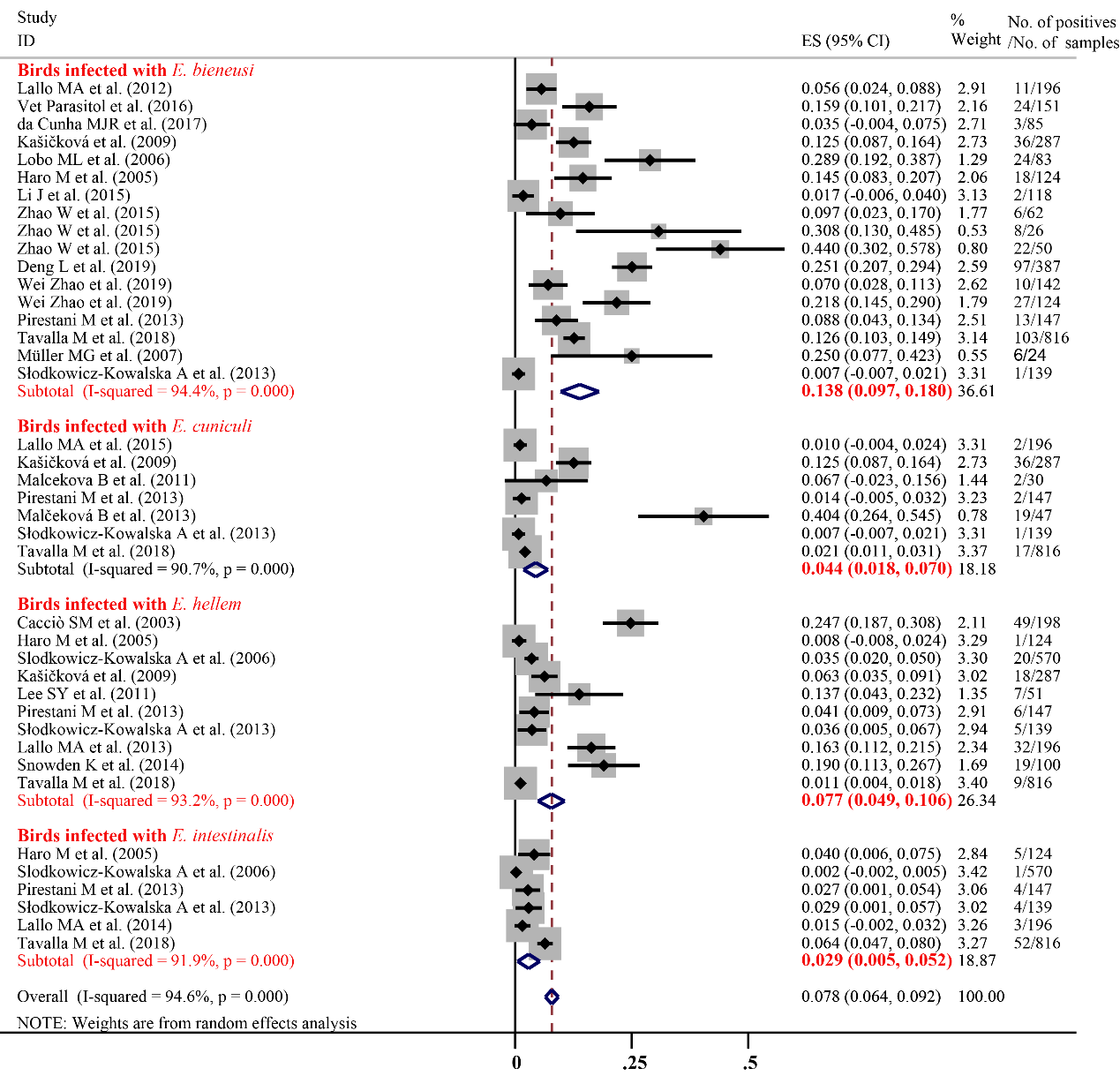


**Figure S21. Forest plot diagram showing the prevalence of microsporidian infection in birds.** The red items indicate *E. bieneusi*, *E. cuniculi*, *E. hellem* and *E. intestinalis* prevalence in birds and the 95% confidence interval (CI) in the considered studies based on the random effects model. The midpoint of each line shows the estimation of the prevalence and the length of the line indicates the 95% CI of each study. The rhombic sign shows the combinational prevalence rate in corresponding studies.


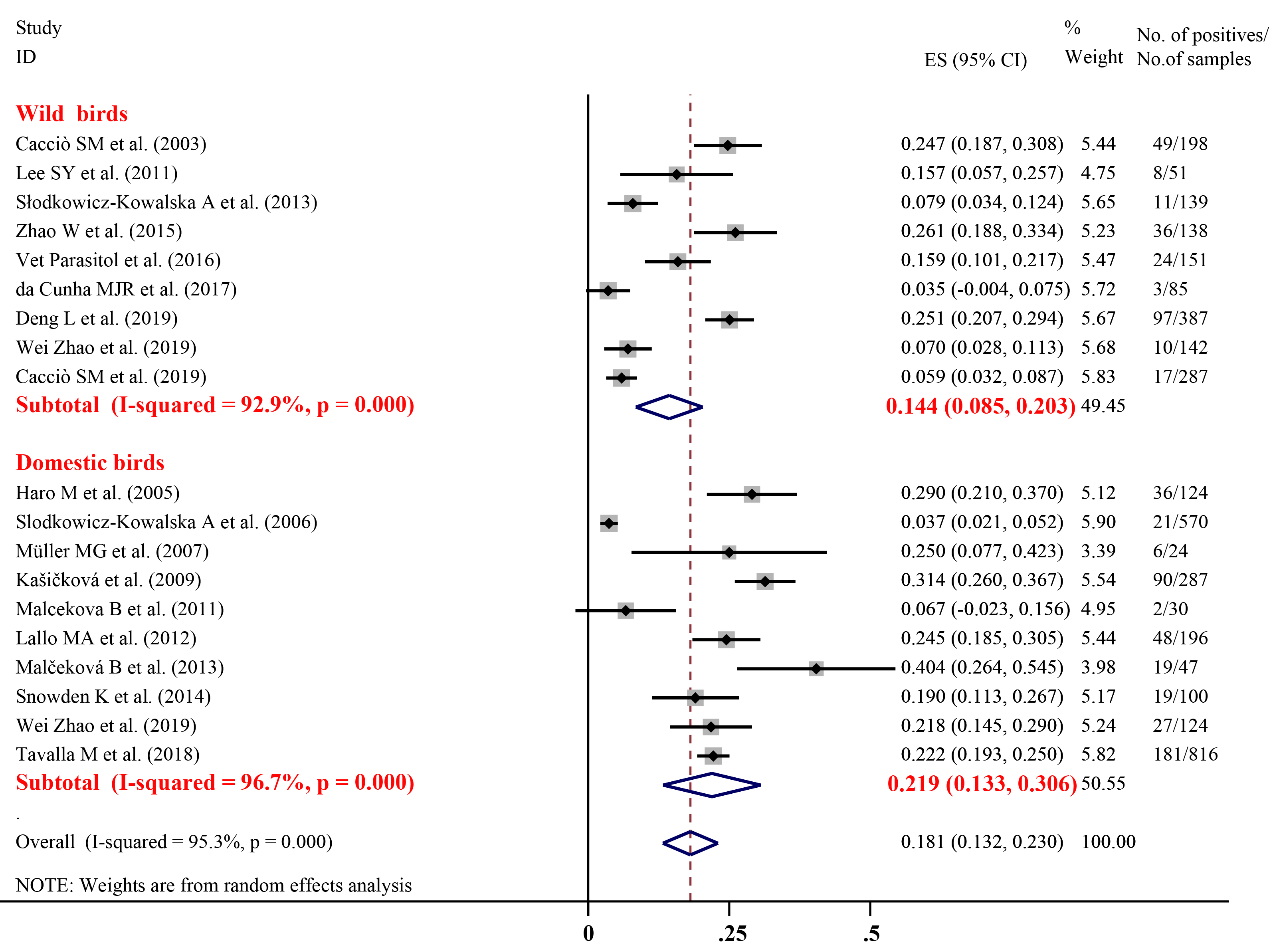


**Figure S22. Forest plot diagram showing the prevalence of microsporidian infection in wild and domestic birds.** The red items indicate microsporidia prevalence in wild birds and domestic birds and the 95% confidence interval (CI) in the considered studies based on the random effects model. The midpoint of each line shows the estimation of the prevalence and the length of the line indicates the 95% CI of each study. The rhombic sign shows the combinational prevalence rate in corresponding studies.


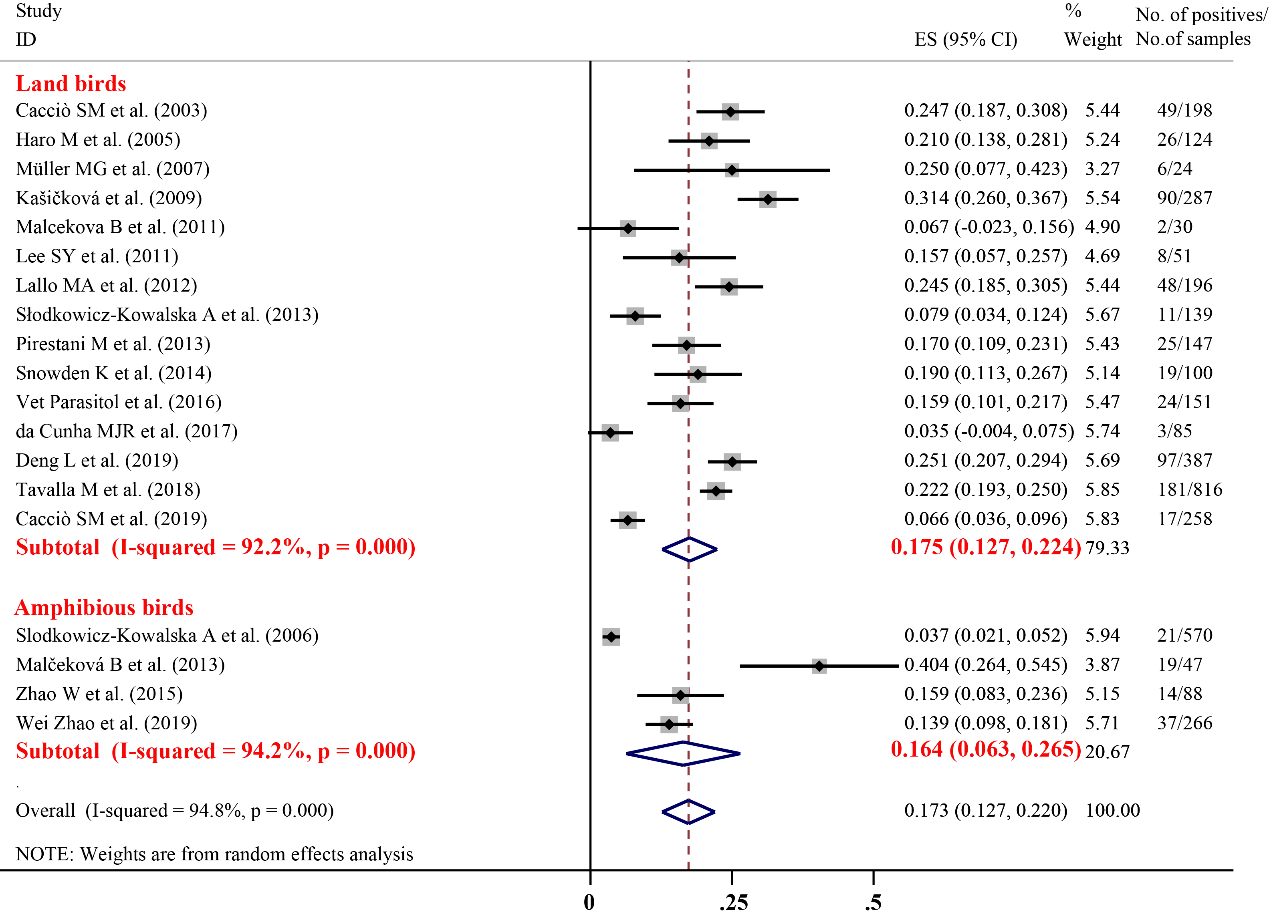


**Figure S23. Forest plot diagram showing the prevalence of microsporidian infection in land and amphibious birds.** The red items indicate microsporidia prevalence in land birds and amphibious birds and the 95% confidence interval (CI) in the considered studies based on the random effects model. The midpoint of each line shows the estimation of the prevalence and the length of the line indicates the 95% CI of each study. The rhombic sign shows the combinational prevalence rate in corresponding studies.


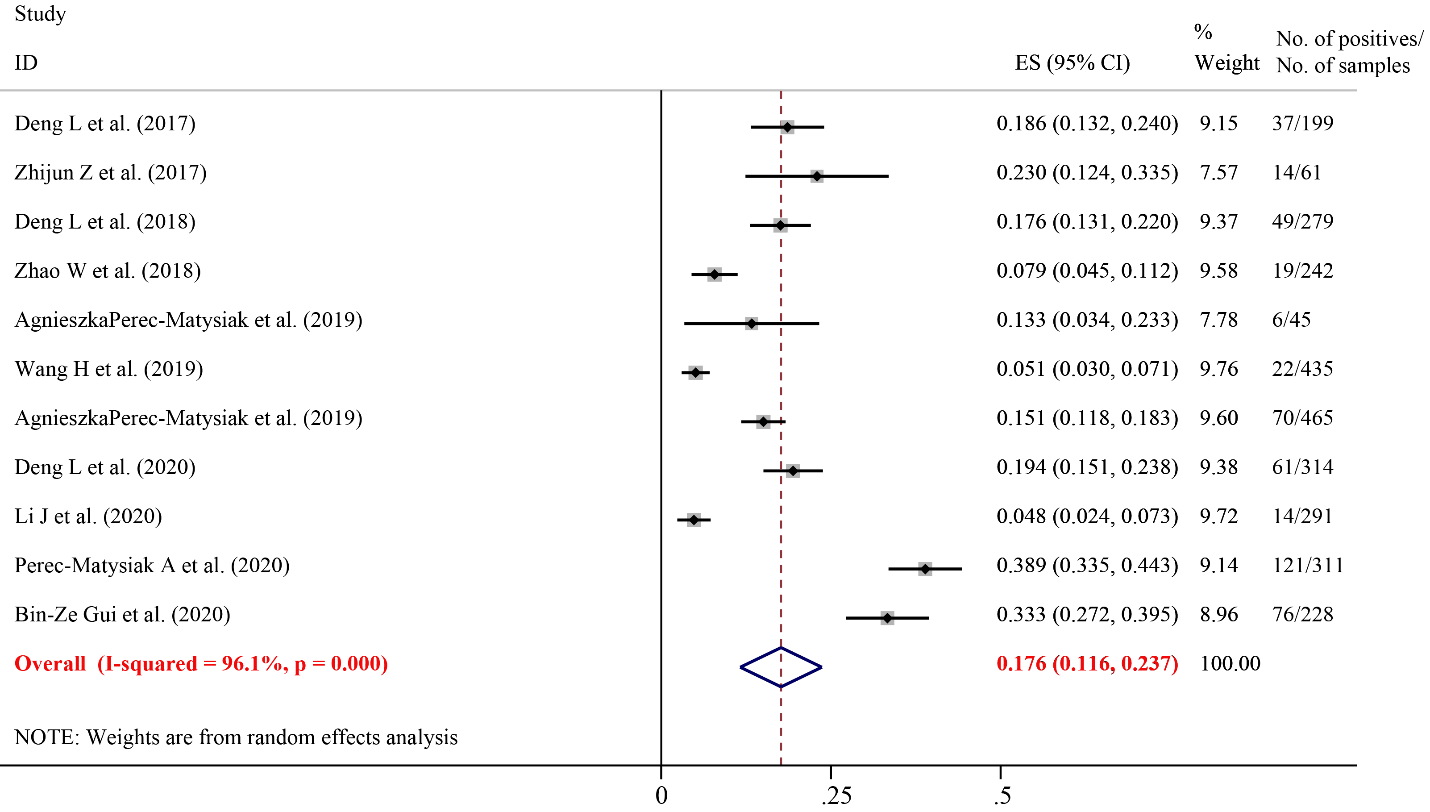


**Figure S24. Forest plot diagram showing the prevalence of microsporidian infection in rodents.** The red item indicates microsporidia prevalence in rodents and the 95% confidence interval (CI) in the considered studies based on the random effects model. The midpoint of each line shows the estimation of the prevalence and the length of the line indicates the 95% CI of each study. The rhombic sign shows the combinational prevalence rate in corresponding studies.


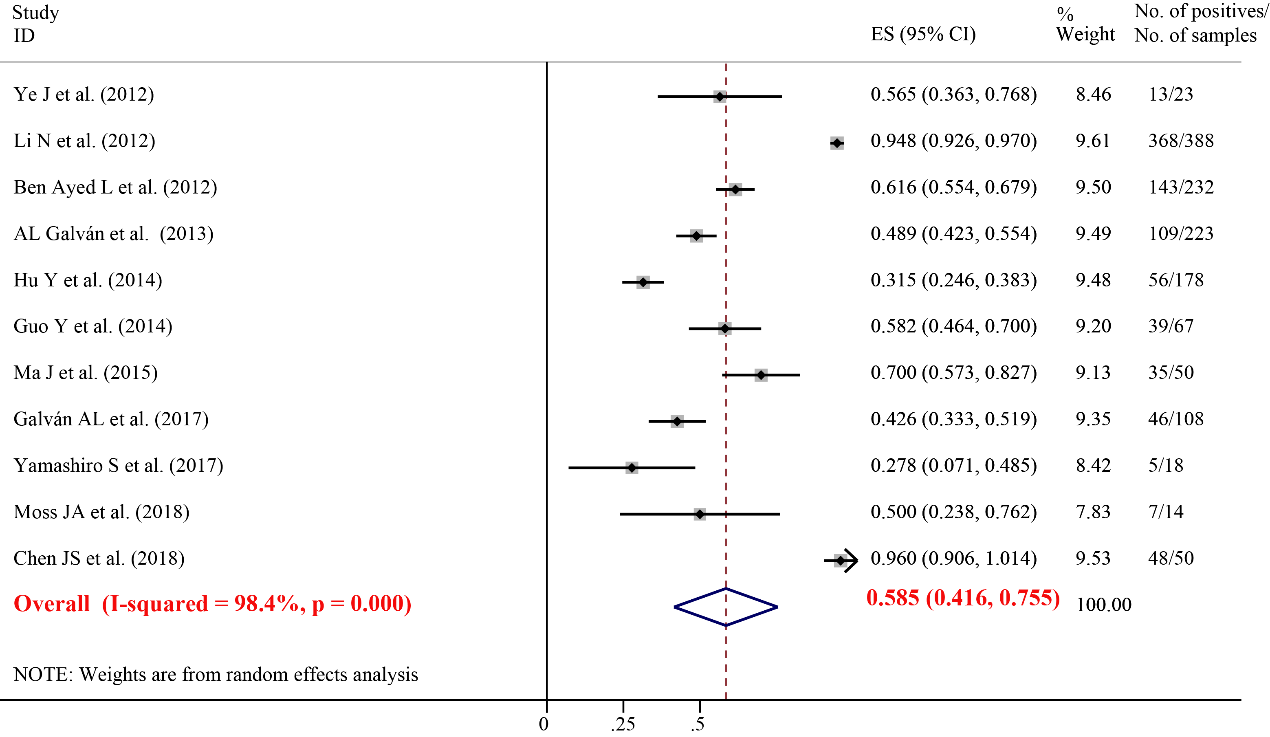


**Figure S25. Forest plot diagram showing the prevalence of microsporidian infection in water.** The red item indicates microsporidia prevalence in water and the 95% confidence interval (CI) in the considered studies based on the random effects model. The midpoint of each line shows the estimation of the prevalence and the length of the line indicates the 95% CI of each study. The rhombic sign shows the combinational prevalence rate in corresponding studies.


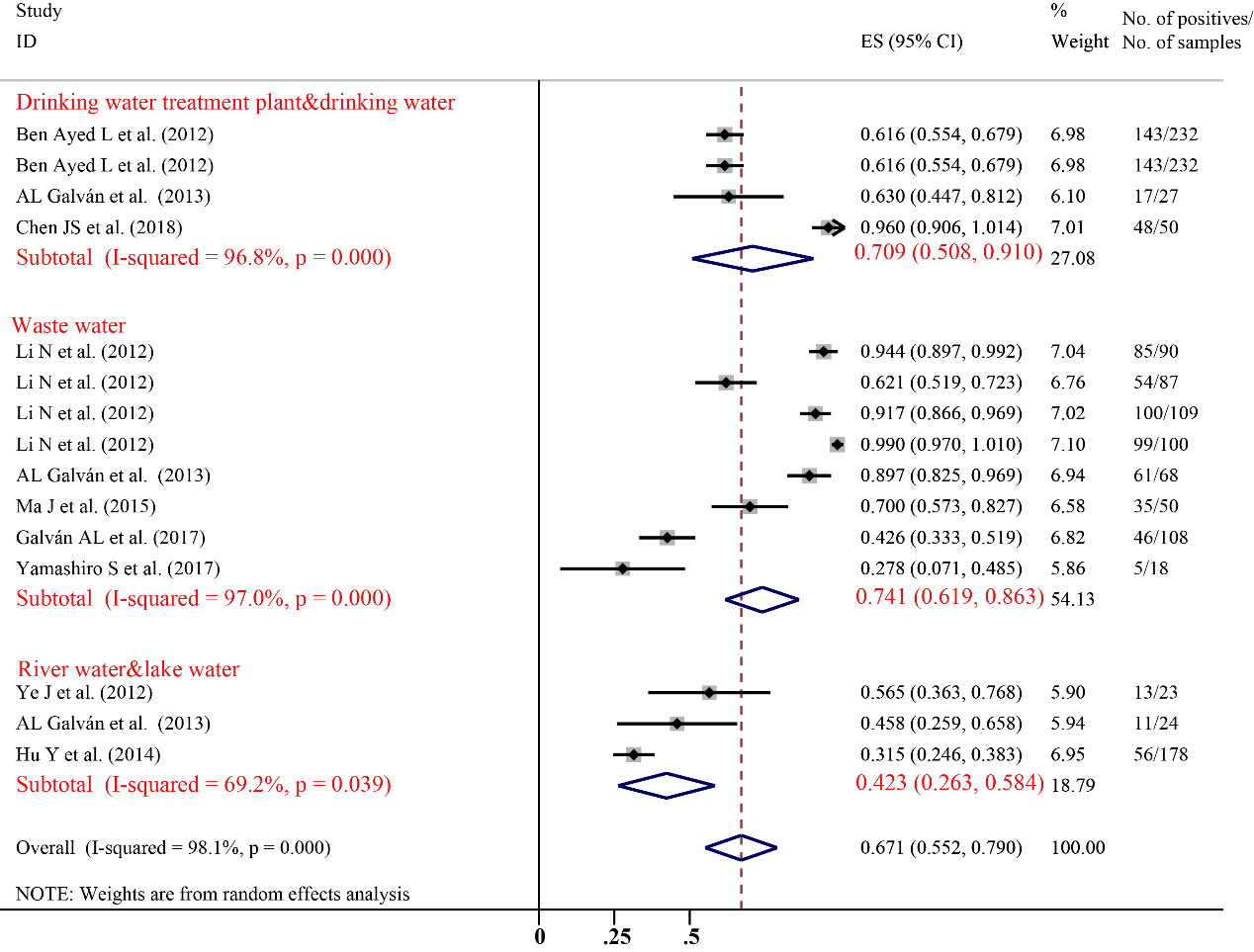


**Figure S26. Forest plot diagram showing the prevalence of microsporidian infection in different water sources.** The red items indicate microsporidia prevalence in different sources of water, and the 95% confidence interval (CI) in the considered studies based on the random effects model. The midpoint of each line shows the estimation of the prevalence and the length of the line indicates the 95% CI of each study. The rhombic sign shows the combinational prevalence rate in corresponding studies.

## Additional Tables

**Table S1.** **Included studies of microsporidian infection in humans**

| **Country** | **Region^*^** | **Income level** | **Age** | **Stata of diarrhea** | **CD4+ T cell counts (cells/µL)** | **No. of samples** | **No. of male** | **No. of female** | **No. of positive samples** | **No. of Male** | **No. of Female** | **Quality score** | **Sampling date** | **Species** | **Reference** |
| --- | --- | --- | --- | --- | --- | --- | --- | --- | --- | --- | --- | --- | --- | --- | --- |
| HIV-positive patients | | | | | | | | | | | | | | | |
| USA | 1 | H | - | - | <350 | 134 | - | - | 6 | - | - | 2 | - | Microsporidia | [1] |
| USA | 1 | H | - | - | - | 141 | - | - | 40 | - | - | 2 | 1996/1-1998/1 | Microsporidia | [2] |
| Zimbabwe | 2 | LM | adult | yes | - | 106 | - | - | 14 | - | - | 3 | 1992/10-1993/1 | *E. bieneusi* | [3] |
| USA | 1 | H | adult | - | - | 371 | - | - | 22 | - | - | 1 | - | Microsporidia | [4] |
| USA | 1 | H | - | yes | - | 68 | - | - | 5 | - | - | 3 | - | *E. bieneusi* | [5] |
|  |  |  | - | no | - | 43 | - | - | 0 | - | - |  |  |  |  |
| Kenya | 2 | LM | 18-56 | yes | - | 36 | 21 | 15 | 1 | 0 | 1 | 2 | 1992/4-1992/7 | *E. bieneusi* | [6] |
| Tanzania | 2 | L | adult | yes | - | 86 | - | - | 3 | - | - | 3 | - | *Enterocytozoan* | [7] |
| Thailand | 3 | UM | - | yes | - | 22 | - | - | 6 | - | - | 3 | - | *Enterocytozoan* | [8] |
| Thailand | 3 | UM | - | yes | - | 66 | - | - | 22 | - | - | 3 | 1995-1996 | Microsporidia | [9] |
| Thailand | 3 | UM | children | yes | - | 82 | - | - | 16 | - | - | 4 | 2008/4-2009/2 | Microsporidia | [10] |
| Zimbabwe | 2 | LM | adult | - | - | 55 | - | - | 10 | - | - | 3 | 1995/7-1995/12 | *E. bieneusi* | [11] |
| Italy | 1 | H | adult | yes | - | 65 | - | - | 6 | - | - | 2 | 1993/12-1998/2 | Microsporidia | [12] |
|  |  |  |  | no | - | 89 | - | - | 1 | - | - |  |  |  |  |
| Guinea-Bissau | 2 | L | 23-68 | yes | - | 37 | - | - | 3 | - | - | 3 | 1993/8-1994/1 | Microsporidia | [13] |
| Thailand | 3 | UM | - | - | - | 60 | - | - | 1 | - | - | 4 | 2000/3-2000/4 | Microsporidia | [14] |
| Thailand | 3 | UM | - | yes | - | 288 | - | - | 32 | - | - | 3 | 1999/11- 2000/8 | Microsporidia | [15] |
| United Kingdom | 1 | H | - | unknow | - | 25 | - | - | 13 | - | - | 2 | - | *E. bieneusi* | [16] |
| Thailand | 3 | UM | children | yes | - | 95 | - | - | 24 | - | - | 3 | - | Microsporidia | [17] |
| India | 3 | LM | - | yes | - | 150 | - | - | 10 | - | - | 2 | - | Microsporidia | [18] |
| India | 3 | LM | - | - | - | 120 | - | - | 3 | - | - | 3 | - | Microsporidia | [19] |
| Colombia | 4 | LM | 18-71 | - | - | 115 | - | - | 4 | - | - | 3 | 2001 | Microsporidia | [20] |
| Ethiopia | 5 | L | - | yes | - | 80 | - | - | 18 | - | - | 1 | - | Microsporidia | [21] |
| Peru | 4 | UM | 19-73 | unknow | <200 | 1079 | - | - | 60 | - | - | 3 | 2000-2002 | *E. bieneusi* | [22] |
| Uganda | 2 | L | 1-60 | yes | - | 91 | - | - | 70 | - | - | 4 | 2002/11-2003/5 | *E. bieneusi* | [23] |
| Ethiopia | 5 | L | adult | yes | <100 | 214 | - | - | 39 | - | - | 2 | - | Microsporidia | [24] |
| Venezuela | 4 | UM | adult | - | - | 103 | - | - | 11 | - | - | 4 | - | Microsporidia | [25] |
|  |  |  |  |  | <200 | 74 |  |  | 9 |  |  |  |  |  |  |
|  |  |  |  |  | >200 | 29 |  |  | 2 |  |  |  |  |  |  |
| Cameroon | 2 | LM | adult | yes | - | 46 | - | - | 3 | - | - | 4 | 2001/1-2003/7 | *E. bieneusi* | [26] |
|  |  |  |  | no |  | 108 | - | - | 5 | - | - |  |  |  |  |
| Niger | 2 | L | 7-50 | - | - | 87 | - | - | 24 | - | - | 3 | - | *E. bieneusi* | [27] |
| Gabon | 2 | UM | 16-75 | - | 1-1164 | 822 | - | - | 25 | - | - | 1 | 2002/9-2003/12 | *E. bieneusi* | [28] |
| Cameroon | 2 | LM | 1-85 | - | - | 758 | - | - | 22 | - | - | 1 | 2003/3 | *E. bieneusi* | [29] |
| Vietnam | 3 | LM | 7-50 | - | - | 42 | - | - | 3 | - | - | 3 | 2004/11-2005/8 | *E. bieneusi* | [30] |
|  |  |  |  |  |  |  | - | - | 1 | - | - | 3 |  | *E. intestinalis* |  |
| India | 3 | LM | - | - | - | 75 | - | - | 5 | - | - | 2 | - | *Microsporidia* | [31] |
| Australia | 3 | H | adults | yes | <200 | 54 | 54 | 0 | 29 | 29 | 0 | 2 | - | *E. bieneusi* | [32] |
| France | 1 | H | 17-87 | - | 0-431 | 6827 | 5122 | 1705 | 58 | 56 | 2 | 2 | 1995/5-2003/9 | *E. bieneusi* | [33] |
| Malawi | 2 | L | children | - | - | 37 | 21 | 16 | 6 | - | - | 2 | 2003-2004 | *E. bieneusi* | [34] |
| Netherlands | 1 | H | adults | - | - | 20 | 13 | 7 | 3 | - | - | 2 | 2003-2004 | *E. bieneusi* | [34] |
| Thailand |  | UM | - | mix | - | 77 | - | - | 14 | - | - | 3 | - | *E. bieneusi* | [35] |
| Thailand | 3 | UM | 20-66 | - | - | 90 | 73 | 17 | 5 | - | - | 3 | 2005 | *E. bieneusi* | [36] |
| Thailand | 3 | UM | - | yes | - | 64 | - | - | 52 | - | - | 3 | - | Microsporidia | [37] |
| India | 3 | LM | - | - | <200 | 137 | - | - | 2 | - | - | 3 | 2002/3-2007/3 | Microsporidia | [38] |
|  |  |  |  |  | 200-499 | - | - | - | 0 | - | - |  |  |  |  |
|  |  |  |  |  | >500 | - | - | - | - | - | - |  |  |  |  |
| Congo | 2 | LM | >16 | yes | - | 87 | - | - | 5 | - | - | 2 | 2009/12-2012/1 | *E. bieneusi* | [39] |
|  |  |  |  | yes | - | 87 | - | - | 1 | - | - |  |  | *E. intestinalis* |  |
|  |  |  |  | no | - | 88 |  |  | 5 |  |  |  |  | *E. bieneusi* |  |
| Russia | 6 | UM | adult | yes | 149 | 159 | - | - | 2 | - | - | 3 | 2006-2009 | *E. bieneusi* | [40] |
|  |  |  |  |  | 42-92 | 159 | - | - | 1 | - | - |  |  | *E. cuniculi* |  |
|  |  |  |  |  | 38 | 159 | - | - | 20 | - | - |  |  | *E. hellem* |  |
|  |  |  |  |  | 4-1213 | 159 | - | - | 1 | - | - |  |  | *E. intestinalis* |  |
|  |  |  |  |  | 49 | 159 | - | - | 1 | - | - |  |  | *E. cuniculi + E. intestinalis* |  |
|  |  |  |  |  | 140-576 | 159 | - | - | 1 | - | - |  |  | *Microsporidium* sp. |  |
| Nigeria | 2 | LM | 21-70 | - | - | 463 | 134 | 329 | 77 | 21 | 56 | 3 | 2009/8-2010/8 | *E. bieneusi* | [41] |
|  |  |  |  | - | <200 | 54 | - | - | 14 | - | - |  |  |  |  |
|  |  |  |  | - | >200 | 409 | - | - | 64 | - | - |  |  |  |  |
|  |  |  |  | yes | - | 98 | - | - | 26 | - | - |  |  |  |  |
|  |  |  |  | no | - | 365 | - | - | 52 | - | - |  |  |  |  |
| Nigeria | 2 | LM | 0-3 | - | - | 62 | - | - | 8 | - | - | 2 | 2008/7-2009/4 | *E. bieneusi* | [42] |
|  |  |  | 18-59 | - | - | 51 | - | - | 3 | - | - |  |  |  |  |
| Nigeria | 2 | LM | - | yes | - | 67 | - | - | 37 | - | - | 3 | - | Microsporidia | [43] |
|  |  |  |  | no | - | 126 | - | - | 8 | - |  |  |  |  |  |
| Congo | 2 | LM | 15-73 | mix | - | 242 | 87 | 155 | 19 | - | - | 2 | - | *E. bieneusi* | [44] |
| Portugal | 1 | H | - | - | - | 561 | 408 | 153 | 39 | - | - | 3 | - | *E. bieneusi* | [45] |
| Tunisia | 5 | LM | 28.9(mean) | - | - | 100 | 40 | 60 | 7 | 2 | 5 | 2 | 2005-2008 | *E. bieneusi* | [46] |
| China | 3 | UM | 4–72 | - | - | 683 | 328 | 344 | 39 | - | - | 3 | 2007/3-2010/11 | *E. bieneusi* | [47] |
|  |  |  |  |  | <200 | 158 | - | - | 8 | - | - |  |  |  |  |
| Venezuela | 4 | UM | - | - | - | 38 | - | - | 17 | - | - | 3 | - | Microsporidia | [48] |
| Iran | 5 | UM | 10–69 | mix | - | 356 | 273 | 83 | 8 | - | - | 1 | 2010/5-2011/5 | *E. bieneusi* | [49] |
|  |  |  |  |  | <200 | 188 | - | - | 8 | - | - |  |  |  |  |
|  |  |  |  |  | 200-500 | 117 | - | - | 0 | - | - |  |  |  |  |
|  |  |  |  |  | >50 | 51 | - | - | 0 | - | - |  |  |  |  |
| Nigeria | 2 | LM | - | yes | - | 108 | - | - | 12 | - | - | 3 | - | *E. bieneusi* | [50] |
|  |  |  | - | no | - | 177 | - | - | 6 | - | - |  |  |  |  |
|  |  |  | - | - | <200 | 45 | - | - | 13 | - | - |  |  |  |  |
|  |  |  | - | - | >200 | 240 | - | - | 5 | - | - |  |  |  |  |
| Nigeria | 2 | LM | - | - | - | 132 | 29 | 73 | 10 | 6 | 4 | 2 | - | *E. bieneusi* | [51] |
|  |  |  | >18 | - | - | 96 | - | - | 7 | - | - |  |  |  |  |
|  |  |  | <18 | - | - | 6 | - | - | 3 | - | - |  |  |  |  |
|  |  |  | - | yes | - | 79 | - | - | 8 | - | - |  |  |  |  |
|  |  |  | - | no | - | 53 | - | - | 2 | - | - |  |  |  |  |
|  |  |  | - | - | <200 | 18 | - | - | 6 | - | - |  |  |  |  |
|  |  |  | - | - | >200 | 84 | - | - | 4 | - | - |  |  |  |  |
| Iran | 5 | UM | 20-65 | yes | <200 | 81 | 58 | 23 | 25 | 17 | 8 | 2 | - | *E. bieneusi* | [52] |
| Nigeria |  | LM | 22–65 | - | - | 90 | - | - | 5 | 5 | 0 | 2 | 2006/10-2007/2 | *E. bieneusi* | [53] |
| India | 3 | LM | 19-59 | mix | - | 200 | 132 | 68 | 5 | - | - | 3 | 2016/3-2017/1 | *E. bieneusi* | [54] |
|  |  |  |  | yes | - | 100 | - | - | 5 | - | - |  |  |  |  |
|  |  |  |  | no | - | 100 | - | - | 0 | - | - |  |  |  |  |
|  |  |  |  | - | <200 | 45 | - | - | 3 | - | - |  |  |  |  |
|  |  |  |  | - | 200-350 | 55 | - | - | 1 | - | - |  |  |  |  |
|  |  |  |  | - | >350 | 100 | - | - | 0 | - | - |  |  |  |  |
| Iran | 5 | UM | - | yes | - | 80 | - | - | 3 | - | - | 2 | - | *E. bieneusi* | [55] |
| Cameroon | 2 | LM | 21-65 | mix | - | 300 | 64 | 236 | 64 | - | - | 3 | - | Microsporidia | [56] |
|  |  |  |  |  | <200 | 300 | -- |  | 33 | - | - |  |  |  |  |
|  |  |  |  |  | 200-499 | 300 | - | - | 27 | - | - |  |  |  |  |
|  |  |  |  |  | >500 | 300 |  | - | 4 | - | - |  |  |  |  |
| India | 3 | LM | >40 | - | - | 194 | - | - | 4 | - | - | 2 | - | Microsporidia | [57] |
| China | 3 | UM | adult | -- | - | 285 | 216 | 69 | 33 | 27 | 6 | 3 | 2013/7-2014/7 | *E. bieneusi* | [58] |
|  |  |  |  |  | <200 | 119 | - | - | 11 | - | - |  |  |  |  |
|  |  |  |  | - | >200 | 49 | - | - | 4 | - | - |  |  |  |  |
| India | 3 | LM |  | - | - | 222 | 169 | 53 | 4 | 4 | 0 | 2 | 2005/1 | Microsporidia | [59] |
|  |  |  |  |  | <200 | 155 | - | - | 4 | - | - |  |  |  |  |
|  |  |  |  |  | 200–500 | 55 | - | - | 0 | - | - |  |  |  |  |
|  |  |  |  |  | >500 | 8 | - | - | 0 | - | - |  |  |  |  |
| Iran | 5 | UM | All range | - | - | 102 | - | - | 21 | - | - | 3 |  | *E. bieneusi* | [60] |
|  |  |  |  | - | - | 102 | - | - | 8 | - | - |  |  | *Encephalitozoon* |  |
| Malaysia | 3 | UM | >18 | - | - | 119 | - | - | 16 | - | - | 3 | 2016/3-2017/3 | *E. bieneusi* | [61] |
| Iran | 5 | UM | 3-94 | yes | - | 741 | - | - | 15 | - | - | 4 | 2009/10- 2014/10 | Microsporidia | [62] |
| **Gastrointestinal disorders** | | | | | | | | | | | | | | | |
| USA | 1 | H | - | yes | - | 83 | - | - | 4 | - | - | 2 | - | Microsporidia | [63] |
| Uganda | 2 | L | 0-6months | yes | - | 1779 | - | - | 310 | - | - | 3 | - | *E. bieneusi* | [64] |
| Spain | 1 | H | mean age of 75 | yes | - | 47 | - | - | 7 | - | - | 3 | - | *E. bieneusi* | [65] |
| Uganda | 2 | L | 1-60 | yes | - | 152 | - | - | 10 | - | - | 4 | 2002/11-2003/5 | *E. bieneusi* | [23] |
| China | 3 | UM | children | yes | - | 500 | - | - | 1 | - | - | 2 | 2016/6-2016/8 | *E. bieneusi* | [66] |
| Australia | 3 | H | children | yes | - | 279 | 146 | 133 | 7 | 3 | 3 | 2 | 2014/1-2014/12 | *E. bieneusi* | [67] |
|  |  |  | adults | yes | - | 326 | 161 | 165 | 1 | 2 | 0 |  |  |  |  |
| China | 3 | UM | children | yes | - | 93 | 40 | 33 | 11 | 4 | 7 | 3 | 2016 | *E. bieneusi* | [68] |
| Malawi | 3 | L | - | - | - | 35 | - | - | 13 | - | - | 3 | - | *E. bieneusi* | [69] |
| Turkey | 1 | UM | - | yes | - | 200 | - | - | 7 | - | - | 3 | 2012-2013 | *E. bieneusi* | [70] |
|  |  |  |  |  |  | 200 | - | - | 70 | - | - |  |  | *Encephalitozoon* |  |
| **Normal immunity individuals** | | | | | | | | | | | | | | | |
| European | 6 | H | adult | yes | - | 40 | - | - | 4 | - | - | 2 | - | Microsporidia | [71] |
| Thailand | 3 | UM | children | yes | - | 87 | - | - | 13 | - | - | 3 | - | Microsporidia | [17] |
| Spain | 1 | H | - | - | - | 13 | - | - | 1 | - | - | 3 | - | *E. bieneusi* | [65] |
| Uganda | 2 | L | children | no | - | 667 | - | - | 112 | - | - | 3 | - | *E. bieneusi* | [72] |
| Thailand | 3 | UM | children | - | - | 290 | 198 | 92 | 13 | 5 | 7 | 1 | - | *E. bieneusi* | [73] |
| South Africa | 2 | UM | <18 | - | - | 67 | 51 | 49 | 67 | ## | 33 | 4 | 2004/11-2005/5 | *E. bieneusi* | [74] |
| Sweden | 1 | H | - | mix | - | 11 | - | - | 7 | - | - | 2 | 2009/10-2009/11 | *E. bieneusi* | [75] |
| Thailand | 3 | UM | children | - | - | 279 | - | - | 2 | - | - | 2 | 2005/1 | *E. bieneusi* | [76] |
|  |  |  | adult | - |  | 220 | - | - | 5 | - | - |  |  |  |  |
| Thailand | 3 | UM | - | mix | - | 463 | - | - | 61 | - | - | 3 | 2003/6-2004/4 | *E. bieneusi* | [77] |
| China | 3 | UM | children | - | - | 40 | - | - | 9 | - | - | 3 | - | *E. bieneusi* | [78] |
| Czech Republic | 1 | H | 1-84 | mix | - | 382 | - | - | 9 | - | - | 2 | 2008/10-2009/3 | *E. bieneusi* | [79] |
|  |  |  |  |  |  |  | - | - | 136 | - | - |  |  | *Encephalitozoon* |  |
| Germany | 1 | H | - | - | - | 26 | - | - | 2 | - | - | 1 | - | *E. bieneusi* | [80] |
| Portugal | 1 | H | - | - |  | 295 | 178 | 117 | 15 | - | - | 3 | - | *E. bieneusi* | [81] |
| China | 3 | UM | 1-82 | - | - | 683 | 312 | 316 | 29 | - | - | 3 | 2007/3-2007/4， 2010/11 | *E. bieneusi* | [47] |
| Thailand | 3 | UM | children | - | - | 180 | - | - | 6 | - | - | 2 | 2006/11-2007/9 | *E. bieneusi* | [82] |
| São Tomé and Principe | 2 | LM | 1-10 | - | - | 134 | 63 | 71 | 7 | - | - | 2 | 2009/7-2009/9 | *E. bieneusi* | [83] |
| China | 3 | UM | <12 | mix | - | 255 | 124 | 131 | 19 | 8 | 11 | 2 | 2016/6 | *E. bieneusi* | [84] |
| Cameroon | 2 | LM | - | no | - | 196 | - | - | 5 | 0 | 2 | 2 | 2011/3-2011/5 | *E. bieneusi* | [85] |
|  |  |  |  |  |  |  | - | - | 1 | 0 | 1 |  |  | *E. intestinalis* |  |
|  |  |  |  |  |  |  |  |  | 1 | 1 | 0 |  |  | *E. cuniculi* |  |
| Thailand | 3 | UM | - | - | - | 277 | - | - | 15 | - | - | 2 | 2015/6-2015/7 | *E. bieneusi* | [86] |
| India | 3 | LM | - | - | - | 220 | 156 | 64 | 0 | 0 | 0 | 2 | 2005/1 | Microsporidia | [59] |
| Thailand | 3 | UM | - | - | - | 200 | - | - | 2 | - | - | 3 | - | *E. bieneusi* | [87] |
| Thailand | 3 | UM | - | - | - | 697 | 318 | 379 | 15 | 13 | 2 | 4 | 2015/8-2017/1 | *E. bieneusi* | [88] |
| **Organ transplant patient** | | | | | | | | | | | | | | | |
| Iran | 5 | UM | children | no | - | 44 | 23 | 21 | 3 | 3 | 0 | 2 | 2008/9-2011/8 | *E. bieneusi* | [89] |
| Iran | 5 | UM | <70 | - | - | 70 | - | - | 4 | - | - | 3 | - | *Encephalitozoon* | [90] |
|  |  |  |  |  |  | 108 | - | - | 3 | - | - |  |  | *E. bieneusi* |  |
| Iran | 5 | UM | - | no | - | 15 | - | - | 4 | - | - | 2 | - | *E. bieneusi* | [55] |
| Poland | 6 | H | 14-73 | - | - | 86 | - | - | 13 | - | - | 2 | 2013/3-2015/1 | *E. cuniculi* | [91] |
| Iran | 5 | UM | - | - | - | 21 | - | - | 2 | - | - |  | - | *E. bieneusi* | [60] |
|  |  |  |  |  |  |  | - | - | 2 | - | - |  |  | *Encephalitozoon sp.* |  |
| Malaysia | 3 | UM | >18 | - | - | 27 | - | - | 6 | - | - |  | - | *E. bieneusi* | [92] |
| Poland | 6 | H | 21–76 | - | - | 72 | - | - | 6 | 3 | 3 | 2 | 2015-2018 | *E. cuniculi* | [93] |
| **Cancer patients** | | | | | | | | | | | | | | | |
| Iran | 5 | UM | NA | no | - | 234 | - | - | 2 | - | - | 2 | - | *E. bieneusi* | [94] |
| Iran | 5 | UM | - | - | - | 100 | - | - | 21 | - | - | 3 | - | *E. bieneusi* | [60] |
|  |  |  |  |  |  |  |  | - | 3 | - | - |  |  | *Encephali tozoon* |  |
| China | 3 | UM | 25-84 | - | - | 381 | 220 | 161 | 5 | 2 | 3 | 3 | 2016/4-2017/1 | *E. bieneusi* | [95] |
|  |  |  |  | yes |  | 110 | - | - | 4 | - | - |  |  |  |  |
|  |  |  |  | no |  | 270 | - | - | 1 | - | - |  |  |  |  |
| Iran | 5 | UM | <15 | Mix | - | 132 | 77 | 55 | 10 | 10 | 4 | 3 | 2015/5-2016/6 | *E. bieneusi* | [96] |
|  |  |  |  |  | - | 132 | 77 | 55 | 4 | - | - | 3 |  | *E. intestinalis* |  |
| Malaysia | 3 | UM | >18 | - | - | 69 | - | - | 9 | - | - |  | - | *E. bieneusi* | [92] |
| **Other patients** | | | | | | | | | | | | | | |  |
| South Africa | 2 | UM | - | - | - | 255 | 148 | 107 | 100 | 58 | 42 | 4 | 2004/11-2005/5 | *E. bieneusi* | [74] |
| São Tomé and Principe | 2 | LM | 1-10 | - | - | 214 | 123 | 91 | 19 | - | - | 2 | 2009/7-2009/10 | *E. bieneusi* | [83] |
| Iran | 5 | UM | <70 | - | - | 150 | - | - | 3 | - | - | 4 | 2010-2011  - | *Encephalitozoon* | [90] |
|  |  |  |  |  |  | 150 | - | - | 1 | - | - | 4 |  | *E. bieneusi* |  |
| Iran | 5 | UM | NA | - | - | 175 | - | - | 18 | - | - | 0 | - | *E. bieneusi* | [97] |
| Iran | 5 | UM | - | - | - | 87 | - | - | 6 | - | - | 3 | - | *E. bieneusi* | [60] |
|  |  |  |  |  |  |  |  |  | 13 |  |  |  |  | *Encephalitozoon* |  |
| Malaysia | 3 | UM | >18 | - | - | 59 |  |  | 14 |  |  |  | - | *E. bieneusi* | [62] |
| China | 3 | UM | children | - | - | 2284 | - | - | 27 | - | - | 3 | 2016/3-2017/1 | *E. bieneusi* | [98] |

-: Data not available; yes: Individuals have diarrhea; no: No symptoms of diarrhea; L: Low-income country; LM: Lower middle-income; UM: Upper middle-income; H: Hight-income country;

Region^*^: 1: Western and central Europe and North America; 2: Sub-Saharan Africa; 3: Asia and the Pacific; 4: Latin America and the Caribbean; 5: Middle East and North Africa; 6: Eastern Europe and central Asia.

**Table S2. Included studies of microsporidian coinfection in humans**

| **Country** | **Author** | **Year** | **Age** | **No. of individuals with pathogen infection** | **No. of individuals** | **Pathogen species** | **Quality score** | **Reference** |
| --- | --- | --- | --- | --- | --- | --- | --- | --- |
| Italy | Brandonisio et al. | \| 19999 \| NA \| \| --- \| --- \| | NA | 3 | 154 | C*ryptosporidium parvum* + microsporidia | 3 | [12] |
| Thailand | Chokephaibulkit et al. | 2001 | Children | 9 | 82 | *Cryptosporidium +* microsporidia | 3 | [10] |
| Chennai | S Kumar et al. | 2002 | - | 1 | 59 | *Microsporidia + Cyclospora cayetanensis* | 3 | [18] |
| Uganda | JAMES K. TUMWINE et al. | 2005 | < 60 mouths | 67 | 243 | *E. bieneusi+ Cryptosporidium* | 4 | [99] |
| Haiti | Christian P Raccurt et al. | 2008 | - | 3 | 58 | *E. bieneusi + Cryptosporidium* | 3 | [100] |
|  |  |  |  | 1 | 58 | *E. bieneusi* *+ Cyclospora cayetanensis* |  |  |
| Argentina | Jorge Nétor Velásquez et al. | 2012 | - | 1 | 11 | *Cryptosporidium hominis* *+ E. bieneusi* | 1 | [101] |
| China | Hua Liu et al. | 2014 | < 77 years | 9 | 252 | *Cryptosporidium + E. bieneusi* | 4 | [102] |
| Poland | Małgorzata Bednarska et al. | 2014 | - | 1 | 80 | *Cryptosporidium + E. bieneusi* | 3 | [103] |
| Iran | Ali Taghipour et al. | 2019 | - | 1 | 161 | *Blastocystis+* microsporidia | 4 | [104] |

-: Data not available; +: Co-infection

**Table S3. Included studies of microsporidian infection in swine**

| **Country** | **Author** | **Year** | **Host type** | **No. of samples** | **No. of positive** | **Microsporidian species** | **Collection date** | **Quality score** | **Reference** |
| --- | --- | --- | --- | --- | --- | --- | --- | --- | --- |
| **Pre-weaned pigs** | | | | | | | | | |
| Korea | Jeong DK et al. | 2007 | diarrheic | 472 | 67 | *E. bieneusi* | - | 3 | [105] |
| Korea | Jeong DK et al. | 2007 | asymptomatic | 117 | 6 | *E. bieneusi* | - | 3 | [105] |
| Czech R | Sak B et al. | 2008 | diarrheic, asymptomatic | 48 | 37 | *E. bieneusi* | 2006/9-2006/11 | 3 | [106] |
| Thailand | Leelayoova S et al. | 2009 | asymptomatic | 29 | 1 | *E. bieneusi* | - | 2 | [107] |
| China | Li W et al. | 2014 | asymptomatic | 33 | 21 | *E. bieneusi* | 2012/9- 2013/4 | 3 | [108] |
| China | Li W et al. | 2014 | asymptomatic | 28 | 1 | *E. bieneusi* | - | 3 | [109] |
| China | Li W et al. | 2014 | asymptomatic | 71 | 53 | *E. bieneusi* | - | 3 | [110] |
| China | Wan Q et al. | 2016 | asymptomatic | 162 | 94 | *E. bieneusi* | - | 3 | [111] |
| China | Zou Y et al. | 2018 | asymptomatic | 87 | 59 | *E. bieneusi* | 2016/3- 2016/6 | 4 | [112] |
| China | Ning Zhang et al. | 2019 | asymptomatic | 111 | 40 | *E. bieneusi* | 2018/4-2018/10 | 3 | [113] |
| China | Li DF et al. | 2019 | asymptomatic | 169 | 60 | *E. bieneusi* | 2017/9-2018/6 | 3 | [114] |
| **Post-weaned pigs** | | | | | | | | | |
| Korea | Jeong DK et al. | 2007 | asymptomatic | 78 | 16 | *E. bieneusi* | - | 3 | [105] |
| Czech R | Sak B et al. | 2008 | diarrheic, asymptomatic | 16 | 15 | *E. bieneusi* | 2006/9-2006/11 | 3 | [106] |
| Thailand | Leelayoova S et al. | 2009 | asymptomatic | 40 | 3 | *E. bieneusi* | - | 2 | [107] |
| China | Li W et al. | 2014 | asymptomatic | 61 | 25 | *E. bieneusi* | 2012/9- 2013/4 | 3 | [108] |
| China | Li W et al. | 2014 | asymptomatic | 20 | 7 | *E. bieneusi* | - | 3 | [109] |
| China | Li W et al. | 2014 | asymptomatic | 33 | 22 | *E. bieneusi* | - | 3 | [110] |
| China | Wan Q et al. | 2016 | asymptomatic | 113 | 54 | *E. bieneusi* | - | 3 | [111] |
| China | Wang SS et al. | 2018 | asymptomatic | 155 | 125 | *E. bieneusi* | 2016/9-2017/3 | 2 | [115] |
| China | Ning Zhang et al. | 2019 | asymptomatic | 127 | 45 | *E. bieneusi* | 2018/4-2018/10 | 3 | [113] |
| China | Li DF et al. | 2019 | asymptomatic | 280 | 217 | *E. bieneusi* | 2017/9-2018/6 | 3 | [114] |
| **Growing pigs** | | | | | | | | | |
| Thailand | Leelayoova S et al. | 2009 | asymptomatic | 144 | 34 | *E. bieneusi* | - | 2 | [107] |
| China | Li W et al. | 2014 | asymptomatic | 19 | 5 | *E. bieneusi* | 2012/9-2013/4 | 3 | [108] |
| China | Li W et al. | 2014 | asymptomatic | 16 | 3 | *E. bieneusi* | - | 3 | [109] |
| China | Li W et al. | 2014 | asymptomatic | 53 | 19 | *E. bieneusi* | - | 3 | [110] |
| China | Zhao W et al. | 2014 | asymptomatic | 95 | 85 | *E. bieneusi* | - | 4 | [109] |
| China | Wan Q et al. | 2016 | asymptomatic | 288 | 114 | *E. bieneusi* | - | 3 | [111] |
| China | Zou Y et al. | 2018 | asymptomatic | 73 | 12 | *E. bieneusi* | 2016/3- 2016/6 | 4 | [112] |
| China | Wang SS et al. | 2018 | asymptomatic | 220 | 189 | *E. bieneusi* | 2016/9-2017/3 | 2 | [115] |
| China | Ning Zhang et al. | 2019 | asymptomatic | 104 | 28 | *E. bieneusi* | 2018/4-2018/10 | 3 | [113] |
| China | Li DF et al. | 2019 | asymptomatic | 128 | 87 | *E. bieneusi* | 2017/9-2018/6 | 3 | [114] |
| **Adult pigs** | | | | | | | | | |
| Slovakia | Valencáková A et al. | 2005 | asymptomatic | 27 | 25 | *E. intestinalis* | - | 2 | [116] |
| Czech R | Sak B et al. | 2008 | diarrheic, asymptomatic | 15 | 12 | *E. bieneusi* | 2006/9-2006/11 | 3 | [106] |
| Thailand | Leelayoova S et al. | 2009 | asymptomatic | 38 | 2 | *E. bieneusi* | - | 2 | [107] |
| China | Zou Y et al. | 2018 | asymptomatic | 236 | 54 | *E. bieneusi* | 2016/3- 2016/6 | 4 | [112] |
| China | Wang SS et al. | 2018 | asymptomatic | 57 | 27 | *E. bieneusi* | 2016/9-2017/3 | 2 | [115] |
| China | Ning Zhang et al. | 2019 | asymptomatic | 255 | 62 | *E. bieneusi* | 2018/4-2018/10 | 3 | [113] |
| China | Li DF et al. | 2019 | asymptomatic | 222 | 25 | *E. bieneusi* | 2017/9-2018/6 | 3 | [114] |
| **Wild boars (*Sus scrofa*)** | | | | | | | | | |
| Austria | Němejc K et al. | 2014 | asymptomatic | 460 | 33 | *E. bieneusi* | 2011-2012 | 4 | [117] |
|  |  |  |  |  | 16 | *Encephalitozoon cuniculi* |  |  |  |
|  |  |  |  |  | 5 | *Encephalitozoon cuniculi + E. bieneusi* |  |  |  |
| China | Li W et al. | 2017 | asymptomatic | 357 | 147 | *E. bieneusi* | 2014-2015 | 3 | [118] |
| China | Wang SS et al. | 2018 | asymptomatic | 30 | 20 | *E. bieneusi* | 2016/9-2017/3 | 2 | [115] |
| China | Ning Zhang et al. | 2019 | asymptomatic | 28 | 2 | *E. bieneusi* | 2018/4-2018/10 | 3 | [113] |
| China | Feng SG et al. | 2020 | asymptomatic | 257 | 108 | *E. bieneusi* | 2015/9-2018/11 | 3 | [119] |
| **Tibetan pig** | | | | | | | | | |
| China | Luo R et al. | 2019 | asymptomatic | 266 | 83 | *E. bieneusi* | 2017/6-2017/10 | 2 | [120] |
| China | Li D et al. | 2019 | asymptomatic | 1190 | 645 | *E. bieneusi* | 2016/6-201612 | 3 | [121] |
| China | Zou Y et al. | 2019 | asymptomatic | 345 | 41 | *E. bieneusi* | - | 4 | [122] |

-: Data not available

**Table S4. Included studies of microsporidian infection in cats and dogs**

| **Country** | **Year** | **Author** | **Origin** | **Host** | **No. of samples** | **No. of positive** | **Microsporidian species** | **Collection date** | **Quality score** | **Reference** |
| --- | --- | --- | --- | --- | --- | --- | --- | --- | --- | --- |
| Germany | 2001 | Xu H et al. | domestic | cat | 60 | 3 | *E. bieneusi* | - | 2 | [80] |
| Colombia | 2006 | Santín M et al. | feral | cat | 46 | 8 | *E. bieneusi* | 2005/8 | 4 | [123] |
| Thailand | 2013 | Mori H et al. | domestic | cat | 80 | 25 | *E. bieneusi* | 2012/1-2014/4 | 3 | [124] |
| China | 2014 | Karim MR et al. | domestic | cat | 96 | 11 | *E. bieneusi* | 2013/1,2014/2 | 3 | [125] |
| China | 2015 | Li W et al. | domestic | cat | 52 | 3 | *E. bieneusi* | 2013/6 | 4 | [126] |
| China | 2015 | Askari Z et al. | domestic | cat | 26 | 3 | *E. bieneusi* | 2012-2013 | 3 | [127] |
| China | 2016 | Xu H et al. | domestic | cat | 160 | 9 | *E. bieneusi* | 2011-2014 | 2 | [128] |
| Egypt | 2016 | Al-Herrawy AZ et al. | domestic | cat | 104 | 13 | *E. bieneusi* | 2012-2013 | 2 | [129] |
|  |  |  |  |  | 104 | 5 | *E. intestinalis* | 2012-2013 |  |  |
| Japan | 2016 | Tsukada R et al. | feral | cat | 132 | 11 | *E. cuniculi* | - | 2 | [130] |
|  |  |  | domestic | cat | 163 | 7 |  | - |  |  |
| Poland | 2017 | Kvac M et al. | domestic | cat | 31 | 0 | *E. bieneusi* | - | 3 | [131] |
|  |  |  | feral | cat | 33 | 4 |  | - |  |  |
| Poland | 2017 | Piekarska J et al. | domestic | cat | 44 | 4 | *E. bieneusi* | 2013/6 -2016/12 | 2 | [132] |
| Slovakia | 2017 | Kvac M et al. | domestic | cat | 34 | 0 | *E. bieneusi* | - | 3 | [131] |
|  |  |  | feral | cat | 39 | 5 |  | - |  |  |
| Czech R. | 2017 | Kvac M et al. | domestic | cat | 55 | 0 | *E. bieneusi* | - | 3 | [131] |
|  |  |  | feral | cat | 63 | 3 |  | - |  |  |
| Spain | 2019 | Dashti A et al. | domestic | cat | 99 | 3 | *E. bieneusi* | - | 3 | [133] |
| Brazil | 2019 | Prado JBF et al. | domestic | cat | 60 | 2 | *E. bieneusi* | - | 3 | [134] |
| Japan and Turkey | 2019 | Pekmezci D | domestic | cat | 72 | 4 | *E. bieneusi* | 2015/5 -2015/12 | 3 | [135] |
|  |  |  |  |  | 72 | 3 | *Encephalitozoon* | 2015/5 -2015/12 |  |  |
| Switzerland | 1999 | Mathis A et al. | domestic | dog | 36 | 3 | *E. bieneusi* | NA | 3 | [136] |
| Colombia | 2008 | Santín M et al. | feral | dog | 120 | 18 | *E. bieneusi* | NA | 2 | [137] |
| Japan | 2009 | Abe Nc et al. | domestic | dog | 20 | 1 | *E. bieneusi* | NA | 2 | [138] |
|  |  |  | feral | dog | 59 | 1 |  | NA |  |  |
| China | 2011 | Zhang X et al. | domestic | dog | 26 | 2 | *E. bieneusi* | NA | 2 | [78] |
| Spain | 2014 | Galván-Díaz AL et al. | domestic | dog | 73 | 7 | *E. bieneusi* | NA | 2 | [139] |
| China | 2014 | Karim MR et al. | domestic | dog | 197 | 23 | *E. bieneusi* | 2013/1-2014/2 | 3 | [125] |
|  |  |  | feral | dog | 151 | 31 |  | 2013/1-2014/2 |  |  |
| China | 2015 | Li W et al. | domestic | dog | 267 | 18 | *E. bieneusi* | 2013/3-2013/7 | 4 | [126] |
| China | 2016 | Xu H et al. | domestic | dog | 102 | 8 | *E. bieneusi* | 2011-2014 | 3 | [128] |
|  |  |  | domestic | dog | 383 | 21 | *E. bieneusi* | 2011-2014 |  |  |
| Egypt | 2016 | Al-Herrawy AZ et al. | domestic | dog | 108 | 14 | *E. bieneusi* | 2012-2013 | 2 | [129] |
|  |  |  |  |  | 108 | 19 | *E. intestinalis* | 2012-2013 |  |  |
| Poland | 2017 | Piekarska J et al. | domestic | dog | 82 | 4 | *E. bieneusi* | 2013/6-2016/12 | 2 | [132] |
| Spain | 2019 | Dashti A et al. | domestic | dog | 237 | 2 | *E. bieneusi* | 2014-2016 | 3 | [133] |
| Iran | 2019 | Delrobaei M et al. | feral | dog | 75 | 4 | *E. bieneusi* | 2015/3-2015/10 | 3 | [140] |
| Japan | 2019 | Duzlu O et al. | domestic | dog | 282 | 35 | *E. intestinalis* | 2011-2013 | 4 | [141] |
|  |  |  |  |  |  | 6 | *E. cuniculi* |  |  |  |
| Japan | 2019 | Phrompraphai T et al. | domestic | dog | 597 | 26 | *E. bieneusi* | 2013/10-2016/12 | 3 | [142] |

-: Data not available.

**Table S5. Included studies of microsporidian infection in ruminants**

| **Country** | **Author** | **Year** | **Host** | **Microsporidian species** | **No. of samples** | **No. of positive** | **Sampling date** | **Quality score** | **Reference** |
| --- | --- | --- | --- | --- | --- | --- | --- | --- | --- |
| Germany | Rinder H et al. | 2000 | cattle | *E. bieneusi* | 28 | 3 | - | 3 | [143] |
| USA | Sulaiman IM et al. | 2004 | cattle | *E. bieneusi* | 290 | 29 | - | 3 | [144] |
| Portugal | Sulaiman IM et al. | 2004 | cattle | *E. bieneusi* | 48 | 3 | - | 3 | [144] |
| USA | Santín M et al. | 2005 | dairy cattle | *E. bieneusi* | 571 | 131 | - | 3 | [145] |
| USA | Fayer R et al. | 2007 | dairy cattle | *E. bieneusi* | 541 | 24 | - | 3 | [146] |
| Korea | Lee JH et al. | 2007 | cattle | *E. bieneusi* | 538 | 80 | 2004/9-2005/8 | 4 | [147] |
| Korea | Lee JH et al. | 2008 | dairy cattle | *E. bieneusi* | 180 | 15 | - | 3 | [148] |
| China | Zhang X et al. | 2011 | cattle | *E. bieneusi* | 93 | 35 | - | 3 | [78] |
| USA | Fayer R et al. | 2012 | dairy cattle | *E. bieneusi* | 47 | 17 | - | 3 | [149] |
| USA | Santín M et al. | 2012 | dairy cattle | *E. bieneusi* | 819 | 285 | 2007-2008 | 3 | [150] |
| Czech R | Juránková J et al. | 2013 | dairy cattle | *E. bieneusi* | 240 | 37 | - | 3 | [151] |
| Argentina | Del Coco VF et al. | 2014 | dairy cattle | *E. bieneusi* | 70 | 10 | 2008 - 2010 | 3 | [152] |
| China | Jiang Y et al. | 2015 | dairy cattle | *E. bieneusi* | 537 | 32 | 2013/9-2014/10 | 3 | [127] |
| China | Ma J et al. | 2015 | dairy cattle | *E. bieneusi* | 446 | 144 | 2012/5-2012/7 | 3 | [153] |
| China | Ma J et al. | 2015 | beef cattle | *E. bieneusi* | 166 | 44 | 2012/5-2012/7 | 3 | [153] |
| China | Zhao W et al. | 2015 | dairy cattle | *E. bieneusi* | 133 | 40 | 2013/10 | 3 | [154] |
| China | Askari Z et al. | 2015 | cattle | *E. bieneusi* | 39 | 2 | 2012-2013 | 3 | [127] |
| Brazil | da Silva Fiuza VR et al. | 2016 | dairy cattle, beef cattle | *E. bieneusi* | 452 | 79 | - | 3 | [155] |
| China | Li J et al. | 2016 | dairy cattle | *E. bieneusi* | 879 | 214 | 2014/6- 2015/1 | 3 | [156] |
| China | Wang XT et al. | 2016 | dairy cattle, beef cattle | *E. bieneusi* | 371 | 70 | 2012/1-2014/11 | 2 | [157] |
| Egypt | Al-Herrawy AZ et al. | 2016 | cattle | *E. bieneusi* | 98 | 14 | - | 3 | [158] |
| China | Hu S et al. | 2017 | dairy cattle | *E. bieneusi* | 1040 | 202 | - | 2 | [159] |
| China | Qi M et al. | 2017 | dairy cattle | *E. bieneusi* | 514 | 85 | 2013/8-2013/9 | 3 | [160] |
| China | Tang C et al. | 2018 | pre-weaned calve | *E. bieneusi* | 809 | 214 | 2015/4-2016/3 | 2 | [161] |
| Iran | Kord-Sarkachi E et al. | 2018 | cow | *E. bieneusi* | 256 | 31 | - | 3 | [162] |
| China | Feng Y et al. | 2019 | dairy calve | *E. bieneusi* | 388 | 16 | 2017/3-2018/1 | 3 | [163] |
| China | Yu F et al. | 2019 | dairy cattle | *E. bieneusi* | 277 | 33 | - | 3 | [164] |
| Australia | Zhang Y et al. | 2019 | cattle | *E. bieneusi* | 471 | 49 | 2011/11-2012/4 | 3 | [165] |
| Thailand | Udonsom R et al. | 2019 | cattle | *E. bieneusi* | 60 | 3 | - | 3 | [166] |
| China | Zhao W et al. | 2019 | cattle | *E. bieneusi* | 30 | 1 | 2014/4-2017/6 | 3 | [167] |
| China | Zhang Q et al. | 2019 | cattle | *E. bieneusi* | 57 | 10 | - | 3 | [168] |
| China | Wang R et al. | 2019 | dairy cattle | *E. bieneusi* | 1366 | 171 | 2016/4-2017/11 | 3 | [169] |
| China | Tao WF et al. | 2020 | dairy calve | *E. bieneusi* | 321 | 93 | 2017/9-2018/12 | 3 | [170] |
| The Republic of South Africa | Abu Samra N et al. | 2012 | African buffalo (*Syncerus caffer*) | *E. bieneusi* | 50 | 9 | - | 4 | [171] |
| China | Ma J et al. | 2015 | dater buffalos | *E. bieneusi* | 181 | 43 | 2012/5-2012/7 | 3 | [153] |
| Egypt | Al-Herrawy AZ et al. | 2016 | buffalo | *E. bieneusi, E. intestinalis* | 116 | 5 | - | 2 | [158] |
| China | Yayun Wu et al. | 2019 | yak (*Bos grunniens*) | *E. bieneusi* | 577 | 8 | 2016/6-2016/7 | 3 | [172] |
| China | Ma J et al. | 2015 | yak (*Bos grunniens*) | *E. bieneusi* | 327 | 23 | 2013/5-2013/7 | 3 | [173] |
| China:Tibetan | Ma JG et al. | 2017 | yak (*Bos grunniens*) | *E. bieneusi* | 353 | 4 | 2013/6-2016/10 | 3 | [174] |
| China | Zhang Q et al. | 2018 | white yak (*Bos grunniens*) | *E. bieneusi* | 554 | 40 | 2013/6-2015/5 | 3 | [175] |
| China | Zhang Q et al. | 2019 | yak (*Bos grunniens*) | *E. bieneusi* | 101 | 13 | 2018/5-2018/7 | 4 | [168] |
| China | Falei Li et al. | 2019 | horse | *E. bieneusi* | 325 | 24 | 2015-2019 | 3 | [176] |
| China | Zhang Q et al. | 2019 | horse | *E. bieneusi* | 32 | 5 | 2018/5-2018/7 | 2 | [168] |
| Algeria | Laatamna AE et al. | 2015 | horse | *E. bieneusi* | 219 | 15 | - | 3 | [177] |
| Algeria | Laatamna AE et al. | 2015 | horse | *E. cuniculi* | 219 | 4 | - | 3 | [177] |
| Colombia | Santín M et al. | 2010 | horse | *E. bieneusi* | 195 | 21 | 2007/8-2007/10 | 3 | [178] |
| Czech R. | Wagnerová P et al. | 2012 | horse | *E. bieneusi* | 377 | 66 | - | 3 | [179] |
| China | Deng L et al. | 2016 | horse | *E. bieneusi* | 333 | 75 | 2015/8-2016/4 | 4 | [180] |
| China | Wagnerová P et al. | 2016 | mustang, Chincoteague ponies | *E. bieneusi* | 84 | 35 | - | 3 | [181] |
| China | Zhao A et al. | 2019 | racehorse | *E. bieneusi* | 621 | 30 | 2012/5-2012/7 | 3 | [182] |
| China | Falei Li et al. | 2019 | donkey (*Equus asinus*) | *E. bieneusi* | 540 | 118 | 2015-2019 | 3 | [176] |
| Algeria | Laatamna AE et al. | 2015 | donkey (*Equus asinus*) | *E. bieneusi* | 124 | 15 | 2011/11-2013/5 | 3 | [177] |
| Algeria | Laatamna AE et al. | 2015 | donkey (*Equus asinus*) | *E. cuniculi* | 124 | 2 | 2011/11-2013/5 | 2 | [177] |
| China | Yue DM et al. | 2017 | donkey (*Equus asinus*) | *E. bieneusi* | 301 | 16 | 2015/5-2016/10 | 3 | [183] |
| China | Qi M et al. | 2018 | camel | *E. bieneusi* | 407 | 122 | 2013/8-9,2016/7-2017/7 | 3 | [184] |
| China | Zhang Q et al. | 2019 | alpacas (*Vicugna pacos*) | *E. bieneusi* | 185 | 28 | 2016/8-2017/3 | 3 | [185] |
| Sweden | Stensvold CR et al. | 2014 | sheep | *E. bieneusi* | 109 | 49 | - | 2 | [186] |
| China | Li W et al. | 2014 | sheep | *E. bieneusi* | 45 | 2 | 2012/10-2012/12 | 2 | [187] |
| China | Li W et al. | 2015 | lamb | *E. bieneusi* | 87 | 65 | - | 3 | [188] |
| China | Jiang Y et al. | 2015 | sheep | *E. bieneusi* | 489 | 68 | 2013-2014 | 4 | [189] |
| China | Li J et al. | 2015 | sheep | *E. bieneusi* | 27 | 2 | 2013/2-3,2015/3-5 | 2 | [190] |
| China | Li W et al. | 2015 | sheep | *E. bieneusi* | 154 | 98 | - | 3 | [188] |
| China | Zhao W et al. | 2015 | sheep | *E. bieneusi* | 138 | 31 | 2013/6-2014/5 | 3 | [191] |
| Brazil | Fiuza VRDS et al. | 2016 | sheep | *E. bieneusi* | 125 | 24 | - | 3 | [192] |
| China | Shi K et al. | 2016 | sheep | *E. bieneusi* | 414 | 177 | 2011/3-2013/10 | 3 | [193] |
| China | Wei Zhao et al. | 2018 | sheep | *E. bieneusi* | 37 | 10 | 2014/4-2017/6 | 3 | [167] |
| China | Yang H et al. | 2018 | sheep | *E. bieneusi* | 953 | 194 | 2013/6-2015/9 | 3 | [194] |
| China | Wu Y et al. | 2018 | sheep | *E. bieneusi* | 177 | 61 | 2015/3-2015/7 | 2 | [195] |
| China | Zhang Q et al. | 2018 | sheep | *E. bieneusi* | 312 | 73 | 2013/6- 2015/5 | 3 | [175] |
| China | Li WC et al. | 2019 | sheep | *E. bieneusi* | 832 | 28 | 2015/9-2015/12 | 4 | [196] |
| China | Qi M et al. | 2019 | sheep | *E. bieneusi* | 318 | 20 | 2015/8-2017/9 | 4 | [197] |
| China | Peng JJ et al. | 2019 | sheep | *E. bieneusi* | 1014 | 124 | - | 4 | [198] |
| China | Zhang Q et al. | 2019 | sheep | *E. bieneusi* | 116 | 23 | 2018/5-2018/7 | 3 | [168] |
| China | Chang Y et al. | 2020 | sheep | *E. bieneusi* | 620 | 93 | 2016/6-2016/7 | 4 | [199] |
| Rwanda | Sak B et al. | 2014 | mountain gorillas | microsporidia | 140 | 11 | 2007/9 | 3 | [200] |
| China | Zhao W et al. | 2015 | goat | *E. bieneusi* | 55 | 12 | 2013/6-2014/5 | 3 | [191] |
| China | Peng XQ et al. | 2016 | goat | *E. bieneusi* | 629 | 274 | 2014/8-2015/6 | 3 | [201] |
|  |  |  | goat | *E. bieneusi* | 537 | 131 | 2014/8-2015/6 |  |  |
| China | Shi K et al. | 2016 | goat | *E. bieneusi* | 611 | 176 | 2011/3-2013/10 | 3 | [193] |
| Thailand | Udonsom R et al. | 2019 | goat | *E. bieneusi* | 73 | 14 | - | 3 | [166] |
| China | Li WC et al. | 2019 | goat | *E. bieneusi* | 781 | 32 | 2015/9-2015/12  - | 2 | [196] |
| China | Chang Y et al. | 2019 | goat | *E. bieneusi* | 260 | 25 | 2016/6-2016/7 | 3 | [199] |
| China | Zhang Q et al. | 2019 | goat | *E. bieneusi* | 59 | 11 | 2018/5-2018/7 | 3 | [202] |
| China:Hainan | Zhou HH et al. | 2019 | black goat | *E. bieneusi* | 341 | 82 | - | 3 | [203] |
| china | Teklu Wegayehu1 | 2020 | lamb | *E. bieneusi* | 389 | 39 | - | 4 | [204] |
| China | Zhao W et al. | 2014 | deer | *E. bieneusi* | 91 | 29 | 2012/5-2013/10 | 3 | [205] |
| USA | Yaqiong Guo et al. | 2014 | deer | *E. bieneusi* | 49 | 6 | 2005/9-2007/7 | 2 | [206] |
| USA | Monica Santin et al. | 2014 | white-tailed deer | *E. bieneusi* | 80 | 26 | 2010-2012 | 3 | [207] |
| China | Liu W et al. | 2015 | deer | *E. bieneusi* | 125 | 21 | 2014/10 | 3 | [208] |
| China | Zhang Z et al. | 2015 | deer | *E. bieneusi* | 47 | 16 | 2014/3 | 3 | [209] |
| China | Li W et al. | 2016 | deer | *E. bieneusi* | 28 | 6 | 2014/6-2015/9 | 3 | [210] |
| China | Zhang XX et al. | 2016 | deer | *E. bieneusi* | 326 | 23 | 2014 | 3 | [211] |
| China | Huang J et al. | 2017 | deer | *E. bieneusi* | 615 | 221 | 2014/4-2014/8 | 3 | [212] |
| China | Zhao W et al. | 2017 | deer | *E. bieneusi* | 122 | 10 | 2015/11-2016/3 | 3 | [213] |
| Australia | Zhang Y et al. | 2018 | deer | *E. bieneusi* | 610 | 25 | 2009/6-2017/3 | 4 | [214] |
| China | Song Y et al. | 2018 | musk deer | *E. bieneusi* | 223 | 38 | 2017/2 | 4 | [215] |
| Korea | Amer et al. | 2019 | water deer | *E. bieneusi* | 97 | 52 | 2018/4-2018/9 | 3 | [216] |
| China | Xie F et al. | 2019 | Père David's deer | *E. bieneusi* | 128 | 45 | 2018/7 | 3 | [217] |
| China | Tao WF et al. | 2020 | sika deer | *E. bieneusi* | 818 | 111 | - | 3 | [170] |
| China | Qi M et al. | 2018 | camel | *E. bieneusi* | 407 | 122 | 2016/7-2017/7 | 4 | [184] |
| Algeria | Djamel Baroudi et al. | 2018 | camel | *E. bieneusi* | 39 | 8 | 2012/1-2013/1 | 3 | [218] |
| Australia | Koehler AV et al. | 2018 | alpaca (*Vicugna pacos*) | *E. bieneusi* | 81 | 8 | 2016/1-2017/7 | 3 | [219] |
| China | Zhang Q et al. | 2019 | camel | *E. bieneusi* | 40 | 18 | 2018/5-2018/7 | 3 | [202] |
| China:Shanxi | Ma YT wt al. | 2019 | alpaca (*Vicugna pacos*) | *E. bieneusi* | 366 | 16 | - | 4 | [220] |

-: Data not available.

**Table S6. Included studies of microsporidian infection in nonhuman primates**

| **Country** | **Year** | **Author** | **Source** | **Host** | **No. of positive** | **No. of samples** | **Species** | **Sampling date** | **Quality score** | **Reference** |
| --- | --- | --- | --- | --- | --- | --- | --- | --- | --- | --- |
| Europe and Africa | 2011 | Sak B et al. | zoo | non-human primate | 46 | 150 | *E. cuniculi* | 2005-2009 | 3 | [221] |
|  |  |  |  |  | 2 | 159 | *E. hellen* |  |  |  |
|  |  |  |  |  | 4 | 150 | *E. bieneusi* |  |  |  |
| Kenya | 2011 | Li W et al. | forest | baboon | 29 | 235 | *E. bieneusi* | 2006/6-2008/12 | 3 | [222] |
| China | 2012 | Ye J et al. | park | monkey | 116 | 411 | *E. bieneusi* | 2010/11 | 2 | [223] |
| Central African Republic | 2013 | Petrzelkova KJ et al. | forest | gorilla gorilla gorilla | 8 | 201 | *E. bieneusi* | 2007/11-2007/12, 2008/11-2008/12, 2009/11-2009/12, 20010/11-2010/12 | 4 | [224] |
|  |  |  |  |  | 15 | 201 | *Encephalitozoon* |  |  |  |
| Rwanda | 2014 | Sak B et al. | park | baboon | 18 | 100 | *E. bieneusi* | 2007/9 | 3 | [200] |
|  |  |  |  |  | 11 | 100 | *E. cuniculi* |  |  |  |
| China | 2014 | Ye J et al. | lab | monkey | 38 | 205 | *E. bieneusi* | 2011/12 | 3 | [225] |
| China | 2014 | Karim MR et al. | farm | non-human primate | 97 | 746 | *E. bieneusi* | 2013-2019 | 3 | [226] |
|  |  |  | zoo | non-human-primate | 12 | 163 |  |  |  |  |
|  |  |  | lab | non-human primate | 31 | 117 |  |  |  |  |
|  |  |  | free range | non-human primate | 18 | 360 |  |  |  |  |
| China | 2014 | Md RobiulKarima et al. | - | *Macaca fascicularis* | 42 | 62 | *E. bieneusi* | 2009-2012 | 4 | [227] |
|  |  |  |  | *Macaca mulatta* | 62 | 521 |  |  |  |  |
|  |  |  |  | Macaca fuscata | 3 | 9 |  |  |  |  |
|  |  |  |  | Presbytis leucocephalus | 19 | 147 |  |  |  |  |
| China | 2015 | Du SZ et al. | captive wild animal farms | Cercopithecus neglectus | 1 | 4 | *E. bieneusi* | 2013/12-2014/7 | 3 | [228] |
|  |  |  |  | *Papio hamadrayas* | 14 | 86 |  |  |  |  |
|  |  |  |  | M. mulatta | 2 | 12 |  |  |  |  |
|  |  |  |  | *S. sciureus* | 6 | 63 |  |  |  |  |
|  |  |  |  | rhinopithecus roxellana | 2 | 20 |  |  |  |  |
| China | 2015 | Karim MR et al. | zoo | rhesus macaque | 33 | 106 | *E. bieneusi* | 2013/10-2014/1 | 3 | [229] |
|  |  |  |  | cynomolgus monkey | 5 | 18 |  |  |  |  |
|  |  |  |  | pigtailed macaque | 4 | 16 |  |  |  |  |
|  |  |  |  | celebes crested macaque | 1 | 2 |  |  |  |  |
|  |  |  |  | hussar monkey | 8 | 16 |  |  |  |  |
|  |  |  |  | trachypithecus francoisi | 3 | 10 |  |  |  |  |
|  |  |  |  | golden monkey | 9 | 29 |  |  |  |  |
|  |  |  |  | king colobus | 1 | 7 |  |  |  |  |
|  |  |  |  | Chlorocebus sabaeus (green monkey) | 10 | 15 |  |  |  |  |
|  |  |  |  | mandrillus sphinx | 1 | 23 |  |  |  |  |
|  |  |  |  | hamadryas baboon | 6 | 21 |  |  |  |  |
|  |  |  |  | olive baboon | 2 | 5 |  |  |  |  |
|  |  |  |  | blackcapped capuchin | 6 | 22 |  |  |  |  |
|  |  |  |  | weeper capuchin | 3 | 4 |  |  |  |  |
|  |  |  |  | whitefronted capuchin | 1 | 5 |  |  |  |  |
|  |  |  |  | squirrel monkey | 17 | 43 |  |  |  |  |
|  |  |  |  | marmoset | 3 | 5 |  |  |  |  |
|  |  |  |  | Aotus sp. | 3 | 4 |  |  |  |  |
|  |  |  |  | ring-tailed lemur | 11 | 45 |  |  |  |  |
|  |  |  |  | blackand-white ruffed lemur | 2 | 5 |  |  |  |  |
|  |  |  |  | northern white-cheeked gibbon | 5 | 14 |  |  |  |  |
|  |  |  |  | silvery gibbon | 3 | 3 |  |  |  |  |
|  |  |  |  | whitehanded gibbon | 5 | 8 |  |  |  |  |
|  |  |  |  | bornean orangutan | 4 | 23 |  |  |  |  |
|  |  |  |  | common chimpanzee | 2 | 14 |  |  |  |  |
| China | 2016 | Deng L | zoo | ring-tailed lemur | 1 | 6 | *E. bieneusi* | 2014/6-2015/9 | 3 | [210] |
|  |  |  |  | northern white-cheeked gibbon | 1 | 2 |  |  |  |  |
|  |  |  |  | Papio anubis | 4 | 8 |  |  |  |  |
|  |  |  |  | golden snub-nosed monkey | 2 | 15 |  |  |  |  |
| China | 2016 | Mynářová A et al. | forest | Pongo abelii | 3 | 90 | *E. bieneusi* | 2013/12-2014/7 |  | [230] |
|  |  |  |  | Pongo pygmaeus | 3 | 208 |  |  |  |  |
| China | 2017 | Yang H | lab | macaca.fascicularis | 34 | 133 | *E. bieneusi* | 2015/10 | 3 | [231] |
|  |  |  |  | macaca mulatta | 3 | 72 | *E. bieneusi* |  |  |  |
| China | 2017 | Yu F et al. | zoo | golden snub-nosed monkey | 74 | 160 | *E. bieneusi* | 2015/6-2015/11 | 3 | [232] |
|  |  |  | farm, zoo | macaca mulatta | 26 | 303 |  |  |  |  |
|  |  |  | zoo | northern white-cheeked gibbon | 20 | 30 |  |  |  |  |
| China | 2019 | Chen L et al. | zoo | crab-eating macaque | 461 | 1452 | *E. bieneusi* | 2016/4, 2017/6, 2017/10, 2018/1 | 4 | [233] |
| China | 2020 | Zhao W et al. | zoo | macaca fascicularis | 59 | 193 | *E. bieneusi* | 2018/7-2018/8 | 3 | [234] |

-: Data not available

**Table S7. Included studies of microsporidian infection in other mammals**

| **Country** | **Author** | **Year** | **Host** | **No. of samples** | **No. of positive** | **Microsporidian species** | **Sampling date** | **Quality score** | **Reference** |
| --- | --- | --- | --- | --- | --- | --- | --- | --- | --- |
| China | Qi M et al. | 2015 | chinchillas (*Chinchilla lanigera*) | 14 | 5 | *E. bieneusi* | - | 1 | [235] |
| China | Deng L et al. | 2017 | red-bellied tree squirrel (*Callosciurus erythraeus*) | 144 | 24 | *E. bieneusi* | 2014/3-2015/9 | 3 | [236] |
|  |  |  | deer mouse | 55 | 13 |  | 2014/3-2015/9 |  |  |
| China | Zhijun Z et al. | 2017 | red kangaroo | 23 | 0 | *E. bieneusi* | - | 3 | [237] |
|  |  |  | grey kangaroo | 38 | 14 | *E. bieneusi* | - |  |  |
| China | Deng L et al. | 2018 | chipmunk (*Eutamias asiaticus*) | 279 | 49 | *E. bieneusi* | 2016/3-2017/4 | 3 | [167] |
| China | [Zhao W et al.](https://www.ncbi.nlm.nih.gov/pubmed/?term=Zhao%20W%5BAuthor%5D&cauthor=true&cauthor_uid=29793513) | 2018 | brown rat (*Rattus norvegicus)* | 242 | 19 | *E. bieneusi* | 2014/4-2014/6 | 3 | [238] |
| Poland, Slovakia, The Czech Republic | Perec-Matysiak et al. | 2019 | *Apodemu sylvaticus* | 45 | 6 | *E. cuniculi* | 2011-2014 | 2 | [239] |
| China | Wang H et al. | 2019 | bamboo rat (*Rhizomys sinensis*) | 435 | 22 | *E. bieneusi* | 2017/2-2018/2 | 3 | [238] |
| Slovakia, The Czech Republic | Agnieszka Perec-Matysiak et al. | 2019 | *Myodes glareolus* | 465 | 70 | *E. cuniculi* | 2011-2014 | 3 | [240] |
| China | Deng L et al. | 2020 | red squirrel (*Sciurus vulgaris*) | 314 | 61 | *E. bieneusi* | - | 3 | [241] |
| China | Li J et al. | 2020 | rat | 291 | 14 | *E. bieneusi* | 2019/7-2019/8 | 3 | [242] |
| Poland | Perec-Matysiak A et al. |  | *Apodemus agrarius* | 184 | 79 | *E. bieneusi* | 2010-2012 | 3 | [243] |
|  |  |  | *Apodemus agrarius* | 60 | 18 |  |  |  |  |
|  |  |  | *Myodes glareolus* | 46 | 18 |  |  |  |  |
|  |  |  | *Mus musculus* | 21 | 6 |  |  |  |  |
| China | Bin-Ze Gui et al. | 2020 | rat | 228 | 76 | *E. bieneusi* | - | 3 | [244] |
| China | Yang Y et al. | 2015 | fox (*Vulpes vulpe*) | 191 | 44 | *E. bieneusi* | 2014/11 | 3 | [245] |
|  |  |  | farmed blue fox (*Alopex lagopu*) | 110 | 18 | *E. bieneusi* | 2014/6- 2015/3 | 3 | [246] |
|  |  |  | farmed blue fox (*Alopex lagopu*) | 110 | 18 |  |  |  |  |
|  |  |  | raccoon dog (*Nyctereutes procyonoide*) | 49 | 2 |  |  |  |  |
| China | Zhang XX et al. | 2016 | fox (*Vulpes lagopu*) | 302 | 37 | *E. bieneusi* | 2014 | 3 | [247] |
| Spain | Santín M et al. | 2017 | red fox (*Vulpes vulpe*) | 87 | 8 | *E. bieneusi* | 2003/12-2016/4 | 3 | [248] |
| China | Yuan-Yuan Ma et al. | 2020 | silver fox (*Vulpes vulpe*), arctic fox (*Vulpes lagopu*) | 344 | 31 | *E. bieneusi* | - | 3 | [249] |
| China | Karim MR et al. | 2014 | snack | 240 | 6 | *E. bieneusi* | - | 3 | [206] |
| USA | Guo Y et al. | 2014 | procyon lotor | 22 | 18 | *E. bieneusi* |  | 1 | [244] |
| China | Yang Y et al. | 2015 | fox (*Vulpes vulpes*) | 191 | 44 | *E. bieneusi* |  | 3 | [245] |
| China | Zhao W et al. | 2015 | blue fox (*Alopex lagopus*) | 110 | 18 | *E. bieneusi* | -- | 3 | [250] |
|  |  |  | raccoon dog (*Nyctereutes procyonoides*) | 49 | 2 |  |  |  |  |
| China | Zhao GH et al. | 2015 | takin | 191 | 28 | *E. bieneusi* | - | 4 | [244] |
| China | Yang Y et al. | 2015 | raccoon dog (*Nyctereutes procyonoides*) | 162 | 16 | *E. bieneusi* | - | 3 | [245] |
| China | Zhao W et al. | 2015 | raccoon dog (*Nyctereutes procyonoides*) | 49 | 2 | *E. bieneusi* | - | 3 | [251] |
| China | Xu C et al. | 2016 | raccoon dog (*Nyctereutes procyonoides*) | 305 | 68 | *E. bieneusi* | - | 4 | [252] |
| China | Yang Z et al. | 2016 | rabbit | 215 | 22 | *E. bieneusi* | - | 2 | [253] |
| China | Zhang XX et al. | 2016 | rabbit | 426 | 4 | *E. bieneusi* | - | 3 | [247] |
|  |  |  | fox (*Vulpes lagopus*) | 302 | 37 |  |  |  |  |
| Poland | Matysiak et al. | 2016 | raccoon | 49 | 2 | *E. bieneusi* | - |  | [254] |
| Spain | Santín M et al. | 2017 | red fox (*Vulpes vulpes*) | 87 | 8 | *E. bieneusi* | 2003/12-2016/4 | 3 | [255] |
|  |  | 2017 | european badger (*Meles meles*) | 69 | 16 |  |  |  |  |
| China | Li W et al. | 2018 | giant panda (*Ailuropoda melanoleuca*) | 200 | 69 | *E. bieneusi* | - | 3 | [256] |
| China | Wei Zhao et al. | 2018 | giant panda | 100 | 6 | *E. bieneusi* | 2013/9-2014/6 | 3 | [257] |
|  |  |  | red panda | 100 | 4 | *E. bieneusi* |  |  |  |
| China | Zhang XX et al. | 2018 | mink (*Neovison vison*) | 298 | 30 | *E. bieneusi* | - | 3 | [258] |
| China | Wu J et al. | 2018 | asiatic black bear (*Ursus thibetanus*) | 405 | 80 | *E. bieneusi* | - | 4 | [259] |
| Korea | Lee SH et al. | 2018 | bat | 268 | 7 | *E. bieneusi* | - | 3 | [260] |
| China | Cong W et al. | 2018 | mink (*Neovison vison*) | 559 | 23 | *E. bieneusi* | 2017 | 3 | [242] |
| China | Zhang Y et al. | 2018 | wombat | 315 | 1 | *E. bieneusi* | 2009/9-2017/3 | 3 | [243] |
|  |  |  | grey kangaroo | 647 | 13 | *E. bieneusi* |  |  |  |
|  |  |  | swamp wallaby | 403 | 5 | *E. bieneusi* |  |  |  |

-: Data not available.

**Table S8. Included studies of microsporidian infection in birds**

| **Author** | **Year** | **Country** | **Host** | **Feeding status** | **Life habit** | **No. of samples** | **No. of positive** | **Microsporidian species** | **Collection date** | **Quality score** | **Reference** |
| --- | --- | --- | --- | --- | --- | --- | --- | --- | --- | --- | --- |
| Barton et al. | 2003 | USA | birds | domestic | land | 198 | 49 | *E. hellem* | 1999 | 3 | [261] |
| Haro M et al. | 2005 | Spain | pigeon | wild | land | 124 | 18 | *E. bieneusi* | - | 4 | [262] |
|  |  |  |  |  |  |  | 5 | *E. intestinalis* |  |  |  |
|  |  |  |  |  |  |  | 1 | *E. hellem* |  |  |  |
|  |  |  |  |  |  |  | 1 | *E. bieneusi, E. intestinalis* |  |  |  |
|  |  |  |  |  |  |  | 1 | *E. intestinalis, E. hellem* |  |  |  |
| Slodkowicz-Kowalska A et al. | 2006 | Poland | bird | wild | amphibious | 570 | 20 | *E. hellem* | - | 3 | [263] |
|  |  |  | bird | wild | amphibious | 570 | 1 | *E. intestinalis* |  |  |  |
| Lobo ML et al. | 2006 | Portugal | bird | - | - | 83 | 24 | *E. bieneusi* | - | 3 | [264]) |
| Müller MG et al. | 2011 | United Arab Emirates | falcon | wild | land | 24 | 6 | *E. bieneusi* | - | 2 | [263] |
| Kašičková et al. | 2011 | Czech R. | bird | wild | land | 287 | 36 | *E. bieneusi* | - | 3 | [264] |
|  |  |  |  |  |  |  | 36 | *E. cuniculi* |  |  |  |
|  |  |  |  |  |  |  | 18 | *E. hellem* |  |  |  |
| Malcekova B et al. | 2012 | Slovakia | gyrfalcons | wild | land | 30 | 2 | *E. cuniculi* | - | 3 | [265, 266] |
| Lee SY et al. | 2015 | Korea | parrot | domestic | land | 51 | 7 | *E. hellem* | - | 3 | [267] |
|  |  |  |  |  |  |  | 1 | *E. cuniculi, E. hellem* |  |  |  |
| Lallo MA et al. | 2013 | Brazil | bird | wild | land | 196 | 11 | *E. bieneusi* | - | 4 | [268] |
|  |  |  |  |  |  |  | 32 | *E. hellem* |  |  |  |
|  |  |  |  |  |  |  | 3 | *E. intestinalis* |  |  |  |
|  |  |  |  |  |  |  | 2 | *E. cuniculi* |  |  |  |
| Słodkowicz-Kowalska A et al. | 2013 | Poland | pigeon | domestic | land | 139 | 5 | *E. hellem* | 2008-2010 | 2 | [269] |
|  |  |  |  |  |  |  | 4 | *E. intestinalis* |  |  |  |
|  |  |  |  |  |  |  | 1 | *E. cuniculi* |  |  |  |
|  |  |  |  |  |  |  | 1 | *E. bieneusi* |  |  |  |
| Malčeková B et al. | 2001 | Slovakia | bird | wild | amphibious | 47 | 19 | *E. cuniculi* | - | 3 | [270] |
| Pirestani M et al. | 2013 | Iran | pigeon | - | land | 147 | 4 | *E. intestinalis* | - | 3 | [271] |
|  |  |  |  | - | land | 147 | 13 | *E. bieneusi* |  |  |  |
|  |  |  |  | - | land | 147 | 2 | *E. cuniculi* |  |  |  |
|  |  |  |  | - | land | 147 | 6 | *E. hellem* |  |  |  |
| Snowden K et al. | 2014 | USA | hummingbird | wild | land | 100 | 19 | *E. hellem* | - | 3 | [270] |
| Li J et al. | 2015 | China | bird | - | - | 118 | 2 | *E. bieneusi* | 2013/2-3-2015/3 | 3 | [272] |
| Zhao W et al. | 2015 | China | duck | domestic | amphibious | 62 | 6 | *E. bieneusi* | 2013/7-2014/10 | 3 | [273] |
|  |  |  | goose | domestic | amphibious | 26 | 8 | *E. bieneusi* |  |  |  |
|  |  |  | pigeon | domestic | land | 50 | 22 | *E. bieneusi* |  |  |  |
| Vet Parasitol et al. | 2016 | Brazil | chicken | domestic | land | 151 | 24 | *E. bieneusi* | 2013/10- 2014/9 | 3 | [274] |
| Deng et al. et al. | 2017 | Brazil | bird | domestic | land | 85 | 3 | *E. bieneusi* | 2013/10-2014/9 | 3 | [275] |
| Rzymski P et al. | 2017 | Poland | cormorant | wild | amphibious | 25 | 0 | *Microsporidia* | 2013/6 | 3 | [276] |
| Deng L et al. | 2019 | China | bird | domestic | land | 387 | 97 | *E. bieneusi* | 2017/1-2018/8 |  | [277] |
| Wei Zhao et al. | 2019 | China | geese | domestic | amphibious | 142 | 10 | *E. bieneusi* | 2019/3-2019/7 | 3 | [278] |
|  |  |  | geese | wild | amphibious | 124 | 27 | *E. bieneusi* | - |  |  |
| Tavalla M et al. | 2018 | Iran | bird | wild | land | 816 | 103 | *E. bieneusi* | 2012-2014 | 4 | [279] |
|  |  |  |  |  |  |  | 52 | *E. intestinalis* |  |  |  |
|  |  |  |  |  |  |  | 9 | *E. hellem* |  |  |  |
|  |  |  |  |  |  |  | 17 | *E. cuniculi* |  |  |  |
| Feng SY et al. | 2012 | China | peafowl (*Pavo cristatus*) | domestic | land | 258 | 17 | *E. bieneusi* | 2017/11-2018/6 | 3 | [280] |

-: Data not available

**Table S9. Included studies of microsporidia in water**

| **Country** | **Author** | **Year** | **Origin** | **No. of sample** | **No. of positive** | **Microsporidian species** | **Quality score** | **Reference** |
| --- | --- | --- | --- | --- | --- | --- | --- | --- |
| Tunisia | Galvan et al. | 2013 | drinking water treatment plant | 232 | 143 | *E. bieneusi* | 3 | [281] |
| Spain | Guo et al. | 2014 | wastewater treatment plant | 112 | 64 | Microsporidia | 4 | [206] |
|  |  |  | drinking water treatment plant | 63 | 17 |  |  |  |
|  |  |  | unknow | 43 | 28 |  |  |  |
| China | Zhao W et al. | 2016 | lake water | 23 | 13 | *E. bieneusi* | 2 | [282] |
| China | Ma J et al. | 2016 | wastewater treatment plant-treated water | 50 | 35 | *E. bieneusi* | 2 | [283] |
| Spain | Ye et al. | 2017 | waste-water | 386 | 338 | *E. bieneusi* | 4 | [284] |
| USA | Huang et al. | 2017 | stormwater | 67 | 39 | *E. bieneusi* | 2 | [285] |
| China | Yamashiro et al. | 2017 | wastewater treatment plant-raw water | 108 | 46 | *E. bieneusi* | 3 | [286] |
| China | MossJA et al. | 2017 | combined sewer overflow | 40 | 40 | *E. bieneusi* | 2 | [287] |
| USA | Moss JA et al. | 2017 | water | 14 | 7 | *E. bieneusi* | 1 | [287] |
| Brazil | Chen et al. | 2018 | wastewater treatment plant-raw water/-treated water | 18 | 5 | *E. bieneusi* | 2 | [288] |
| China | Chen JS et al. | 2018 | drinking water | 50 | 48 | *Vittaforma* | 2 | [288] |

-: Data not available.

**Table S10.** **Checklist of items included when reporting a meta-analysis**

| **Section/topic** | **#** | **Checklist item** | **Reported in page #** |
| --- | --- | --- | --- |
| TITLE | | | |
| Title | 1 | Identify the report as a systematic review, meta-analysis, or both. | 1 |
| ABSTRACT | | | |
| Structured summary | 2 | Provide a structured summary including, as applicable: background; objectives; data sources; study eligibility criteria, participants, and interventions; study appraisal and synthesis methods; results; limitations; conclusions and implications of key findings; systematic review registration number. | 2-3 |
| INTRODUCTION | | | |
| Rationale | 3 | Describe the rationale for the review in the context of what is already known. | 4-5 |
| Objectives | 4 | Provide an explicit statement of questions being addressed with reference to participants, interventions, comparisons, outcomes, and study design (PICOS). | 5 |
| METHODS | | | |
| Protocol and registration | 5 | Indicate if a review protocol exists, if and where it can be accessed (e.g., Web address), and, if available, provide registration information including registration number. | N/A |
| Eligibility criteria | 6 | Specify study characteristics (e.g., PICOS, length of follow-up) and report characteristics (e.g., years considered, language, publication status) used as criteria for eligibility, giving rationale. | 5 |
| Information sources | 7 | Describe all information sources (e.g., databases with dates of coverage, contact with study authors to identify additional studies) in the search and date last searched. | 5-6 |
| Search | 8 | Present full electronic search strategy for at least one database, including any limits used, such that it could be repeated. | 5-6 |
| Study selection | 9 | State the process for selecting studies (i.e., screening, eligibility, included in systematic review, and, if applicable, included in the meta-analysis). | 6, Figure 1 |
| Data collection process | 10 | Describe method of data extraction from reports (e.g., piloted forms, independently, in duplicate) and any processes for obtaining and confirming data from investigators. | 6 |
| Data items | 11 | List and define all variables for which data were sought (e.g., PICOS, funding sources) and any assumptions and simplifications made. | 6 |
| Risk of bias in individual studies | 12 | Describe methods used for assessing risk of bias of individual studies (including specification of whether this was done at the study or outcome level), and how this information is to be used in any data synthesis. | 7 |
| Summary measures | 13 | State the principal summary measures (e.g., risk ratio, difference in means). | 7 |
| Synthesis of results | 14 | Describe the methods of handling data and combining results of studies, if done, including measures of consistency (e.g., I2) for each meta-analysis. | 7 |
| Risk of bias across studies | 15 | Specify any assessment of risk of bias that may affect the cumulative evidence (e.g., publication bias, selective reporting within studies). | 7 |
| Additional analyses | 16 | Describe methods of additional analyses (e.g., sensitivity or subgroup analyses, meta-regression), if done, indicating which were pre-specified. | 7 |
| RESULTS | | | |
| Study selection | 17 | Give numbers of studies screened, assessed for eligibility, and included in the review, with reasons for exclusions at each stage, ideally with a flow diagram. | 6, Figure 1 |
| Study characteristics | 18 | For each study, present characteristics for which data were extracted (e.g., study size, PICOS, follow-up period) and provide the citations. | Additional file 1: Tables S1-S9 |
| Risk of bias within studies | 19 | Present data on risk of bias of each study and, if available, any outcome-level assessment (see Item 12). | Additional file 1: Tables S1-9 |
| Results of individual studies | 20 | For all outcomes considered (benefits or harms), present, for each study: (a) simple summary data for each intervention group and (b) effect estimates and confidence intervals, ideally with a forest plot. | Additional file 1: Figures S1-S26, |
| Synthesis of results | 21 | Present results of each meta-analysis done, including confidence intervals and measures of consistency. | 8-13, Additional file 1: Figures S1-26 |
| Risk of bias across studies | 22 | Present results of any assessment of risk of bias across studies (see Item 15). | N/A |
| Additional analysis | 23 | Give results of additional analyses, if done (e.g., sensitivity or subgroup analyses, meta-regression [see Item 16]). | 8-13, Table 1, Table 2 |
| DISCUSSION | | | |
| Summary of evidence | 24 | Summarize the main findings including the strength of evidence for each main outcome; consider their relevance to key groups (e.g., health care providers, users, and policy makers). | 8-10 |
| Limitations | 25 | Discuss limitations at study and outcome level (e.g., risk of bias), and at review level (e.g., incomplete retrieval of identified research, reporting bias). | 14-15 |
| Conclusions | 26 | Provide a general interpretation of the results in the context of other evidence, and implications for future research. | 15 |
| FUNDING | | | |
| Funding | 27 | Describe sources of funding for the systematic review and other support (e.g., supply of data); role of funders for the systematic review. | 17 |

**REFERENCES**

1. Weber, R.; Bryan, R. T.; Owen, R. L.; Wilcox, C. M.; Gorelkin, L.; Visvesvara, G. S., Improved light-microscopical detection of microsporidia spores in stool and duodenal aspirates. The Enteric Opportunistic Infections Working Group. The New England journal of medicine 1992, 326, (3), 161-6.

2. Kotler, D. P.; Orenstein, J. M., Prevalence of intestinal microsporidiosis in HIV-infected individuals referred for gastroenterological evaluation. The American journal of gastroenterology 1994, 89, (11), 1998-2002.

3. van Gool, T.; Luderhoff, E.; Nathoo, K. J.; Kiire, C. F.; Dankert, J.; Mason, P. R., High prevalence of *Enterocytozoon bieneusi* infections among HIV-positive individuals with persistent diarrhoea in Harare, Zimbabwe. Transactions of the Royal Society of Tropical Medicine and Hygiene 1995, 89, (5), 478-80.

4. Anwar-Bruni, D. M.; Hogan, S. E.; Schwartz, D. A.; Wilcox, C. M.; Bryan, R. T.; Lennox, J. L., Atovaquone is effective treatment for the symptoms of gastrointestinal microsporidiosis in HIV-1-infected patients. Aids 1996, 10, (6), 619-23.

5. Coyle, C. M.; Wittner, M.; Kotler, D. P.; Noyer, C.; Orenstein, J. M.; Tanowitz, H. B.; Weiss, L. M., Prevalence of microsporidiosis due to *Enterocytozoon bieneusi* and Encephalitozoon (Septata) intestinalis among patients with AIDS-related diarrhea: determination by polymerase chain reaction to the microsporidian small-subunit rRNA gene. Clinical infectious diseases : an official publication of the Infectious Diseases Society of America 1996, 23, (5), 1002-6.

6. Mwachari, C.; Batchelor, B. I. F.; Paul, J.; Waiyaki, P. G.; Gilks, C. F., Chronic diarrhoea among HIV-infected adult patients in Nairobi, Kenya. Journal of Infection 1998, 37, (1), 48-53.

7. Cegielski, J. P.; Ortega, Y. R.; McKee, S.; Madden, J. F.; Gaido, L.; Schwartz, D. A.; Manji, K.; Jorgensen, A. F.; Miller, S. E.; Pulipaka, U. P.; Msengi, A. E.; Mwakyusa, D. H.; Sterling, C. R.; Reller, L. B., Cryptosporidium, enterocytozoon, and cyclospora infections in pediatric and adult patients with diarrhea in Tanzania. Clinical infectious diseases : an official publication of the Infectious Diseases Society of America 1999, 28, (2), 314-21.

8. Punpoowong, B.; Viriyavejakul, P.; Riganti, M.; Pongponaratn, E.; Chaisri, U.; Maneerat, Y., Opportunistic protozoa in stool samples from HIV-infected patients. The Southeast Asian journal of tropical medicine and public health 1998, 29, (1), 31-4.

9. Wanachiwanawin, D.; Manatsathit, S.; Lertlaituan, P.; Thakerngpol, K.; Suwanagool, P., Intestinal microsporidiosis in HIV infected patients with chronic diarrhea in Thailand. The Southeast Asian journal of tropical medicine and public health 1998, 29, (4), 767-71.

10. Chokephaibulkit, K.; Wanachiwanawin, D.; Tosasuk, K.; Pavitpok, J.; Vanprapar, N.; Chearskul, S., Intestinal parasitic infections among human immunodeficiency virus-infected and -uninfected children hospitalized with diarrhea in Bangkok, Thailand. The Southeast Asian journal of tropical medicine and public health 2001, 32, (4), 770-5.

11. Gumbo, T.; Sarbah, S.; Gangaidzo, I. T.; Ortega, Y.; Sterling, C. R.; Carville, A.; Tzipori, S.; Wiest, P. M., Intestinal parasites in patients with diarrhea and human immunodeficiency virus infection in Zimbabwe. Aids 1999, 13, (7), 819-21.

12. Brandonisio, O.; Maggi, P.; Panaro, M. A.; Lisi, S.; Andriola, A.; Acquafredda, A.; Angarano, G., Intestinal protozoa in HIV-infected patients in Apulia, South Italy. Epidemiology and infection 1999, 123, (3), 457-62.

13. Lebbad, M.; Norrgren, H.; Nauclér, A.; Dias, F.; Andersson, S.; Linder, E., Intestinal parasites in HIV-2 associated AIDS cases with chronic diarrhoea in Guinea-Bissau. Acta Trop 2001, 80, (1), 45-9.

14. Wiwanitkit, V., Intestinal parasitic infections in Thai HIV-infected patients with different immunity status. BMC gastroenterology 2001, 1, 3-3.

15. Waywa, D.; Kongkriengdaj, S.; Chaidatch, S.; Tiengrim, S.; Kowadisaiburana, B.; Chaikachonpat, S.; Suwanagool, S.; Chaiprasert, A.; Curry, A.; Bailey, W.; Suputtamongkol, Y.; Beeching, N. J., Protozoan enteric infection in AIDS related diarrhea in Thailand. The Southeast Asian journal of tropical medicine and public health 2001, 32 Suppl 2, 151-5.

16. Sadler, F.; Peake, N.; Borrow, R.; Rowl, P. L.; Wilkins, E. G. L.; Curry, A., Genotyping of *Enterocytozoon bieneusi* in AIDS Patients from the North West of England. Journal of Infection 2002, 44, (1), 39-42.

17. Wanachiwanawin, D.; Chokephaibulkit, K.; Lertlaituan, P.; Ongrotchanakun, J.; Chinabut, P.; Thakerngpol, K., Intestinal microsporidiosis in HIV-infected children with diarrhea. The Southeast Asian journal of tropical medicine and public health 2002, 33, (2), 241-5.

18. Kumar, S. S.; Ananthan, S.; Lakshmi, P., Intestinal parasitic infection in HIV infected patients with diarrhoea in Chennai. Indian journal of medical microbiology 2002, 20, (2), 88-91.

19. Mohandas, K.; Sehgal, R.; Sud, A.; Malla, N., Prevalence of intestinal parasitic pathogens in HIV-seropositive individuals in Northern India. Japanese journal of infectious diseases 2002, 55, (3), 83-84.

20. Florez, A. C.; Garcia, D. A.; Moncada, L.; Beltran, M., Prevalence of microsporidia and other intestinal parasites in patients with HIV infection, Bogota, 2001. Biomedica : revista del Instituto Nacional de Salud 2003, 23, (3), 274-82.

21. Endeshaw, T.; Kebede, A.; Verweij, J. J.; Wolday, D.; Zewide, A.; Tsige, K.; Abraham, Y.; Messele, T.; Polderman, A. M.; Petros, B., Detection of intestinal microsporidiosis in diarrhoeal patients infected with the human immunideficiency virus (HIV-1) using PCR and uvitex-2B stain. Ethiopian Med J 2005, 43, (2), 97-101.

22. Bern, C.; Kawai, V.; Vargas, D.; Rabke-Verani, J.; Williamson, J.; Chavez-Valdez, R.; Xiao, L. H.; Sulaiman, I.; Vivar, A.; Ticona, E.; Navincopa, M.; Cama, V.; Moura, H.; Secor, W. E.; Visvesvara, G.; Gilman, R. H., The epidemiology of intestinal microsporidiosis in patients with HIV/AIDS in Lima, Peru. Journal of Infectious Diseases 2005, 191, (10), 1658-1664.

23. Tumwine, J. K.; Kekitiinwa, A.; Bakeera-Kitaka, S.; Ndeezi, G.; Downing, R.; Feng, X. C.; Akiyoshi, D. E.; Tzipori, S., Cryptosporidiosis and microsporidiosis in Ugandan children with persistent diarrhea with and without concurrent infection with the human immunodeficiency virus. Am J Trop Med Hyg 2005, 73, (5), 921-925.

24. Endeshaw, T.; Kebede, A.; Verweij, J. J.; Zewide, A.; Tsige, K.; Abraham, Y.; Wolday, D.; Woldemichael, T.; Messele, T.; Polderman, A. M.; Petros, B., Intestinal microsporidiosis in diarrheal patients infected with human immunodeficiency virus-1 in Addis Ababa, Ethiopia. Japanese journal of infectious diseases 2006, 59, (5), 306-10.

25. Chacin-Bonilla, L.; Panunzio, A. P.; Monsalve-Castillo, F. M.; Parra-Cepeda, I. E.; Martinez, R., Microsporidiosis in Venezuela: prevalence of intestinal microsporidiosis and its contribution to diarrhea in a group of human immunodeficiency virus-infected patients from Zulia State. Am J Trop Med Hyg 2006, 74, (3), 482-6.

26. Sarfati, C.; Bourgeois, A.; Menotti, J.; Liegeois, F.; Moyou-Somo, R.; Delaporte, E.; Derouin, F.; Ngole, E. M.; Molina, J. M., Prevalence of intestinal parasites including microsporidia in human immunodeficiency virus-infected adults in Cameroon: A cross-sectional study. Am J Trop Med Hyg 2006, 74, (1), 162-164.

27. Espern, A.; Morio, F.; Miegeville, M.; Illa, H.; Abdoulaye, M.; Meyssonnier, V.; Adehossi, E.; Lejeune, A.; Cam, P. D.; Besse, B.; Gay-Andrieu, F., Molecular study of microsporidiosis due to Enterocytozoon bieneusi and Encephalitozoon intestinalis among human immunodeficiency virus-infected patients from two geographical areas: Niamey, Niger, and Hanoi, Vietnam. Journal of clinical microbiology 2007, 45, (9), 2999-3002.

28. Breton, J.; Bart-Delabesse, E.; Biligui, S.; Carbone, A.; Seiller, X.; Okome-Nkoumou, M.; Nzamba, C.; Kombila, M.; Accoceberry, I.; Thellier, M., New highly divergent rRNA sequence among biodiverse genotypes of *Enterocytozoon bieneusi* strains isolated from humans in Gabon and Cameroon. J Clin Microbiol 2007, 45, (8), 2580-9.

29. Breton, J.; Bart-Delabesse, E.; Biligui, S.; Carbone, A.; Seiller, X.; Okome-Nkoumou, M.; Nzamba, C.; Kombila, M.; Accoceberry, I.; Thellier, M., New highly divergent rRNA sequence among biodiverse genotypes of *Enterocytozoon bieneusi* strains isolated from humans in Gabon and Cameroon. Journal of Clinical Microbiology 2007, 45, (8), 2580-2589.

30. Espern, A.; Morio, F.; Miegeville, M.; Illa, H.; Abdoulaye, M.; Meyssonnier, V.; Adehossi, E.; Lejeune, A.; Cam, P. D.; Besse, B.; Gay-Andrieu, F., Molecular study of microsporidiosis due to *Enterocytozoon bieneusi* and Encephalitozoon intestinalis among human immunodeficiency virus-infected patients from two geographical areas: Niamey, Niger, and Hanoi, Vietnam. Journal of clinical microbiology 2007, 45, (9), 2999-3002.

31. Dwivedi, K. K.; Prasad, G.; Saini, S.; Mahajan, S.; Lal, S.; Baveja, U. K., Enteric opportunistic parasites among HIV infected individuals: Associated risk factors and immune status. Japanese journal of infectious diseases 2007, 60, (2-3), 76-81.

32. Stark, D.; van Hal, S.; Barratt, J.; Ellis, J.; Marriott, D.; Harkness, J., Limited genetic diversity among genotypes of *Enterocytozoon bieneusi* strains Isolated from HIV-Infected patients from Sydney, Australia. J Med Microbiol 2009, 58, (3), 355-357.

33. Lagrange-Xélot, M.; Porcher, R.; Sarfati, C.; de Castro, N.; Carel, O.; Magnier, J. D.; Delcey, V.; Molina, J. M., Isosporiasis in patients with HIV infection in the highly active antiretroviral therapy era in France. HIV medicine 2008, 9, (2), 126-30.

34. ten Hove, R. J.; Van Lieshout, L.; Beadsworth, M. B.; Perez, M. A.; Spee, K.; Claas, E. C.; Verweij, J. J., Characterization of genotypes of *Enterocytozoon bieneusi* in immunosuppressed and immunocompetent patient groups. J Eukaryot Microbiol 2009, 56, (4), 388-93.

35. Pagornrat, W.; Leelayoova, S.; Rangsin, R.; Tan-Ariya, P.; Naaglor, T.; Mungthin, M., Carriage Rate of *Enterocytozoon bieneusi* in an Orphanage in Bangkok, Thailand. Journal of Clinical Microbiology 2009, 47, (11), 3739-3741.

36. Saksirisampant, W.; Prownebon, J.; Saksirisampant, P.; Mungthin, M.; Siripatanapipong, S.; Leelayoova, S., Intestinal parasitic infections: prevalences in HIV/AIDS patients in a Thai AIDS-care centre. Ann Trop Med Parasit 2009, 103, (7), 573-581.

37. Viriyavejakul, P.; Nintasen, R.; Punsawad, C.; Chaisri, U.; Punpoowong, B.; Riganti, M., High prevalence of Microsporidium infection in HIV-infected patients. The Southeast Asian journal of tropical medicine and public health 2009, 40, (2), 223-8.

38. Kulkarni, S. V.; Kairon, R.; Sane, S. S.; Padmawar, P. S.; Kale, V. A.; Thakar, M. R.; Mehendale, S. M.; Risbud, A. R., Opportunistic parasitic infections in HIV/AIDS patients presenting with diarrhoea by the level of immunesuppression. Indian J Med Res 2009, 130, (1), 63-66.

39. Wumba, R.; Longo-Mbenza, B.; Mandina, M.; Odio, W. T.; Biligui, S.; Sala, J.; Breton, J.; Thellier, M., Intestinal parasites infections in hospitalized AIDS patients in Kinshasa, Democratic Republic of Congo. Parasite 2010, 17, (4), 321-8.

40. Sokolova, O. I.; Demyanov, A. V.; Bowers, L. C.; Didier, E. S.; Yakovlev, A. V.; Skarlato, S. O.; Sokolova, Y. Y., Emerging Microsporidian Infections in Russian HIV-Infected Patients. Journal of Clinical Microbiology 2011, 49, (6), 2102-2108.

41. Akinbo, F. O.; Okaka, C. E.; Omoregie, R.; Dearen, T.; Leon, E. T.; Xiao, L., Molecular epidemiologic characterization of *Enterocytozoon bieneusi* in HIV-infected persons in Benin City, Nigeria. Am J Trop Med Hyg 2012, 86, (3), 441-5.

42. Maikai, B. V.; Umoh, J. U.; Lawal, I. A.; Kudi, A. C.; Ejembi, C. L.; Xiao, L., Molecular characterizations of Cryptosporidium, Giardia, and Enterocytozoon in humans in Kaduna State, Nigeria. Experimental parasitology 2012, 131, (4), 452-6.

43. Ojuromi, O. T.; Izquierdo, F.; Fenoy, S.; Fagbenro-Beyioku, A.; Oyibo, W.; Akanmu, A.; Odunukwe, N.; Henriques-Gil, N.; del Aguila, C., Identification and characterization of microsporidia from fecal samples of HIV-positive patients from Lagos, Nigeria. PloS one 2012, 7, (4), e35239.

44. Wumba, R.; Jean, M.; Benjamin, L.-M.; Madone, M.; Fabien, K.; Josue, Z.; Jean, S.; Eric, K.; A C, G.-O.; Marc, T., *Enterocytozoon bieneusi* Identification Using Real-Time Polymerase Chain Reaction and Restriction Fragment Length Polymorphism in HIV-Infected Humans from Kinshasa Province of the Democratic Republic of Congo. Journal of parasitology research 2012, 2012, 278028-278028.

45. Lobo, M. L.; Xiao, L.; Antunes, F.; Matos, O., Microsporidia as emerging pathogens and the implication for public health: A 10-year study on HIV-positive and -negative patients. International Journal for Parasitology 2012, 42, (2), 197-205.

46. Chabchoub, N.; Abdelmalek, R.; Breton, J.; Kanoun, F.; Thellier, M.; Bouratbine, A.; Aoun, K., Genotype identification of *Enterocytozoon bieneusi* isolates from stool samples of HIV-infected Tunisian patients. Parasite 2012, 19, (2), 147-51.

47. Wang, L.; Zhang, H.; Zhao, X.; Zhang, L.; Zhang, G.; Guo, M.; Liu, L.; Feng, Y.; Xiao, L., Zoonotic Cryptosporidium Species and *Enterocytozoon bieneusi* Genotypes in HIV-Positive Patients on Antiretroviral Therapy. Journal of Clinical Microbiology 2013, 51, (2), 557-563.

48. Rivero-Rodriguez, Z.; Hernandez, A.; Bracho, A.; Salazar, S.; Villalobos, R., Prevalence of intestinal microsporidia and other intestinal parasites in hiv positive patients from Maracaibo, Venezuela. Biomedica : revista del Instituto Nacional de Salud 2013, 33, (4), 538-545.

49. Agholi, M.; Hatam, G. R.; Motazedian, M. H., HIV/AIDS-associated opportunistic protozoal diarrhea. AIDS Res Hum Retroviruses 2013, 29, (1), 35-41.

50. Akinbo, F. O.; Okaka, C. E.; Omoregie, R.; Adamu, H.; Xiao, L., Unusual *Enterocytozoon bieneusi* genotypes and Cryptosporidium hominis subtypes in HIV-infected patients on highly active antiretroviral therapy. Am J Trop Med Hyg 2013, 89, (1), 157-61.

51. Ayinmode, A. B.; Zhang, H.; Dada-Adegbola, H. O.; Xiao, L., Cryptosporidium hominis subtypes and *Enterocytozoon bieneusi* genotypes in HIV-infected persons in Ibadan, Nigeria. Zoonoses and public health 2014, 61, (4), 297-303.

52. Mirjalali, H.; Mohebali, M.; Mirhendi, H.; Gholami, R.; Keshavarz, H.; Meamar, A. R.; Rezaeian, M., Emerging Intestinal Microsporidia Infection in HIV+/AIDS Patients in Iran: Microscopic and Molecular Detection. Iran J Parasitol 2014, 9, (2), 149-154.

53. Ojuromi, O. T.; Duan, L.; Izquierdo, F.; Fenoy, S. M.; Oyibo, W. A.; Del Aguila, C.; Ashafa, A. O.; Feng, Y.; Xiao, L., Genotypes of Cryptosporidium spp. and *Enterocytozoon bieneusi* in Human Immunodeficiency Virus-Infected Patients in Lagos, Nigeria. The Journal of eukaryotic microbiology 2016, 63, (4), 414-8.

54. Khalil, S.; Mirdha, B. R.; Sinha, S.; Panda, A.; Singh, Y.; Joseph, A.; Deb, M., Intestinal Parasitosis in Relation to Anti-Retroviral Therapy, CD4(+) T-cell Count and Diarrhea in HIV Patients. The Korean journal of parasitology 2015, 53, (6), 705-712.

55. Mirjalali, H.; Mirhendi, H.; Meamar, A. R.; Mohebali, M.; Askari, Z.; Mirsamadi, E. S.; Rezaeian, M., Genotyping and molecular analysis of *Enterocytozoon bieneusi* isolated from immunocompromised patients in Iran. Infect Genet Evol 2015, 36, 244-249.

56. Nsagha, D. S.; Njunda, A. L.; Assob, N. J. C.; Ayima, C. W.; Tanue, E. A.; Kibu, O. D.; Kwenti, T. E., Intestinal parasitic infections in relation to CD4(+) T cell counts and diarrhea in HIV/AIDS patients with or without antiretroviral therapy in Cameroon. Bmc Infect Dis 2016, 16, 9-9.

57. Mitra, S.; Mukherjee, A.; Khanra, D.; Bhowmik, A.; Roy, K.; Talukdar, A., Enteric Parasitic Infection Among Antiretroviral Therapy Naive HIV-Seropositive People: Infection Begets Infection-Experience from Eastern India. J Glob Infect Dis 2016, 8, (2), 82-86.

58. Liu, H.; Jiang, Z.; Yuan, Z.; Yin, J.; Wang, Z.; Yu, B.; Zhou, D.; Shen, Y.; Cao, J., Infection by and genotype characteristics of *Enterocytozoon bieneusi* in HIV/AIDS patients from Guangxi Zhuang autonomous region, China. Bmc Infect Dis 2017, 17.

59. Khanduja, S.; Ghoshal, U.; Agarwal, V.; Pant, P.; Ghoshal, U. C., Identification and genotyping of *Enterocytozoon bieneusi* among human immunodeficiency virus infected patients. J Infect Public Heal 2017, 10, (1), 31-40.

60. Tavalla, M.; Mardani-Kateki, M.; Abdizadeh, R.; Nashibi, R.; Rafie, A.; Khademvatan, S., Molecular identification of *Enterocytozoon bieneusi* and Encephalitozoon spp. in immunodeficient patients in Ahvaz, Southwest of Iran. Acta tropica 2017, 172, 107-112.

61. Wan Sulaiman, W.; Lim, Y.; Hassan, N.-A.; Mohd Shaharuddin, N.; Mahmud, R.; Ngui, R., Molecular Diagnosis of Microsporidia among Immunocompromised Patients in Kuala Lumpur, Malaysia. The American Journal of Tropical Medicine and Hygiene 2018, 99.

62. Hassan, N.-A.; Lim, Y. A. L.; Mahmud, R.; Mohd-Shaharuddin, N.; Sulaiman, W. Y. W.; Ngui, R., Molecular Diagnosis of Microsporidia among Immunocompromised Patients in Kuala Lumpur, Malaysia. Am J Trop Med Hyg 2018, 99, (6), 1562-1566.

63. MATHEWSON, J. J., <HEp-2 Cell-Adherent Escherichia coli and Intestinal Secretory Immune Response to Human Immunodeﬁciency Virus (HIV) in Outpatients with HIV-Associated Diarrhe.pdf>. 1998.

64. Tumwine, J. K.; Kekitiinwa, A.; Nabukeera, N.; Akiyoshi, D. E.; Buckholt, M. A.; Tzipori, S., *Enterocytozoon bieneusi* among children with diarrhea attending Mulago Hospital in Uganda. The American journal of tropical medicine and hygiene 2002, 67, (3), 299-303.

65. Lores, B.; López-Miragaya, I.; Arias, C.; Fenoy, S.; Torres, J.; del Aguila, C., Intestinal microsporidiosis due to *Enterocytozoon bieneusi* in elderly human immunodeficiency virus--negative patients from Vigo, Spain. Clinical infectious diseases : an official publication of the Infectious Diseases Society of America 2002, 34, (7), 918-21.

66. Wang, T.; Fan, Y.; Koehler, A. V.; Ma, G.; Li, T.; Hu, M.; Gasser, R. B., First survey of Cryptosporidium, Giardia and Enterocytozoon in diarrhoeic children from Wuhan, China. Infect Genet Evol 2017, 51, 127-131.

67. Zhang, Y.; Koehler, A. V.; Wang, T.; Robertson, G. J.; Bradbury, R. S.; Gasser, R. B., *Enterocytozoon bieneusi* genotypes in people with gastrointestinal disorders in Queensland and Western Australia. Infection, genetics and evolution : journal of molecular epidemiology and evolutionary genetics in infectious diseases 2018, 65, 293-299.

68. Ding, S.; Huang, W.; Qin, Q.; Tang, J.; Liu, H., Genotype Identification and Phylogenetic Analysis of *Enterocytozoon bieneusi* Isolates from Stool Samples of Diarrheic Children. (1937-2345 (Electronic)).

69. Huibers, M. H. W.; Moons, P.; Maseko, N.; Gushu, M. B.; Iwajomo, O. H.; Heyderman, R. S.; van Hensbroek, M. B.; Brienen, E. A.; van Lieshout, L.; Calis, J. C. J., Multiplex Real-time PCR Detection of Intestinal Protozoa in HIV-infected Children in Malawi: *Enterocytozoon Bieneusi* Is Common and Associated With Gastrointestinal Complaints and May Delay BMI (Nutritional Status) Recovery. Pediatr Infect Dis J 2018, 37, (9), 910-915.

70. Oguz Kaya, I.; Dogruman Al, F.; Mumcuoglu, I., Investigation of Microsporidia Prevalence with Calcofluor White and Uvitex 2B Chemiluminescence Staining Methods and Molecular Analysis of Species in Diarrheal Patients. Mikrobiyoloji bulteni 2018, 52, (4), 401-412.

71. Ding, S.; Huang, W.; Qin, Q.; Tang, J.; Liu, H., Genotype Identification and Phylogenetic Analysis of *Enterocytozoon bieneusi* Isolates from Stool Samples of Diarrheic Children. The Journal of parasitology 2018, 104, (3), 297-301.

72. Tumwine, J. K.; Kekitiinwa, A.; Nabukeera, N.; Akiyoshi, D. E.; Buckholt, M. A.; Tzipori, S., *Enterocytozoon bieneusi* among children with diarrhea attending Mulago Hospital in Uganda. Am J Trop Med Hyg 2002, 67, (3), 299-303.

73. Leelayoova, S.; Subrungruang, I.; Rangsin, R.; Chavalitshewinkoon-Petmitr, P.; Worapong, J.; Naaglor, T.; Mungthin, M., Transmission of *Enterocytozoon bieneusi* genotype a in a Thai orphanage. Am J Trop Med Hyg 2005, 73, (1), 104-7.

74. Samie, A.; Obi, C. L.; Tzipori, S.; Weiss, L. M.; Guerrant, R. L., Microsporidiosis in South Africa: PCR detection in stool samples of HIV-positive and HIV-negative individuals and school children in Vhembe district, Limpopo Province. Transactions of the Royal Society of Tropical Medicine and Hygiene 2007, 101, (6), 547-554.

75. Decraene, V.; Lebbad, M.; Botero-Kleiven, S.; Gustavsson, A. M.; Lofdahl, M., First reported foodborne outbreak associated with microsporidia, Sweden, October 2009. Epidemiol Infect 2012, 140, (3), 519-527.

76. Leelayoova, S.; Piyaraj, P.; Subrungruang, I.; Pagornrat, W.; Naaglor, T.; Phumklan, S.; Taamasri, P.; Suwanasri, J.; Mungthin, M., Genotypic Characterization of *Enterocytozoon bieneusi* in Specimens from Pigs and Humans in a Pig Farm Community in Central Thailand. Journal of Clinical Microbiology 2009, 47, (5), 1572-1574.

77. Pagornrat, W.; Leelayoova, S.; Rangsin, R.; Tan-Ariya, P.; Naaglor, T.; Mungthin, M., Carriage rate of *Enterocytozoon bieneusi* in an orphanage in Bangkok, Thailand. J Clin Microbiol 2009, 47, (11), 3739-41.

78. Zhang, X.; Wang, Z.; Su, Y.; Liang, X.; Sun, X.; Peng, S.; Lu, H.; Jiang, N.; Yin, J.; Xiang, M.; Chen, Q., Identification and genotyping of *Enterocytozoon bieneusi* in China. Journal of clinical microbiology 2011, 49, (5), 2006-8.

79. Sak, B.; Brady, D.; Pelikanova, M.; Kvetonova, D.; Rost, M.; Kostka, M.; Tolarova, V.; Huzova, Z.; Kvac, M., Unapparent Microsporidial Infection among Immunocompetent Humans in the Czech Republic. Journal of Clinical Microbiology 2011, 49, (3), 1064-1070.

80. Dengjel, B.; Zahler, M.; Hermanns, W.; Heinritzi, K.; Spillmann, T.; Thomschke, A.; Loscher, T.; Gothe, R.; Rinder, H., Zoonotic potential of *Enterocytozoon bieneusi*. Journal of Clinical Microbiology 2001, 39, (12), 4495-4499.

81. Lobo, M. L.; Xiao, L.; Antunes, F.; Matos, O., Microsporidia as emerging pathogens and the implication for public health: a 10-year study on HIV-positive and -negative patients. International journal for parasitology 2012, 42, (2), 197-205.

82. Mori, H.; Mahittikorn, A.; Watthanakulpanich, D.; Komalamisra, C.; Sukthana, Y., Zoonotic potential of *Enterocytozoon bieneusi* among children in rural communities in Thailand. Parasite 2013, 20.

83. Lobo, M. L.; Augusto, J.; Antunes, F.; Ceita, J.; Xiao, L.; Codices, V.; Matos, O., Cryptosporidium spp., Giardia duodenalis, *Enterocytozoon bieneusi* and other intestinal parasites in young children in Lobata province, Democratic Republic of São Tomé and Principe. PLoS One 2014, 9, (5), e97708.

84. Yang, J. P.; Song, M. X.; Wan, Q.; Li, Y. J.; Lu, Y. X.; Jiang, Y. X.; Tao, W.; Li, W., *Enterocytozoon bieneusi* Genotypes in Children in Northeast China and Assessment of Risk of Zoonotic Transmission. Journal of Clinical Microbiology 2014, 52, (12), 4363-4367.

85. Ndzi, E. S.; Asonganyi, T.; Nkinin, M. B.; Xiao, L.; Didier, E. S.; Bowers, L. C.; Nkinin, S. W.; Kaneshiro, E. S., Fast Technology Analysis Enables Identification of Species and Genotypes of Latent Microsporidia Infections in Healthy Native Cameroonians. The Journal of eukaryotic microbiology 2016, 63, (2), 146-52.

86. Prasertbun, R.; Mori, H.; Pintong, A.-r.; Sanyanusin, S.; Popruk, S.; Komalamisra, C.; Changbunjong, T.; Buddhirongawatr, R.; Sukthana, Y.; Mahittikorn, A., Zoonotic potential of Enterocytozoon genotypes in humans and pigs in Thailand. Veterinary Parasitology 2017, 233, 73-79.

87. Udonsom, R.; Prasertbun, R.; Mahittikorn, A.; Chiabchalard, R.; Sutthikornchai, C.; Palasuwan, A.; Popruk, S., Identification of *Enterocytozoon bieneusi* in goats and cattle in Thailand. Parasitol Res 2019, 15, (1), 308.

88. Prasertbun, R.; Mori, H.; Sukthana, Y.; Popruk, S.; Kusolsuk, T.; Hagiwara, K.; Mahittikorn, A., "Enterocytozoon bieneusi and Cryptosporidium: a cross-sectional study conducted throughout Thailand". Bmc Infect Dis 2019, 19, (1).

89. Agholi, M.; Hatam, G. R.; Motazedian, M. H., Microsporidia and Coccidia as Causes of Persistence Diarrhea among Liver Transplant Children: Incidence Rate and Species/Genotypes. Pediatr Infect Dis J 2013, 32, (2), 185-187.

90. Tabatabaie, F.; Abrehdari Tafreshi, Z.; Shahmohammad, N.; Pirestani, M., Molecular detection of microsporidiosis in various samples of Iranian immunocompromised patients. Journal of parasitic diseases : official organ of the Indian Society for Parasitology 2015, 39, (4), 634-8.

91. Kicia, M.; Wesolowska, M.; Kopacz, Z.; Jakuszko, K.; Sak, B.; Květonová, D.; Krajewska, M.; Kváč, M., Prevalence and molecular characteristics of urinary and intestinal microsporidia infections in renal transplant recipients. Clinical microbiology and infection : the official publication of the European Society of Clinical Microbiology and Infectious Diseases 2016, 22, (5), 462.e5-9.

92. Hassan, N. A.; Lim, Y. A. L.; Mahmud, R.; Mohd-Shaharuddin, N.; Wan Sulaiman, W. Y.; Ngui, R., Molecular Diagnosis of Microsporidia among Immunocompromised Patients in Kuala Lumpur, Malaysia. The American journal of tropical medicine and hygiene 2018, 99, (6), 1562-1566.

93. Kicia, M.; Szydlowicz, M.; Cebulski, K.; Jakuszko, K.; Piesiak, P.; Kowal, A.; Sak, B.; Krajewska, M.; Hendrich, A. B.; Kvac, M.; Kopacz, Z., Symptomatic respiratory Encephalitozoon cuniculi infection in renal transplant recipients. International journal of infectious diseases : IJID : official publication of the International Society for Infectious Diseases 2019, 79, 21-25.

94. Mirjalali, H.; Mirhendi, H.; Meamar, A. R.; Mohebali, M.; Askari, Z.; Mirsamadi, E. S.; Rezaeian, M., Genotyping and molecular analysis of *Enterocytozoon bieneusi* isolated from immunocompromised patients in Iran. Infect Genet Evol 2015, 36, 244-249.

95. Zhang, W.; Ren, G.; Zhao, W.; Yang, Z.; Shen, Y.; Sun, Y.; Liu, A.; Cao, J., Genotyping of *Enterocytozoon bieneusi* and Subtyping of Blastocystis in Cancer Patients: Relationship to Diarrhea and Assessment of Zoonotic Transmission. Front Microbiol 2017, 8.

96. Ghoyounchi, R.; Mahami-Oskouei, M.; Rezamand, A.; Spotin, A.; Aminisani, N.; Nami, S.; Pirestani, M.; Berahmat, R.; Madadi, S., Molecular Phylodiagnosis of *Enterocytozoon bieneusi* and Encephalitozoon intestinalis in Children with Cancer: Microsporidia in Malignancies as an Emerging Opportunistic Infection. Acta parasitologica 2019, 64, (1), 103-111.

97. Khanaliha, K.; Mirjalali, H.; Mohebali, M.; Tarighi, F.; Rezaeian, M., Comparison of Three Staining Methods for the Detection of Intestinal Microspora Spp. Iran J Parasitol 2014, 9, (4), 445-451.

98. Yu, F.; Li, D.; Chang, Y.; Wu, Y.; Guo, Z.; Jia, L.; Xu, J.; Li, J.; Qi, M.; Wang, R.; Zhang, L., Molecular characterization of three intestinal protozoans in hospitalized children with different disease backgrounds in Zhengzhou, central China. 2019, 12, (1), 543.

99. Tumwine, J. K.; Kekitiinwa, A.; Bakeera-Kitaka, S.; Ndeezi, G.; Downing, R.; Feng, X.; Akiyoshi, D. E.; Tzipori, S., Cryptosporidiosis and microsporidiosis in ugandan children with persistent diarrhea with and without concurrent infection with the human immunodeficiency virus. Am J Trop Med Hyg 2005, 73, (5), 921-5.

100. Raccurt, C. P.; Fouché, B.; Agnamey, P.; Menotti, J.; Chouaki, T.; Totet, A.; Pape, J. W., Presence of *Enterocytozoon bieneusi* associated with intestinal coccidia in patients with chronic diarrhea visiting an HIV center in Haiti. The American journal of tropical medicine and hygiene 2008, 79, (4), 579-80.

101. Nétor Velásquez, J.; Marta, E.; Alicia di Risio, C.; Etchart, C.; Gancedo, E.; Victor Chertcoff, A.; Bruno Malandrini, J.; Germán Astudillo, O.; Carnevale, S., Molecular identification of protozoa causing AIDS-associated cholangiopathy in Buenos Aires, Argentina. Acta gastroenterologica Latinoamericana 2012, 42, (4), 301-8.

102. Liu, H.; Shen, Y.; Yin, J.; Yuan, Z.; Jiang, Y.; Xu, Y.; Pan, W.; Hu, Y.; Cao, J., Prevalence and genetic characterization of Cryptosporidium, Enterocytozoon, Giardia and Cyclospora in diarrheal outpatients in China. Bmc Infect Dis 2014, 14, 25.

103. Bednarska, M.; Bajer, A.; Sinski, E.; Wolska-Kusnierz, B.; Samolinski, B.; Graczyk, T. M., Occurrence of intestinal microsporidia in immunodeficient patients in Poland. Annals of Agricultural and Environmental Medicine 2014, 21, (2), 244-248.

104. Taghipour, A.; Tabarsi, P.; Sohrabi, M. R.; Riahi, S. M.; Rostami, A.; Mirjalali, H.; Malih, N.; Haghighi, A., Frequency, associated factors and clinical symptoms of intestinal parasites among tuberculosis and non-tuberculosis groups in Iran: a comparative cross-sectional study. Transactions of the Royal Society of Tropical Medicine and Hygiene 2019, 113, (5), 234-241.

105. Jeong, D. K.; Won, G. Y.; Park, B. K.; Hur, J.; You, J. Y.; Kang, S. J.; Oh, I. G.; Lee, Y. S.; Stein, B. D.; Lee, J. H., Occurrence and genotypic characteristics of *Enterocytozoon bieneusi* in pigs with diarrhea. Parasitology research 2007, 102, (1), 123-8.

106. Sak, B.; Kvac, M.; Hanzlikova, D.; Cama, V., First report of *Enterocytozoon bieneusi* infection on a pig farm in the Czech Republic. Vet Parasitol 2008, 153, (3-4), 220-4.

107. Leelayoova, S.; Piyaraj, P.; Subrungruang, I.; Pagornrat, W.; Naaglor, T.; Phumklan, S.; Taamasri, P.; Suwanasri, J.; Mungthin, M., Genotypic characterization of *Enterocytozoon bieneusi* in specimens from pigs and humans in a pig farm community in Central Thailand. J Clin Microbiol 2009, 47, (5), 1572-4.

108. Li, W.; Diao, R.; Yang, J.; Xiao, L.; Lu, Y.; Li, Y.; Song, M., High diversity of human-pathogenic *Enterocytozoon bieneusi* genotypes in swine in northeast China. Parasitol Res 2014, 113, (3), 1147-53.

109. Li, W.; Tao, W.; Jiang, Y. X.; Diao, R. N.; Yang, J. P.; Xiao, L. H., Genotypic Distribution and Phylogenetic Characterization of *Enterocytozoon bieneusi* in Diarrheic Chickens and Pigs in Multiple Cities, China: Potential Zoonotic Transmission. Plos One 2014, 9, (9).

110. Li, W.; Li, Y. J.; Li, W. Z.; Yang, J. P.; Song, M. X.; Diao, R. N.; Jia, H. L.; Lu, Y. X.; Zheng, J.; Zhang, X. C.; Xiao, L. H., Genotypes of *Enterocytozoon bieneusi* in Livestock in China: High Prevalence and Zoonotic Potential. Plos One 2014, 9, (5).

111. Wan, Q.; Lin, Y.; Mao, Y.; Yang, Y.; Li, Q.; Zhang, S.; Jiang, Y.; Tao, W.; Li, W., High Prevalence and Widespread Distribution of Zoonotic *Enterocytozoon bieneusi* Genotypes in Swine in Northeast China: Implications for Public Health. J Eukaryot Microbiol 2016, 63, (2), 162-70.

112. Zou, Y.; Hou, J. L.; Li, F. C.; Zou, F. C.; Lin, R. Q.; Ma, J. G.; Zhang, X. X.; Zhu, X. Q., Prevalence and genotypes of *Enterocytozoon bieneusi* in pigs in southern China. Infect Genet Evol 2018, 66, 52-56.

113. Zhang, N.; Wu, R.; Ji, T.; Cui, L. L.; Cao, H. X.; Li, D.; Li, J.; Zhang, L.; Huang, C.; Zhou, D. H., Molecular Detection, Multilocus Genotyping, and Population Genetics of *Enterocytozoon bieneusi* in Pigs in Southeastern China. J Eukaryot Microbiol 2020, 67, (1), 107-114.

114. Li, D. F.; Zhang, Y.; Jiang, Y. X.; Xing, J. M.; Tao, D. Y.; Zhao, A. Y.; Cui, Z. H.; Jing, B.; Qi, M.; Zhang, L. X., Genotyping and Zoonotic Potential of *Enterocytozoon bieneusi* in Pigs in Xinjiang, China. Frontiers in microbiology 2019, 10, 2401.

115. Wang, S. S.; Li, J. Q.; Li, Y. H.; Wang, X. W.; Fan, X. C.; Liu, X.; Li, Z. J.; Song, J. K.; Zhang, L. X.; Zhao, G. H., Novel genotypes and multilocus genotypes of *Enterocytozoon bieneusi* in pigs in northwestern China: A public health concern. Infect Genet Evol 2018, 63, 89-94.

116. Valencakova, A.; Balent, P.; Huska, M.; Novotny, F.; Luptakova, L., First report on Encephalitozoon intestinalis infection of swine in Europe. Acta veterinaria Hungarica 2006, 54, (3), 407-11.

117. Nemejc, K.; Sak, B.; Kvetonova, D.; Hanzal, V.; Janiszewski, P.; Forejtek, P.; Rajsky, D.; Kotkova, M.; Ravaszova, P.; McEvoy, J.; Kvac, M., Prevalence and diversity of Encephalitozoon spp. and *Enterocytozoon bieneusi* in wild boars (Sus scrofa) in Central Europe. Parasitol Res 2014, 113, (2), 761-7.

118. Li, W.; Deng, L.; Wu, K.; Huang, X.; Song, Y.; Su, H.; Hu, Y.; Fu, H.; Zhong, Z.; Peng, G., Presence of zoonotic Cryptosporidium scrofarum, Giardia duodenalis assemblage A and *Enterocytozoon bieneusi* genotypes in captive Eurasian wild boars (Sus scrofa) in China: potential for zoonotic transmission. Parasit Vectors 2017, 10, (1), 10.

119. Feng, S.; Jia, T.; Huang, J.; Fan, Y.; Chang, H.; Han, S.; Luo, J.; He, H., Identification of *Enterocytozoon bieneusi* and Cryptosporidium spp. in farmed wild boars (Sus scrofa) in Beijing, China. Infect Genet Evol 2020, 80, 104231.

120. Luo, R.; Xiang, L.; Liu, H.; Zhong, Z.; Liu, L.; Deng, L.; Liu, L.; Huang, X.; Zhou, Z.; Fu, H.; Luo, Y.; Peng, G., First report and multilocus genotyping of *Enterocytozoon bieneusi* from Tibetan pigs in southwestern China. Parasite 2019, 26, 24.

121. Li, D.; Zheng, S.; Zhou, C.; Karim, M. R.; Wang, L.; Wang, H.; Yu, F.; Li, J.; Wang, W.; Wang, Y.; Zhang, S.; Jian, F.; Wang, R.; Ning, C.; Zhang, L., Multilocus Typing of *Enterocytozoon bieneusi* in Pig Reveals the High Prevalence, Zoonotic Potential, Host Adaptation and Geographical Segregation in China. J Eukaryot Microbiol 2019, 66, (5), 707-718.

122. Zou, Y.; Zheng, W. B.; Song, H. Y.; Xia, C. Y.; Shi, B.; Liu, J. Z.; Hou, J. L.; Zhu, X. Q., Prevalence and genetic characterization of *Enterocytozoon bieneusi* and Giardia duodenalis in Tibetan pigs in Tibet, China. Infect Genet Evol 2019, 75, 104019.

123. Santin, M.; Trout, J. M.; Vecino, J. A.; Dubey, J. P.; Fayer, R., Cryptosporidium, Giardia and *Enterocytozoon bieneusi* in cats from Bogota (Colombia) and genotyping of isolates. Vet Parasitol 2006, 141, (3-4), 334-9.

124. Mori, H.; Mahittikorn, A.; Thammasonthijarern, N.; Chaisiri, K.; Rojekittikhun, W.; Sukthana, Y., Presence of zoonotic *Enterocytozoon bieneusi* in cats in a temple in central Thailand. Vet Parasitol 2013, 197, (3-4), 696-701.

125. Karim, M. R.; Dong, H.; Yu, F.; Jian, F.; Zhang, L.; Wang, R.; Zhang, S.; Rume, F. I.; Ning, C.; Xiao, L., Genetic diversity in *Enterocytozoon bieneusi* isolates from dogs and cats in China: host specificity and public health implications. J Clin Microbiol 2014, 52, (9), 3297-302.

126. Li, W.; Li, Y.; Song, M.; Lu, Y.; Yang, J.; Tao, W.; Jiang, Y.; Wan, Q.; Zhang, S.; Xiao, L., Prevalence and genetic characteristics of Cryptosporidium, *Enterocytozoon bieneusi* and Giardia duodenalis in cats and dogs in Heilongjiang province, China. Vet Parasitol 2015, 208, (3-4), 125-34.

127. Zeinab Askari, H. M., Molecular detection and identification of zoonotic Microsporidia spore in fecal samples of some animals with close-contact to human. Journal of Parasitology Research 2015, 2012, 1-19.

128. Xu, H.; Jin, Y.; Wu, W.; Li, P.; Wang, L.; Li, N.; Feng, Y.; Xiao, L., Genotypes of Cryptosporidium spp., *Enterocytozoon bieneusi* and Giardia duodenalis in dogs and cats in Shanghai, China. Parasit Vectors 2016, 9, 121.

129. Ahmad Z. AL-HERRAWY, M. A. G., <Microsporidial Spores in Fecal Samples of Some Domesticated Animals Living in Giza, Egypt.pdf>. 2016.

130. Tsukada, R.; Osaka, Y.; Takano, T.; Sasaki, M.; Inose, M.; Ikadai, H., Serological survey of Encephalitozoon cuniculi infection in cats in Japan. The Journal of veterinary medical science 2016, 78, (10), 1615-1617.

131. Kvac, M.; Hofmannova, L.; Ortega, Y.; Holubova, N.; Horcickova, M.; Kicia, M.; Hlaskova, L.; Kvetonova, D.; Sak, B.; McEvoy, J., Stray cats are more frequently infected with zoonotic protists than pet cats. Folia parasitologica 2017, 64.

132. Piekarska, J.; Kicia, M.; Wesolowska, M.; Kopacz, Z.; Gorczykowski, M.; Szczepankiewicz, B.; Kvac, M.; Sak, B., Zoonotic microsporidia in dogs and cats in Poland. Vet Parasitol 2017, 246, 108-111.

133. Dashti, A.; Santin, M.; Cano, L.; de Lucio, A.; Bailo, B.; de Mingo, M. H.; Koster, P. C.; Fernandez-Basterra, J. A.; Aramburu-Aguirre, J.; Lopez-Molina, N.; Fernandez-Crespo, J. C.; Calero-Bernal, R.; Carmena, D., Occurrence and genetic diversity of *Enterocytozoon bieneusi* (Microsporidia) in owned and sheltered dogs and cats in Northern Spain. Parasitol Res 2019, 118, (10), 2979-2987.

134. Prado, J. B. F.; Ramos, C.; Fiuza, V.; Terra, V. J. B., Occurrence of zoonotic *Enterocytozoon bieneusi* in cats in Brazil. Revista brasileira de parasitologia veterinaria = Brazilian journal of veterinary parasitology : Orgao Oficial do Colegio Brasileiro de Parasitologia Veterinaria 2019, 28, (1), 80-90.

135. Pekmezci, D.; Pekmezci, G. Z.; Yildirim, A.; Duzlu, O.; Inci, A., Molecular Detection of Zoonotic Microsporidia in Domestic Cats in Turkey: A Preliminary Study. Acta Parasitol 2019, 64, (1), 13-18.

136. Mathis, A.; Breitenmoser, A. C.; Deplazes, P., Detection of newEnterocytozoongenotypes in faecal samples of farm dogs and a cat. Parasite 2014, 6, (2), 189-193.

137. Mónica Santín, J. A. C. V., and Ronald Fayer *Enterocytozoon bieneusi* Genotypes in Dogs in Bogota, Colombia. The American Society of Tropical Medicine and Hygiene 2008.

138. Abe, N.; Kimata, I.; Iseki, M., Molecular evidence of Enterocytozoon bieneusi in Japan. The Journal of veterinary medical science 2009, 71, (2), 217-9.

139. Galvan-Diaz, A. L.; Magnet, A.; Fenoy, S.; Henriques-Gil, N.; Haro, M.; Gordo, F. P.; Millan, J.; Miro, G.; del Aguila, C.; Izquierdo, F., Microsporidia detection and genotyping study of human pathogenic E. bieneusi in animals from Spain. PLoS One 2014, 9, (3), e92289.

140. Delrobaei, M.; Jamshidi, S.; Shayan, P.; Ebrahimzade, E.; Ashrafi Tamai, I.; Rezaeian, M.; Mirjalali, H., Molecular Detection and Genotyping of Intestinal Microsporidia from Stray Dogs in Iran. Iran J Parasitol 2019, 14, (1), 159-166.

141. Duzlu, O.; Yildirim, A.; Onder, Z.; Ciloglu, A.; Yetismis, G.; Inci, A., Prevalence and Genotyping of Microsporidian Parasites in Dogs in Turkey: Zoonotic Concerns. J Eukaryot Microbiol 2019, 66, (5), 771-777.

142. Phrompraphai, T.; Itoh, N.; Iijima, Y.; Ito, Y.; Kimura, Y., Molecular detection and genotyping of *Enterocytozoon bieneusi* in family pet dogs obtained from different routes in Japan. Parasitology international 2019, 70, 86-88.

143. Heinz Rinder, A. T., Bianca Dengjel, Rainer Gothe, Thomas Löscher, and Monika Zahler Close Genotypic Relationship between *Enterocytozoon bieneusi* from Humans and Pigs and First Detection in Cattle. American Society of Parasitologists 2000.

144. Sulaiman, I. M.; Fayer, R.; Yang, C. F.; Santin, M.; Matos, O.; Xiao, L. H., Molecular characterization of *Enterocytozoon bieneusi* in cattle indicates that only some isolates have zoonotic potential. Parasitol Res 2004, 92, (4), 328-334.

145. Santin, M.; Trout, J. M.; Fayer, R., *Enterocytozoon bieneusi* genotypes in dairy cattle in the eastern United States. Parasitol Res 2005, 97, (6), 535-8.

146. Fayer, R.; Santin, M.; Trout, J. M., *Enterocytozoon bieneusi* in mature dairy cattle on farms in the eastern United States. Parasitol Res 2007, 102, (1), 15-20.

147. Lee, J. H., Prevalence and molecular characteristics of *Enterocytozoon bieneusi* in cattle in Korea. Parasitol Res 2007, 101, (2), 391-6.

148. Lee, J. H., Molecular detection of *Enterocytozoon bieneusi* and identification of a potentially human-pathogenic genotype in milk. Appl Environ Microbiol 2008, 74, (5), 1664-6.

149. Fayer, R.; Santin, M.; Macarisin, D., Detection of concurrent infection of dairy cattle with Blastocystis, Cryptosporidium, Giardia, and Enterocytozoon by molecular and microscopic methods. Parasitol Res 2012, 111, (3), 1349-55.

150. Santin, M.; Dargatz, D.; Fayer, R., Prevalence and genotypes of *Enterocytozoon bieneusi* in weaned beef calves on cow-calf operations in the USA. Parasitol Res 2012, 110, (5), 2033-41.

151. Jurankova, J.; Kamler, M.; Kovarcik, K.; Koudela, B., *Enterocytozoon bieneusi* in Bovine Viral Diarrhea Virus (BVDV) infected and noninfected cattle herds. Res Vet Sci 2013, 94, (1), 100-4.

152. Del Coco, V. F.; Cordoba, M. A.; Bilbao, G.; de Almeida Castro, P.; Basualdo, J. A.; Santin, M., First report of *Enterocytozoon bieneusi* from dairy cattle in Argentina. Vet Parasitol 2014, 199, (1-2), 112-5.

153. Ma, J.; Li, P.; Zhao, X.; Xu, H.; Wu, W.; Wang, Y.; Guo, Y.; Wang, L.; Feng, Y.; Xiao, L., Occurrence and molecular characterization of Cryptosporidium spp. and *Enterocytozoon bieneusi* in dairy cattle, beef cattle and water buffaloes in China. Vet Parasitol 2015, 207, (3-4), 220-7.

154. Zhao, W.; Zhang, W.; Yang, F.; Zhang, L.; Wang, R.; Cao, J.; Shen, Y.; Liu, A., *Enterocytozoon bieneusi* in Dairy Cattle in the Northeast of China: Genetic Diversity of ITS Gene and Evaluation of Zoonotic Transmission Potential. J Eukaryot Microbiol 2015, 62, (4), 553-60.

155. da Silva Fiuza, V. R.; Lopes, C. W.; de Oliveira, F. C.; Fayer, R.; Santin, M., New findings of *Enterocytozoon bieneusi* in beef and dairy cattle in Brazil. Vet Parasitol 2016, 216, 46-51.

156. Li, J.; Luo, N.; Wang, C.; Qi, M.; Cao, J.; Cui, Z.; Huang, J.; Wang, R.; Zhang, L., Occurrence, molecular characterization and predominant genotypes of *Enterocytozoon bieneusi* in dairy cattle in Henan and Ningxia, China. Parasit Vectors 2016, 9, 142.

157. Wang, X. T.; Wang, R. J.; Ren, G. J.; Yu, Z. Q.; Zhang, L. X.; Zhang, S. Y.; Lu, H.; Peng, X. Q.; Zhao, G. H., Multilocus genotyping of Giardia duodenalis and *Enterocytozoon bieneusi* in dairy and native beef (Qinchuan) calves in Shaanxi province, northwestern China. Parasitol Res 2016, 115, (3), 1355-61.

158. Al-Herrawy, A. Z.; Gad, M. A., Microsporidial Spores in Fecal Samples of Some Domesticated Animals Living in Giza, Egypt. Iranian journal of parasitology 2016, 11, (2), 195-203.

159. Hu, S.; Liu, Z.; Yan, F.; Zhang, Z.; Zhang, G.; Zhang, L.; Jian, F.; Zhang, S.; Ning, C.; Wang, R., Zoonotic and host-adapted genotypes of Cryptosporidium spp., Giardia duodenalis and *Enterocytozoon bieneusi* in dairy cattle in Hebei and Tianjin, China. Vet Parasitol 2017, 248, 68-73.

160. Qi, M.; Jing, B.; Jian, F.; Wang, R.; Zhang, S.; Wang, H.; Ning, C.; Zhang, L., Dominance of *Enterocytozoon bieneusi* genotype J in dairy calves in Xinjiang, Northwest China. Parasitol Int 2017, 66, (1), 960-963.

161. Tang, C.; Cai, M.; Wang, L.; Guo, Y.; Li, N.; Feng, Y.; Xiao, L., Genetic diversity within dominant *Enterocytozoon bieneusi* genotypes in pre-weaned calves. Parasit Vectors 2018, 11, (1), 170.

162. Kord-Sarkachi, E.; Tavalla, M.; Beiromvand, M., Molecular diagnosis of microsporidia strains in slaughtered cows of southwest of Iran. Journal of parasitic diseases : official organ of the Indian Society for Parasitology 2018, 42, (1), 81-86.

163. Feng, Y.; Gong, X.; Zhu, K.; Li, N.; Yu, Z.; Guo, Y.; Weng, Y.; Kvac, M.; Feng, Y.; Xiao, L., Prevalence and genotypic identification of Cryptosporidium spp., Giardia duodenalis and *Enterocytozoon bieneusi* in pre-weaned dairy calves in Guangdong, China. Parasit Vectors 2019, 12, (1), 41.

164. Yu, F.; Qi, M.; Zhao, Z.; Lv, C.; Wang, Y.; Wang, R.; Zhang, L., The Potential Role of Synanthropic Rodents and Flies in the Transmission of *Enterocytozoon bieneusi* on a Dairy Cattle farm in China. J Eukaryot Microbiol 2019, 66, (3), 435-441.

165. Zhang, Y.; Koehler, A. V.; Wang, T.; Haydon, S. R.; Gasser, R. B., *Enterocytozoon bieneusi* Genotypes in Cattle on Farms Located within a Water Catchment Area. J Eukaryot Microbiol 2019, 66, (4), 553-559.

166. Udonsom, R.; Prasertbun, R.; Mahittikorn, A.; Chiabchalard, R.; Sutthikornchai, C.; Palasuwan, A.; Popruk, S., Identification of *Enterocytozoon bieneusi* in goats and cattle in Thailand. BMC veterinary research 2019, 15, (1), 308.

167. Zhao, W.; Wang, J.; Ren, G.; Yang, Z.; Yang, F.; Zhang, W.; Xu, Y.; Liu, A.; Ling, H., Molecular characterizations of Cryptosporidium spp. and *Enterocytozoon bieneusi* in brown rats (Rattus norvegicus) from Heilongjiang Province, China. Parasit Vectors 2018, 11, (1), 313.

168. Zhang, Q.; Zhang, Z.; Ai, S.; Wang, X.; Zhang, R.; Duan, Z., Cryptosporidium spp., *Enterocytozoon bieneusi*, and Giardia duodenalis from animal sources in the Qinghai-Tibetan Plateau Area (QTPA) in China. Comparative immunology, microbiology and infectious diseases 2019, 67, 101346.

169. Wang, R.; Li, N.; Jiang, W.; Guo, Y.; Wang, X.; Jin, Y.; Feng, Y.; Xiao, L., Infection patterns, clinical significance, and genetic characteristics of *Enterocytozoon bieneusi* and Giardia duodenalis in dairy cattle in Jiangsu, China. Parasitol Res 2019, 118, (10), 3053-3060.

170. Tao, W. F.; Ni, H. B.; Du, H. F.; Jiang, J.; Li, J.; Qiu, H. Y.; Ye, L.; Zhang, X. X., Molecular detection of Cryptosporidium and *Enterocytozoon bieneusi* in dairy calves and sika deer in four provinces in Northern China. Parasitol Res 2020, 119, (1), 105-114.

171. Samra, N. A.; Thompson, P. N.; Jori, F.; Zhang, H.; Xiao, L., *Enterocytozoon bieneusi* at the wildlife/livestock interface of the Kruger National Park, South Africa. Veterinary Parasitology 2012, 190, (3-4), 587-590.

172. Wu, Y.; Chang, Y.; Zhang, X.; Chen, Y.; Li, D.; Wang, L.; Zheng, S.; Wang, R.; Zhang, S.; Jian, F.; Ning, C.; Li, J.; Zhang, L., Molecular characterization and distribution of Cryptosporidium spp., Giardia duodenalis, and *Enterocytozoon bieneusi* from yaks in Tibet, China. BMC Vet Res 2019, 15, (1), 417.

173. Ma, J.; Cai, J.; Ma, J.; Feng, Y.; Xiao, L., *Enterocytozoon bieneusi* genotypes in yaks (Bos grunniens) and their public health potential. J Eukaryot Microbiol 2015, 62, (1), 21-5.

174. Ma, J. G.; Zhang, N. Z.; Hou, J. L.; Zou, Y.; Hu, G. X.; Zhu, X. Q.; Zhou, D. H., Detection of *Enterocytozoon bieneusi* in White Yaks in Gansu Province, China. BioMed research international 2017, 2017, 5790181.

175. Zhang, Q.; Cai, J.; Li, P.; Wang, L.; Guo, Y.; Li, C.; Lei, M.; Feng, Y.; Xiao, L., *Enterocytozoon bieneusi* genotypes in Tibetan sheep and yaks. Parasitol Res 2018, 117, (3), 721-727.

176. Li, F.; Wang, R.; Guo, Y.; Li, N.; Feng, Y.; Xiao, L., Zoonotic potential of *Enterocytozoon bieneusi* and Giardia duodenalis in horses and donkeys in northern China. Parasitol Res 2020.

177. Laatamna, A. E.; Wagnerova, P.; Sak, B.; Kvetonova, D.; Xiao, L.; Rost, M.; McEvoy, J.; Saadi, A. R.; Aissi, M.; Kvac, M., Microsporidia and Cryptosporidium in horses and donkeys in Algeria: detection of a novel Cryptosporidium hominis subtype family (Ik) in a horse. Vet Parasitol 2015, 208, (3-4), 135-42.

178. Santin, M.; Vecino, J. A.; Fayer, R., A zoonotic genotype of *Enterocytozoon bieneusi* in horses. The Journal of parasitology 2010, 96, (1), 157-61.

179. Wagnerova, P.; Sak, B.; Kvetonova, D.; Bunatova, Z.; Civisova, H.; Marsalek, M.; Kvac, M., *Enterocytozoon bieneusi* and Encephalitozoon cuniculi in horses kept under different management systems in the Czech Republic. Vet Parasitol 2012, 190, (3-4), 573-7.

180. Deng, L.; Li, W.; Zhong, Z.; Gong, C.; Liu, X.; Huang, X.; Xiao, L.; Zhao, R.; Wang, W.; Feng, F.; Zhang, Y.; Hu, Y.; Fu, H.; He, M.; Zhang, Y.; Wu, K.; Peng, G., Molecular characterization and multilocus genotypes of *Enterocytozoon bieneusi* among horses in southwestern China. Parasit Vectors 2016, 9, (1), 561.

181. Wagnerova, P.; Sak, B.; McEvoy, J.; Rost, M.; Sherwood, D.; Holcomb, K.; Kvac, M., Cryptosporidium parvum and *Enterocytozoon bieneusi* in American Mustangs and Chincoteague ponies. Exp Parasitol 2016, 162, 24-7.

182. Zhao, A.; Li, D.; Wei, Z.; Zhang, Y.; Peng, Y.; Zhu, Y.; Qi, M.; Zhang, L., Molecular Detection and Genotyping of *Enterocytozoon bieneusi* in Racehorses in China. Front Microbiol 2019, 10, 1920.

183. Yue, D. M.; Ma, J. G.; Li, F. C.; Hou, J. L.; Zheng, W. B.; Zhao, Q.; Zhang, X. X.; Zhu, X. Q., Occurrence of *Enterocytozoon bieneusi* in Donkeys (Equus asinus) in China: A Public Health Concern. Frontiers in microbiology 2017, 8, 565.

184. Qi, M.; Li, J.; Zhao, A.; Cui, Z.; Wei, Z.; Jing, B.; Zhang, L., Host specificity of *Enterocytozoon bieneusi* genotypes in Bactrian camels (Camelus bactrianus) in China. Parasit Vectors 2018, 11, (1), 219.

185. Zhang, Q.; Wang, H.; Zhao, A.; Zhao, W.; Wei, Z.; Li, Z.; Qi, M., Molecular detection of *Enterocytozoon bieneusi* in alpacas (Vicugna pacos) in Xinjiang, China. Parasite 2019, 26, 31.

186. Stensvold, C. R.; Beser, J.; Ljungstrom, B.; Troell, K.; Lebbad, M., Low host-specific Enterocytozoon bieneusi genotype BEB6 is common in Swedish lambs. Vet Parasitol 2014, 205, (1-2), 371-4.

187. Li, W.; Li, Y.; Li, W.; Yang, J.; Song, M.; Diao, R.; Jia, H.; Lu, Y.; Zheng, J.; Zhang, X.; Xiao, L., Genotypes of *Enterocytozoon bieneusi* in livestock in China: high prevalence and zoonotic potential. PLoS One 2014, 9, (5), e97623.

188. Ye, J.; Xiao, L.; Wang, Y.; Guo, Y.; Roellig, D. M.; Feng, Y., Dominance of Giardia duodenalis assemblage A and *Enterocytozoon bieneusi* genotype BEB6 in sheep in Inner Mongolia, China. Vet Parasitol 2015, 210, (3-4), 235-9.

189. Jiang, Y.; Tao, W.; Wan, Q.; Li, Q.; Yang, Y.; Lin, Y.; Zhang, S.; Li, W., Zoonotic and Potentially Host-Adapted *Enterocytozoon bieneusi* Genotypes in Sheep and Cattle in Northeast China and an Increasing Concern about the Zoonotic Importance of Previously Considered Ruminant-Adapted Genotypes. Appl Environ Microbiol 2015, 81, (10), 3326-35.

190. Li, J.; Qi, M.; Chang, Y.; Wang, R.; Li, T.; Dong, H.; Zhang, L., Molecular Characterization ofCryptosporidiumspp.,Giardia duodenalis, and*Enterocytozoon bieneusi*in Captive Wildlife at Zhengzhou Zoo, China. J Eukaryot Microbiol 2015, 62, (6), 833-839.

191. Zhao, W.; Zhang, W.; Yang, D.; Zhang, L.; Wang, R.; Liu, A., Prevalence of *Enterocytozoon bieneusi* and genetic diversity of ITS genotypes in sheep and goats in China. Infect Genet Evol 2015, 32, 265-70.

192. Fiuza VRDS, L. C., Cosendey RIJ, de Oliveira FCR, Fayer R, Santín M, Zoonotic *Enterocytozoon bieneusi* genotypes found in brazilian sheep. Res Vet Sci 2016.

193. Shi, K.; Li, M.; Wang, X.; Li, J.; Karim, M. R.; Wang, R.; Zhang, L.; Jian, F.; Ning, C., Molecular survey of *Enterocytozoon bieneusi* in sheep and goats in China. Parasit Vectors 2016, 9, 23.

194. Yang, H.; Mi, R.; Cheng, L.; Huang, Y.; An, R.; Zhang, Y.; Jia, H.; Zhang, X.; Wang, X.; Han, X.; Chen, Z., Prevalence and genetic diversity of *Enterocytozoon bieneusi* in sheep in China. Parasit Vectors 2018, 11, (1), 587.

195. Wu, Y.; Chang, Y.; Chen, Y.; Zhang, X.; Li, D.; Zheng, S.; Wang, L.; Li, J.; Ning, C.; Zhang, L., Occurrence and molecular characterization of Cryptosporidium spp., Giardia duodenalis, and *Enterocytozoon bieneusi* from Tibetan sheep in Gansu, China. Infect Genet Evol 2018, 64, 46-51.

196. Li, W. C.; Wang, K.; Gu, Y. F., Detection and Genotyping Study of *Enterocytozoon bieneusi* in Sheep and Goats in East-central China. Acta Parasitol 2019, 64, (1), 44-50.

197. Qi, M.; Zhang, Z.; Zhao, A.; Jing, B.; Guan, G.; Luo, J.; Zhang, L., Distribution and molecular characterization of Cryptosporidium spp., Giardia duodenalis, and *Enterocytozoon bieneusi* amongst grazing adult sheep in Xinjiang, China. Parasitol Int 2019, 71, 80-86.

198. Peng, J. J.; Zou, Y.; Li, Z. X.; Liang, Q. L.; Song, H. Y.; Li, T. S.; Ma, Y. Y.; Zhu, X. Q.; Zhou, D. H., Occurrence of *Enterocytozoon bieneusi* in Chinese Tan sheep in the Ningxia Hui Autonomous Region, China. Parasitol Res 2019, 118, (9), 2729-2734.

199. Chang, Y.; Wang, Y.; Wu, Y.; Niu, Z.; Li, J.; Zhang, S.; Wang, R.; Jian, F.; Ning, C.; Zhang, L., Molecular Characterization of Giardia duodenalis and *Enterocytozoon bieneusi* Isolated from Tibetan Sheep and Tibetan Goats Under Natural Grazing Conditions in Tibet. J Eukaryot Microbiol 2020, 67, (1), 100-106.

200. Sak, B.; Petrzelkova, K. J.; Kvetonova, D.; Mynarova, A.; Pomajbikova, K.; Modry, D.; Cranfield, M. R.; Mudakikwa, A.; Kvac, M., Diversity of microsporidia, Cryptosporidium and Giardia in mountain gorillas (Gorilla beringei beringei) in Volcanoes National Park, Rwanda. PLoS One 2014, 9, (11), e109751.

201. Peng, X. Q.; Tian, G. R.; Ren, G. J.; Yu, Z. Q.; Lok, J. B.; Zhang, L. X.; Wang, X. T.; Song, J. K.; Zhao, G. H., Infection rate of Giardia duodenalis, Cryptosporidium spp. and *Enterocytozoon bieneusi* in cashmere, dairy and meat goats in China. Infect Genet Evol 2016, 41, 26-31.

202. Eberhard, M. L.; Arrowood, M. J., Cryptosporidium spp., *Enterocytozoon bieneusi*, and Giardia duodenalis from animal sources in the Qinghai-Tibetan Plateau Area (QTPA) in China. Curr Opin Infect Dis 2002, 15, (5), 519-22.

203. Zhou, H. H.; Zheng, X. L.; Ma, T. M.; Qi, M.; Cao, Z. X.; Chao, Z.; Wei, L. M.; Liu, Q. W.; Sun, R. P.; Wang, F.; Zhang, Y.; Lu, G.; Zhao, W., Genotype identification and phylogenetic analysis of *Enterocytozoon bieneusi* in farmed black goats (Capra hircus) from China's Hainan Province. Parasite 2019, 26, 62.

204. Wegayehu, T.; Li, J.; Karim, M. R.; Zhang, L., Molecular Characterization and Phylogenetic Analysis of *Enterocytozoon bieneusi* in Lambs in Oromia Special Zone, Central Ethiopia. Frontiers in veterinary science 2020, 7, 6.

205. Zhao, W.; Zhang, W.; Wang, R.; Liu, W.; Liu, A.; Yang, D.; Yang, F.; Karim, M. R.; Zhang, L., *Enterocytozoon bieneusi* in sika deer (Cervus nippon) and red deer (Cervus elaphus): deer specificity and zoonotic potential of ITS genotypes. Parasitol Res 2014, 113, (11), 4243-50.

206. Guo, Y.; Alderisio, K. A.; Yang, W.; Cama, V.; Feng, Y.; Xiao, L., Host specificity and source of *Enterocytozoon bieneusi* genotypes in a drinking source watershed. Appl Environ Microbiol 2014, 80, (1), 218-25.

207. Santin, M.; Fayer, R., *Enterocytozoon bieneusi*, giardia, and Cryptosporidium infecting white-tailed deer. J Eukaryot Microbiol 2015, 62, (1), 34-43.

208. Liu, W.; Nie, C.; Zhang, L.; Wang, R.; Liu, A.; Zhao, W.; Li, H., First detection and genotyping of *Enterocytozoon bieneusi* in reindeers (Rangifer tarandus): a zoonotic potential of ITS genotypes. Parasit Vectors 2015, 8, 526.

209. Zhang, Z.; Huang, J.; Karim, M. R.; Zhao, J.; Dong, H.; Ai, W.; Li, F.; Zhang, L.; Wang, R., Zoonotic *Enterocytozoon bieneusi* genotypes in Pere David's deer (Elaphurus davidianus) in Henan, China. Exp Parasitol 2015, 155, 46-8.

210. Li, W.; Deng, L.; Yu, X.; Zhong, Z.; Wang, Q.; Liu, X.; Niu, L.; Xie, N.; Deng, J.; Lei, S.; Wang, L.; Gong, C.; Zhou, Z.; Hu, Y.; Fu, H.; Xu, H.; Geng, Y.; Peng, G., Multilocus genotypes and broad host-range of *Enterocytozoon bieneusi* in captive wildlife at zoological gardens in China. Parasites & Vectors 2016, 9, (1).

211. Zhang, X. X.; Cong, W.; Liu, G. H.; Ni, X. T.; Ma, J. G.; Zheng, W. B.; Zhao, Q.; Zhu, X. Q., Prevalence and genotypes of *Enterocytozoon bieneusi* in sika deer in Jilin province, Northeastern China. Acta Parasitol 2016, 61, (2), 382-8.

212. Huang, J.; Zhang, Z.; Yang, Y.; Wang, R.; Zhao, J.; Jian, F.; Ning, C.; Zhang, L., New Genotypes of *Enterocytozoon bieneusi* Isolated from Sika Deer and Red Deer in China. Front Microbiol 2017, 8, 879.

213. Zhao, W.; Wang, J.; Yang, Z.; Liu, A., Dominance of the *Enterocytozoon bieneusi* genotype BEB6 in red deer (Cervus elaphus) and Siberian roe deer (Capreolus pygargus) in China and a brief literature review. Parasite 2017, 24, 54.

214. Zhang, Y.; Koehler, A. V.; Wang, T.; Haydon, S. R.; Gasser, R. B., First detection and genetic characterisation of *Enterocytozoon bieneusi* in wild deer in Melbourne's water catchments in Australia. Parasit Vectors 2018, 11, (1), 2.

215. Song, Y.; Li, W.; Liu, H.; Zhong, Z.; Luo, Y.; Wei, Y.; Fu, W.; Ren, Z.; Zhou, Z.; Deng, L.; Cheng, J.; Peng, G., First report of Giardia duodenalis and *Enterocytozoon bieneusi* in forest musk deer (Moschus berezovskii) in China. Parasit Vectors 2018, 11, (1), 204.

216. Amer, S.; Kim, S.; Han, J. I.; Na, K. J., Prevalence and genotypes of *Enterocytozoon bieneusi* in wildlife in Korea: a public health concern. Parasit Vectors 2019, 12, (1), 160.

217. Casey E. Barton, D. N. P., Prevalence of Microsporidian Spores Shed by Asymptomatic Lovebirds: Evidence for a Potential Emerging Zoonosis. Avian Medicine and Surgery 2003.

218. Baroudi, D.; Zhang, H.; Amer, S.; Khelef, D.; Roellig, D. M.; Wang, Y.; Feng, Y.; Xiao, L., Divergent Cryptosporidium parvum subtype and *Enterocytozoon bieneusi* genotypes in dromedary camels in Algeria. Parasitol Res 2018, 117, (3), 905-910.

219. Koehler, A. V.; Rashid, M. H.; Zhang, Y.; Vaughan, J. L.; Gasser, R. B.; Jabbar, A., First cross-sectional, molecular epidemiological survey of Cryptosporidium, Giardia and Enterocytozoon in alpaca (Vicugna pacos) in Australia. Parasit Vectors 2018, 11, (1), 498.

220. Ma, Y. T.; Zou, Y.; Liu, Q.; Xie, S. C.; Li, R. L.; Zhu, X. Q.; Gao, W. W., Prevalence and multilocus genotypes of *Enterocytozoon bieneusi* in alpacas (Vicugna pacos) in Shanxi Province, northern China. Parasitol Res 2019, 118, (12), 3371-3375.

221. Sak, B.; Kvác, M.; Petrzelková, K.; Kvetonová, D.; Pomajbíková, K.; Mulama, M.; Kiyang, J.; Modrý, D., Diversity of microsporidia (Fungi: Microsporidia) among captive great apes in European zoos and African sanctuaries: evidence for zoonotic transmission? Folia parasitologica 2011, 58, (2), 81-6.

222. Li, W.; Kiulia, N. M.; Mwenda, J. M.; Nyachieo, A.; Taylor, M. B.; Zhang, X.; Xiao, L., Cyclospora papionis, Cryptosporidium hominis, and human-pathogenic *Enterocytozoon bieneusi* in captive baboons in Kenya. J Clin Microbiol 2011, 49, (12), 4326-9.

223. Ye, J.; Xiao, L.; Ma, J.; Guo, M.; Liu, L.; Feng, Y., Anthroponotic enteric parasites in monkeys in public park, China. Emerging infectious diseases 2012, 18, (10), 1640-3.

224. Sak, B.; Petrzelkova, K. J.; Kvetonova, D.; Mynarova, A.; Shutt, K. A.; Pomajbikova, K.; Kalousova, B.; Modry, D.; Benavides, J.; Todd, A.; Kvac, M., Long-term monitoring of microsporidia, Cryptosporidium and Giardia infections in western Lowland Gorillas (Gorilla gorilla gorilla) at different stages of habituation in Dzanga Sangha Protected Areas, Central African Republic. PLoS One 2013, 8, (8), e71840.

225. Ye, J.; Xiao, L.; Li, J.; Huang, W.; Amer, S. E.; Guo, Y.; Roellig, D.; Feng, Y., Occurrence of human-pathogenic *Enterocytozoon bieneusi*, Giardia duodenalis and Cryptosporidium genotypes in laboratory macaques in Guangxi, China. Parasitol Int 2014, 63, (1), 132-7.

226. Karim, M. R.; Wang, R.; Dong, H.; Zhang, L.; Li, J.; Zhang, S.; Rume, F. I.; Qi, M.; Jian, F.; Sun, M.; Yang, G.; Zou, F.; Ning, C.; Xiao, L., Genetic polymorphism and zoonotic potential of *Enterocytozoon bieneusi* from nonhuman primates in China. Appl Environ Microbiol 2014, 80, (6), 1893-8.

227. Karim, M. R.; Wang, R.; He, X.; Zhang, L.; Li, J.; Rume, F. I.; Dong, H.; Qi, M.; Jian, F.; Zhang, S.; Sun, M.; Yang, G.; Zou, F.; Ning, C.; Xiao, L., Multilocus sequence typing of *Enterocytozoon bieneusi* in nonhuman primates in China. Vet Parasitol 2014, 200, (1-2), 13-23.

228. Du, S. Z.; Zhao, G. H.; Shao, J. F.; Fang, Y. Q.; Tian, G. R.; Zhang, L. X.; Wang, R. J.; Wang, H. Y.; Qi, M.; Yu, S. K., Cryptosporidium spp., Giardia intestinalis, and *Enterocytozoon bieneusi* in Captive Non-Human Primates in Qinling Mountains. Korean J Parasitol 2015, 53, (4), 395-402.

229. Karim, M. R.; Dong, H.; Li, T.; Yu, F.; Li, D.; Zhang, L.; Li, J.; Wang, R.; Li, S.; Li, X.; Rume, F. I.; Ning, C., Predomination and new genotypes of *Enterocytozoon bieneusi* in captive nonhuman primates in zoos in China: high genetic diversity and zoonotic significance. PLoS One 2015, 10, (2), e0117991.

230. Mynarova, A.; Foitova, I.; Kvac, M.; Kvetonova, D.; Rost, M.; Morrogh-Bernard, H.; Nurcahyo, W.; Nguyen, C.; Supriyadi, S.; Sak, B., Prevalence of Cryptosporidium spp., *Enterocytozoon bieneusi*, Encephalitozoon spp. and Giardia intestinalis in Wild, Semi-Wild and Captive Orangutans (Pongo abelii and Pongo pygmaeus) on Sumatra and Borneo, Indonesia. PLoS One 2016, 11, (3), e0152771.

231. Yang, H.; Lin, Y.; Li, Y.; Song, M.; Lu, Y.; Li, W., Molecular characterization of *Enterocytozoon bieneusi* isolates in laboratory macaques in north China: zoonotic concerns. Parasitol Res 2017, 116, (10), 2877-2882.

232. Yu, F.; Wu, Y.; Li, T.; Cao, J.; Wang, J.; Hu, S.; Zhu, H.; Zhang, S.; Wang, R.; Ning, C.; Zhang, L., High prevalence of *Enterocytozoon bieneusi* zoonotic genotype D in captive golden snub-nosed monkey (Rhinopithecus roxellanae) in zoos in China. BMC Vet Res 2017, 13, (1), 158.

233. Chen, L.; Zhao, J.; Li, N.; Guo, Y.; Feng, Y.; Feng, Y.; Xiao, L., Genotypes and public health potential of *Enterocytozoon bieneusi* and Giardia duodenalis in crab-eating macaques. Parasit Vectors 2019, 12, (1), 254.

234. Zhao, W.; Zhou, H.; Jin, H.; Sun, L.; Li, P.; Liu, M.; Qiu, M.; Xu, L.; Li, F.; Ma, T.; Wang, S.; Yin, F.; Li, L.; Cui, X.; Chan, J. F.; Lu, G., Genotyping of *Enterocytozoon bieneusi* among captive long-tailed macaques (Macaca fascicularis) in Hainan Province: High genetic diversity and zoonotic potential. Acta Trop 2020, 201, 105211.

235. Deng, L.; Li, W.; Yu, X.; Gong, C.; Liu, X.; Zhong, Z.; Xie, N.; Lei, S.; Yu, J.; Fu, H.; Chen, H.; Xu, H.; Hu, Y.; Peng, G., First Report of the Human-Pathogenic *Enterocytozoon bieneusi* from Red-Bellied Tree Squirrels (Callosciurus erythraeus) in Sichuan, China. PLoS One 2016, 11, (9), e0163605.

236. Zhong, Z.; Tian, Y.; Song, Y.; Deng, L.; Li, J.; Ren, Z.; Ma, X.; Gu, X.; He, C.; Geng, Y.; Peng, G., Molecular characterization and multi-locus genotypes of *Enterocytozoon bieneusi* from captive red kangaroos (Macropus Rufus) in Jiangsu province, China. PLoS One 2017, 12, (8), e0183249.

237. Deng, L.; Li, W.; Zhong, Z.; Chai, Y.; Yang, L.; Zheng, H.; Wang, W.; Fu, H.; He, M.; Huang, X.; Zuo, Z.; Wang, Y.; Cao, S.; Liu, H.; Ma, X.; Wu, K.; Peng, G., Molecular characterization and new genotypes of *Enterocytozoon bieneusi* in pet chipmunks (Eutamias asiaticus) in Sichuan province, China. BMC microbiology 2018, 18, (1), 37.

238. Perec-Matysiak, A.; Lesnianska, K.; Bunkowska-Gawlik, K.; Condlova, S.; Sak, B.; Kvac, M.; Rajsky, D.; Hildebrand, J., The opportunistic pathogen Encephalitozoon cuniculi in wild living Murinae and Arvicolinae in Central Europe. European journal of protistology 2019, 69, 14-19.

239. Wang, H.; Liu, Q.; Jiang, X.; Zhang, Y.; Zhao, A.; Cui, Z.; Li, D.; Qi, M.; Zhang, L., Dominance of zoonotic genotype D of *Enterocytozoon bieneusi* in bamboo rats (Rhizomys sinensis). Infect Genet Evol 2019, 73, 113-118.

240. Deng, L.; Chai, Y.; Luo, R.; Yang, L.; Yao, J.; Zhong, Z.; Wang, W.; Xiang, L.; Fu, H.; Liu, H.; Zhou, Z.; Yue, C.; Chen, W.; Peng, G., Occurrence and genetic characteristics of Cryptosporidium spp. and *Enterocytozoon bieneusi* in pet red squirrels (Sciurus vulgaris) in China. Sci Rep 2020, 10, (1), 1026.

241. Li, J.; Jiang, Y.; Wang, W.; Chao, L.; Jia, Y.; Yuan, Y.; Wang, J.; Qiu, J.; Qi, M., Molecular identification and genotyping of *Enterocytozoon bieneusi* in experimental rats in China. Exp Parasitol 2020, 210, 107850.

242. Perec-Matysiak A, B.-G. K., Kváč M, Sak B, Hildebrand J, Leśniańska K, Diversity of *Enterocytozoon bieneusi* genotypes among small rodents in southwestern Poland. 2015.

243. Gui, B. Z.; Zou, Y.; Chen, Y. W.; Li, F.; Jin, Y. C.; Liu, M. T.; Yi, J. N.; Zheng, W. B.; Liu, G. H., Novel genotypes and multilocus genotypes of *Enterocytozoon bieneusi* in two wild rat species in China: potential for zoonotic transmission. Parasitol Res 2020, 119, (1), 283-290.

244. Yang, Y.; Lin, Y.; Li, Q.; Zhang, S.; Tao, W.; Wan, Q.; Jiang, Y.; Li, W., Widespread presence of human-pathogenic *Enterocytozoon bieneusi* genotype D in farmed foxes (Vulpes vulpes) and raccoon dogs (Nyctereutes procyonoides) in China: first identification and zoonotic concern. Parasitol Res 2015, 114, (11), 4341-8.

245. Zhao, W.; Zhang, W.; Yang, Z.; Liu, A.; Zhang, L.; Yang, F.; Wang, R.; Ling, H., Genotyping of *Enterocytozoon bieneusi* in Farmed Blue Foxes (Alopex lagopus) and Raccoon Dogs (Nyctereutes procyonoides) in China. PloS one 2015, 10, (11), e0142611.

246. Zhang, X. X.; Cong, W.; Lou, Z. L.; Ma, J. G.; Zheng, W. B.; Yao, Q. X.; Zhao, Q.; Zhu, X. Q., Prevalence, risk factors and multilocus genotyping of *Enterocytozoon bieneusi* in farmed foxes (Vulpes lagopus), Northern China. Parasit Vectors 2016, 9, 72.

247. Santin, M.; Calero-Bernal, R.; Carmena, D.; Mateo, M.; Balseiro, A.; Barral, M.; Lima Barbero, J. F.; Habela, M. A., Molecular Characterization of *Enterocytozoon bieneusi* in Wild Carnivores in Spain. J Eukaryot Microbiol 2018, 65, (4), 468-474.

248. Ma, Y. Y.; Zou, Y.; Ma, Y. T.; Nie, L. B.; Xie, S. C.; Cong, W.; Xu, Q. M.; Zhu, X. Q., Molecular detection and genotype distribution of *Enterocytozoon bieneusi* in farmed silver foxes (Vulpes vulpes) and arctic foxes (Vulpes lagopus) in Shandong Province, eastern China. Parasitol Res 2020, 119, (1), 321-326.

249. Karim, M. R.; Yu, F.; Li, J.; Li, J.; Zhang, L.; Wang, R.; Rume, F. I.; Jian, F.; Zhang, S.; Ning, C., First molecular characterization of enteric protozoa and the human pathogenic microsporidian, *Enterocytozoon bieneusi*, in captive snakes in China. Parasitol Res 2014, 113, (8), 3041-8.

250. Zhao, G. H.; Du, S. Z.; Wang, H. B.; Hu, X. F.; Deng, M. J.; Yu, S. K.; Zhang, L. X.; Zhu, X. Q., First report of zoonotic Cryptosporidium spp., Giardia intestinalis and *Enterocytozoon bieneusi* in golden takins (Budorcas taxicolor bedfordi). Infect Genet Evol 2015, 34, 394-401.

251. Xu, C.; Ma, X.; Zhang, H.; Zhang, X. X.; Zhao, J. P.; Ba, H. X.; Rui, D.; Xing, X. M.; Wang, Q. K.; Zhao, Q., Prevalence, risk factors and molecular characterization of *Enterocytozoon bieneusi* in raccoon dogs (Nyctereutes procyonoides) in five provinces of Northern China. Acta Trop 2016, 161, 68-72.

252. Yang, Z.; Zhao, W.; Shen, Y.; Zhang, W.; Shi, Y.; Ren, G.; Yang, D.; Ling, H.; Yang, F.; Liu, A.; Cao, J., Subtyping of Cryptosporidium cuniculus and genotyping of *Enterocytozoon bieneusi* in rabbits in two farms in Heilongjiang Province, China. Parasite 2016, 23, 52.

253. Zhang, X. X.; Jiang, J.; Cai, Y. N.; Wang, C. F.; Xu, P.; Yang, G. L.; Zhao, Q., Molecular Characterization of *Enterocytozoon bieneusi* in Domestic Rabbits (Oryctolagus cuniculus) in Northeastern China. Korean J Parasitol 2016, 54, (1), 81-5.

254. Lesnianska, K.; Perec-Matysiak, A.; Hildebrand, J.; Bunkowska-Gawlik, K.; Pirog, A.; Popiolek, M., Cryptosporidium spp. and *Enterocytozoon bieneusi* in introduced raccoons (Procyon lotor)-first evidence from Poland and Germany. Parasitol Res 2016, 115, (12), 4535-4541.

255. Tian, G. R.; Zhao, G. H.; Du, S. Z.; Hu, X. F.; Wang, H. B.; Zhang, L. X.; Yu, S. K., First report of *Enterocytozoon bieneusi* from giant pandas (Ailuropoda melanoleuca) and red pandas (Ailurus fulgens) in China. Infect Genet Evol 2015, 34, 32-5.

256. Zhang, X. X.; Jiang, R. L.; Ma, J. G.; Xu, C.; Zhao, Q.; Hou, G.; Liu, G. H., *Enterocytozoon bieneusi* in Minks (Neovison vison) in Northern China: A Public Health Concern. Front Microbiol 2018, 9, 1221.

257. Wu, J.; Han, J. Q.; Shi, L. Q.; Zou, Y.; Li, Z.; Yang, J. F.; Huang, C. Q.; Zou, F. C., Prevalence, genotypes, and risk factors of *Enterocytozoon bieneusi* in Asiatic black bear (Ursus thibetanus) in Yunnan Province, Southwestern China. Parasitol Res 2018, 117, (4), 1139-1145.

258. Lee, S. H.; Oem, J. K.; Lee, S. M.; Son, K.; Jo, S. D.; Kwak, D., Molecular detection of *Enterocytozoon bieneusi* from bats in South Korea. Medical mycology 2018, 56, (8), 1033-1037.

259. Cong, W.; Qin, S. Y.; Meng, Q. F., Molecular characterization and new genotypes of *Enterocytozoon bieneusi* in minks (Neovison vison) in China. Parasite 2018, 25, 34.

260. Zhang, Y.; Koehler, A. V.; Wang, T.; Haydon, S. R.; Gasser, R. B., New operational taxonomic units of Enterocytozoon in three marsupial species. Parasit Vectors 2018, 11, (1), 371.

261. Barton, C.; Snowden, K., Prevalence of Microsporidian Spores Shed by Asymptomatic Lovebirds: Evidence for a Potential Emerging Zoonosis. Journal of Avian Medicine and Surgery - J AVIAN MED SURG 2003, 17, 197-202.

262. Lobo, M. L.; Xiao, L.; Cama, V.; Magalhaes, N.; Antunes, F.; Matos, O., Identification of potentially human-pathogenic *Enterocytozoon bieneusi* genotypes in various birds. Appl Environ Microbiol 2006, 72, (11), 7380-2.

263. Müller, M. G.; Kinne, J.; Schuster, R. K.; Walochnik, J., Outbreak of microsporidiosis caused by *Enterocytozoon bieneusi* in falcons. Veterinary parasitology 2008, 152, (1-2), 67-78.

264. Kasicková, D.; Sak, B.; Kvác, M.; Ditrich, O., Sources of potentially infectious human microsporidia: molecular characterisation of microsporidia isolates from exotic birds in the Czech Republic, prevalence study and importance of birds in epidemiology of the human microsporidial infections. Veterinary parasitology 2009, 165, (1-2), 125-30.

265. Malcekova, B.; Valencakova A Fau - Luptakova, L.; Luptakova L Fau - Molnar, L.; Molnar L Fau - Ravaszova, P.; Ravaszova P Fau - Novotny, F.; Novotny, F., First detection and genotyping of Encephalitozoon cuniculi in a new host species, gyrfalcon (Falco rusticolus). (1432-1955 (Electronic)).

266. Malcekova, B.; Valencakova, A.; Luptakova, L.; Molnar, L.; Ravaszova, P.; Novotny, F., First detection and genotyping of Encephalitozoon cuniculi in a new host species, gyrfalcon (Falco rusticolus). Parasitology research 2011, 108, (6), 1479-82.

267. Lee, S. Y.; Lee, S. S.; Lyoo, Y. S.; Park, H. M., DNA detection and genotypic identification of potentially human-pathogenic microsporidia from asymptomatic pet parrots in South Korea as a risk factor for zoonotic emergence. Applied and environmental microbiology 2011, 77, (23), 8442-4.

268. Lallo, M. A.; Calábria P Fau - Milanelo, L.; Milanelo, L., Encephalitozoon and Enterocytozoon (Microsporidia) spores in stool from pigeons and exotic birds: microsporidia spores in birds. (1873-2550 (Electronic)).

269. Słodkowicz-Kowalska, A.; Graczyk Tk Fau - Nowosad, A.; Nowosad A Fau - Majewska, A. C.; Majewska, A. C., First detection of microsporidia in raised pigeons in Poland. (1898-2263 (Electronic)).

270. Malčeková, B.; Valenčáková A Fau - Molnár, L.; Molnár L Fau - Kočišová, A.; Kočišová, A., First detection and genotyping of human-associated microsporidia in wild waterfowl of Slovakia. (1896-1851 (Electronic)).

271. Pirestani M Fau - Sadraei, J.; Sadraei J Fau - Forouzandeh, M.; Forouzandeh, M., Molecular characterization and genotyping of human related microsporidia in free-ranging and captive pigeons of Tehran, Iran. (1567-7257 (Electronic)).

272. Li, J.; Qi, M.; Chang, Y.; Wang, R.; Li, T.; Dong, H.; Zhang, L., Molecular Characterization of Cryptosporidium spp., Giardia duodenalis, and *Enterocytozoon bieneusi* in Captive Wildlife at Zhengzhou Zoo, China. (1550-7408 (Electronic)).

273. Zhao, W.; Yu, S.; Yang, Z.; Zhang, Y.; Zhang, L.; Wang, R.; Zhang, W.; Yang, F.; Liu, A., Genotyping of *Enterocytozoon bieneusi* (Microsporidia) isolated from various birds in China. (1567-7257 (Electronic)).

274. Rzymski, P.; Slodkowicz-Kowalska, A.; Klimaszyk, P.; Solarczyk, P.; Poniedzialek, B., Screening of protozoan and microsporidian parasites in feces of great cormorant (Phalacrocorax carbo). Environmental science and pollution research international 2017, 24, (10), 9813-9819.

275. Deng, L.; Yue, C. J.; Chai, Y. J.; Wang, W. Y.; Su, X. Y.; Zhou, Z. Y.; Wang, L. Q.; Li, L. Y.; Liu, H. F.; Zhong, Z. J.; Cao, S. Z.; Hu, Y. C.; Fu, H. L.; Peng, G. N., New genotypes and molecular characterization of Enterocytozoon bieneusi in pet birds in Southwestern China. International journal for parasitology. Parasites and wildlife 2019, 10, 164-169.

276. Rzymski, P.; Słodkowicz-Kowalska, A.; Klimaszyk, P.; Solarczyk, P.; Poniedziałek, B., Screening of protozoan and microsporidian parasites in feces of great cormorant (Phalacrocorax carbo). (1614-7499 (Electronic)).

277. Deng, L.; Yue, C. J.; Chai, Y. J.; Wang, W. Y.; Su, X. Y.; Zhou, Z. Y.; Wang, L. Q.; Li, L. Y.; Liu, H. F.; Zhong, Z. J.; Cao, S. Z.; Hu, Y. C.; Fu, H. L.; Peng, G. N., New genotypes and molecular characterization of *Enterocytozoon bieneusi* in pet birds in Southwestern China. (2213-2244 (Print)).

278. Zhao, W.; Zhou, H. H.; Ma, T. M.; Cao, J.; Lu, G.; Shen, Y. J., PCR-Based Detection of Cryptosporidium spp. and *Enterocytozoon bieneusi* in Farm-Raised and Free-Ranging Geese (Anser anser f. domestica) From Hainan Province of China: Natural Infection Rate and the Species or Genotype Distribution. (2235-2988 (Electronic)).

279. Tavalla, M.; Mardani-Kateki, M.; Abdizadeh, R.; Soltani, S.; Saki, J., Molecular diagnosis of potentially human pathogenic *Enterocytozoon bieneusi* and Encephalitozoon species in exotic birds in Southwestern Iran. (1876-035X (Electronic)).

280. Feng, S. Y.; Chang, H.; Luo, J.; Huang, J. J.; He, H. X., First report of *Enterocytozoon bieneusi* and Cryptosporidium spp. in peafowl (Pavo cristatus) in China. (2213-2244 (Print)).

281. Galván, A. L.; Magnet, A.; Izquierdo, F.; Fenoy, S.; Rueda, C.; Fernández Vadillo, C.; Henriques-Gil, N.; del Aguila, C., Molecular characterization of human-pathogenic microsporidia and Cyclospora cayetanensis isolated from various water sources in Spain: a year-long longitudinal study. Applied and environmental microbiology 2013, 79, (2), 449-59.

282. Zhao W, Y. S., Yang Z, Zhang Y, Zhang L, Wang R, Zhang W, Yang F, Liu A, Genotyping of *Enterocytozoon bieneusi* (Microsporidia) isolated from various birds in China. Infect Genet Evol 2016.

283. Ma, J.; Feng, Y.; Hu, Y.; Villegas, E. N.; Xiao, L., Human infective potential of Cryptosporidium spp., Giardia duodenalis and *Enterocytozoon bieneusi* in urban wastewater treatment plant effluents. (1477-8920 (Print)).

284. Ye, J.; Ji, Y.; Xu, J.; Ma, K.; Yang, X., Zoonotic *Enterocytozoon bieneusi* in raw wastewater in Zhengzhou, China. Folia parasitologica 2017, 64.

285. Huang, C.; Hu, Y.; Wang, L.; Wang, Y.; Li, N.; Guo, Y.; Feng, Y.; Xiao, L., Environmental Transport of Emerging Human-Pathogenic Cryptosporidium Species and Subtypes through Combined Sewer Overflow and Wastewater. Applied and environmental microbiology 2017, 83, (16).

286. Yamashiro, S.; Fiuza, V.; Teixeira, A.; Branco, N.; Levy, C. E.; Castro, I.; Franco, R. M. B., *Enterocytozoon bieneusi* detected by molecular methods in raw sewage and treated effluent from a combined system in Brazil. Mem Inst Oswaldo Cruz 2017, 112, (6), 403-410.

287. Moss, J. A.; Snyder, R. A., Biofilms for Monitoring Presence of Microsporidia in Environmental Water. J Eukaryot Microbiol 2017, 64, (4), 533-538.

288. Chen, J. S.; Hsu, B. M.; Tsai, H. C.; Chen, Y. P.; Huang, T. Y.; Li, K. Y.; Ji, D. D.; Lee, H. S., Molecular surveillance of Vittaforma-like microsporidia by a small-volume procedure in drinking water source in Taiwan: evidence for diverse and emergent pathogens. Environmental science and pollution research international 2018, 25, (19), 18823-18837.
